# A lithium–aluminium heterobimetallic dimetallocene

**DOI:** 10.1038/s41557-024-01531-y

**Published:** 2024-05-14

**Authors:** Inga-Alexandra Bischoff, Sergi Danés, Philipp Thoni, Bernd Morgenstern, Diego M. Andrada, Carsten Müller, Jessica Lambert, Elias C. J. Gießelmann, Michael Zimmer, André Schäfer

**Affiliations:** Department of Chemistry, Faculty of Natural Sciences and Technology, Saarland University, Saarbrücken, Germany

**Keywords:** Chemical bonding, Chemical bonding, Chemical bonding

## Abstract

Homobimetallic dimetallocenes exhibiting two identical metal atoms sandwiched between two η^5^ bonded cyclopentadienyl rings is a narrow class of compounds, with representative examples being dizincocene and diberyllocene. Here we report the synthesis and structural characterization of a heterobimetallic dimetallocene, accessible through heterocoupling of lithium and aluminylene fragments with pentaisopropylcyclopentadienyl ligands. The Al–Li bond features a high ionic character and profits from attractive dispersion interactions between the isopropyl groups of the cyclopentadienyl ligands. A key synthetic step is the isolation of a cyclopentadienylaluminylene monomer, which also enables the structural characterization of this species. In addition to their structural authentication by single-crystal X-ray diffraction analysis, both compounds were characterized by multinuclear NMR spectroscopy in solution and in the solid state. Furthermore, reactivity studies of the lithium–aluminium heterobimetallic dimetallocene with an N-heterocyclic carbene and different heteroallenes were performed and show that the Al–Li bond is easily cleaved.

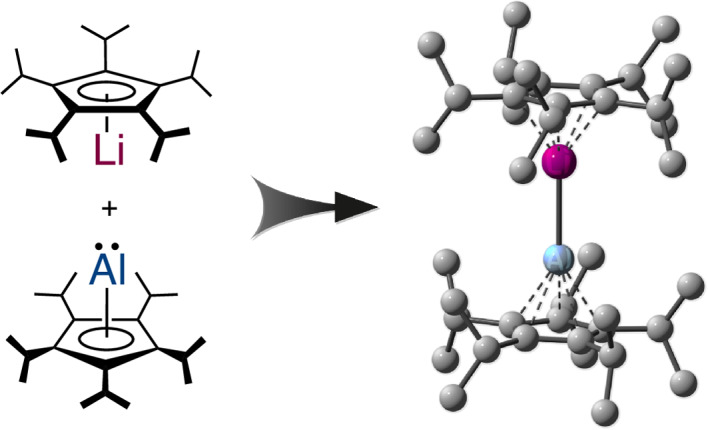

## Abstract (web summary)

Dimetallocenes are a narrow class of compounds represented by the homobimetallic examples dizincocene and diberyllocene. Now a heterobimetallic dimetallocene featuring lithium and aluminium centres has been synthesized. The Al–Li bond is shown to cleave upon reaction with N-heterocyclic carbenes and heteroallenes.

## Main

**Fig. 1:**
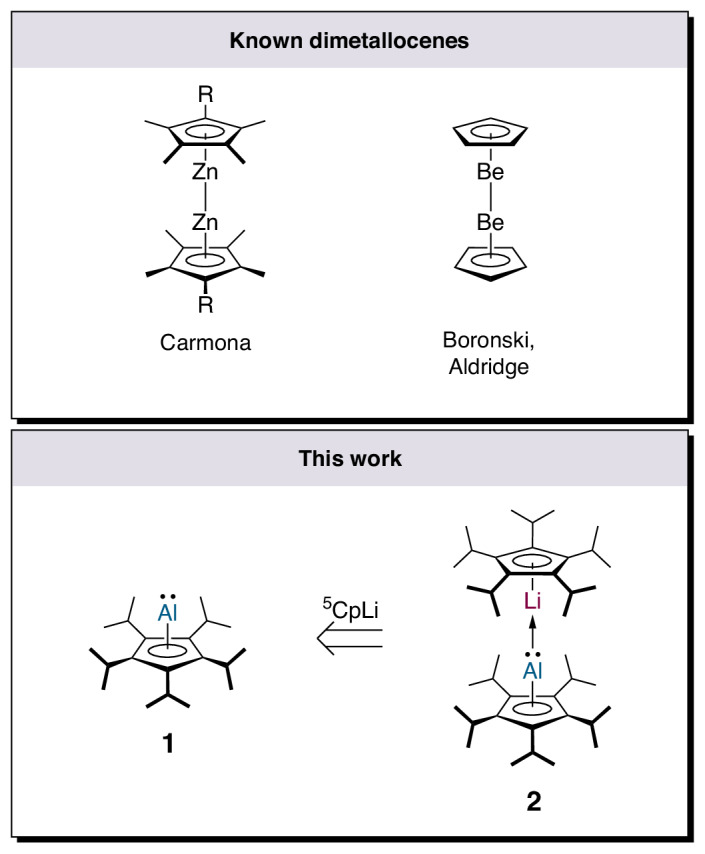
Overview of dimetallocenes. Top: reported homobimetallic dimetallocenes. Bottom: synthetic strategy to the lithium–aluminium heterobimetallic dimetallocene (this work).

The discovery of ferrocene has undoubtedly revolutionized organometallic chemistry, as metallocenes have shaped various areas of chemistry and have become standard textbook knowledge, nowadays^1–3^. Not only has their unexpected bonding situation revolutionized bonding theory by introducing the concept of a ‘sandwich complex’, but their properties have also attracted much attention and made them an extremely important class of compounds for various fields, including catalysis, materials chemistry, bio-medical applications and beyond^4–9^. Over the years, metallocene-type compounds—species of the general formula ‘(η^5^-Cp)_2_[M]’ (Cp = cyclopentadienyl; [M] = metal centre)—have been described for many elements across the periodic table^10–16^. Unlike these monometallic derivatives, the term dimetallocene refers to very rare sandwich complexes in which two metal atoms are bonded between the η^5^-coordinated Cp ligands arranged in linear/coplanar fashion and interlinked by a metal–metal bond. The synthesis of homobimetallic decamethyldizincocene (Cp*_2_Zn_2_) by Carmona and co-workers in 2004 was a paradigm-shifting milestone of modern organometallic chemistry^17,18^, as preceding reports on dimetallocenes did not provide suitable evidence and were subsequently shown to be erroneous^19–21^. Additionally, bimetallic complexes in which two metal centres are bridged by halides, hydrides or hydroxy, carbonyl, aryl or alkyl groups are well known^22,23^. Nevertheless, different transition metals and main-group elements have been theoretically predicted to form stable dimetallocenes^24–28^, yet dizincocene remained the only experimentally characterized example until very recently, when the likewise homobimetallic diberyllocene (Cp_2_Be_2_) was described by Boronski and Aldridge (Fig. 1)^29^. On the other hand, dimetallocenes of p-block elements are still unknown, although numerous attempts to isolate a dimetallocene of silicon were made but were all unsuccessful due to disproportionation of the alleged decamethyldisilicocene into decamethylsilicocene (Cp*_2_Si) and silicon(0) (refs. ^30,31^). Notably, heterobimetallic dimetallocenes have remained elusive so far, although they have been theoretically studied since nearly two decades^32–34^. Combinations of group 1 and group 13 metals were proposed as intermediates, but—despite considerable efforts—have never been detected let alone isolated. For example, Timoshkin and Schaefer speculated that the experimental observation of Cp^Bn5^Li in the reaction of Cp^Bn5^Al and Cp*Li might be explained by the occurrence of a weakly bonded donor–acceptor complex of the type Cp^Bn5^Al→LiCp^Bn5^ (refs. ^32,35^).

Our synthetic strategy towards a heterobimetallic group 1 group 13 dimetallocene relied on the isolation of a cyclopentadienylaluminylene (Fig. 1), a species whose crystal structure has been elusive for almost three decades^36^. Schnöckel’s report of (pentamethylcyclopentadienyl)aluminium(I) in 1991 demonstrated the ability of cyclopentadienyl ligands to stabilize aluminium(I) centres, which have since become common in low-valent aluminium chemistry^37,38^. Noteworthily, this compound exists in tetrameric form in the solid state and in solution at room temperature, while monomeric cyclopentadienylaluminylenes are accessible at elevated temperatures, or with more bulky substitution patterns on the Cp moiety^36^. The isolation of a monomeric cyclopentadienylaluminylene was reported recently by Braunschweig and co-workers, but its monomeric nature was confirmed solely NMR spectroscopically from the crude product in solution^39^. Thus, structural authentication of a monomeric cyclopentadienylaluminylene has remained elusive, although a few monomeric aluminylenes with σ-bonded substituents have been structurally characterized^40–47^.

In this Article, we report the synthesis, isolation and structural characterization of a monomeric cyclopentadienylaluminylene taking advantage of the sterically very demanding pentaisopropylcyclopentadienyl (^5^Cp) ligand^23^. Even more importantly, reaction of (^5^Cp)aluminylene **1** with (^5^Cp)lithium, ^5^CpLi, results in the formation of the heterobimetallic dimetallocene **2** with a unique dative metal–metal bond.

## Results and discussion

### Aluminylene 1

**Fig. 2:**
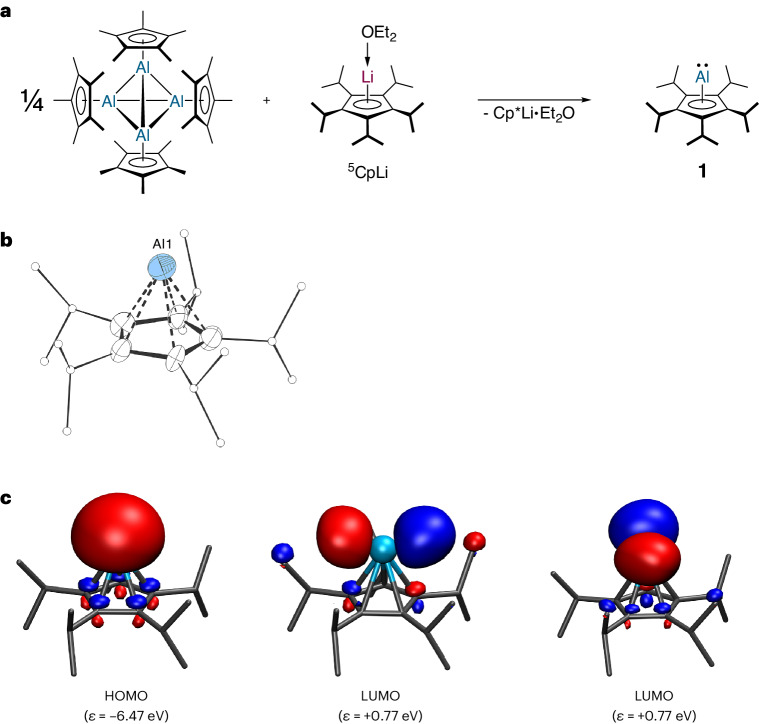
(^5^Cp)aluminylene 1. **a**, Synthesis of aluminylene **1**. **b**, The molecular structure of **1** in the crystal (displacement ellipsoids at 50% probability level, hydrogen atoms omitted for clarity, ^*i*^Pr groups drawn as ball-and-stick models). Selected experimental and theoretical [M06-2X/def2-SVP] bond lengths: Al1–Cp^cent^: 196.71(6) [199.1] pm. **c**, Selected Kohn–Sham frontier molecular orbital contours of **1** (M06-2X/def2-TZVPP//M06-2X/def2-SVP; isodensity 0.05 a.u.). *ε*, orbital energy.

Following the report of Schnöckel and co-workers, the (pentamethylcyclopentadienyl)aluminium(I) tetramer can be utilized as a precursor for the synthesis of cyclopentadienylaluminylenes^38,48^; we reacted it with (^5^Cp)lithium diethyletherate, ^5^CpLi∙OEt_2_, and obtained the corresponding (^5^Cp)aluminylene, **1** (Fig. 2a).

As shown by the groups of Schnöckel and Braunschweig, monomeric cyclopentadienylaluminylenes have ^27^Al NMR chemical shifts in the range of −150 to −170 ppm, while the tetrameric aggregates exhibit more downfield-shifted resonances, usually in the range of −60 to −110 ppm (refs. ^36,39^). This is due to increased π type bonding interactions between the aluminium atom and the Cp ligand in the monomeric species, which results in an energetically higher lying lowest unoccupied molecular orbital (LUMO) and, thus, a larger highest occupied molecular orbital (HOMO)–LUMO gap and smaller paramagnetic contribution to the ^27^Al NMR chemical shift^49,50^. Accordingly, **1** exhibits a ^27^Al NMR chemical shift of δ^27^Al{^1^H}(C_6_D_6_) = −154 (*ω*_½_ = 521 Hz) in solution and of δ^27^Al(SPE/MAS(13 kHz)) = −154 (SPE = single pulse excitation; MAS = magic-angle spinning) in the solid state at ambient temperatures, clearly indicating its monomeric nature both in the solid state and in solution (Supplementary Figs. 3 and 4). Crystals of **1**, suitable for single-crystal X-ray diffraction (XRD), were obtained by sublimation of the compound in vacuum at 323 K. The crystal structure of **1** reveals well-separated, monomeric (^5^Cp)aluminium moieties (Fig. 2b and Supplementary Fig. 36). The closest contact from the aluminium centre to a neighbouring molecule is to an H atom of a methyl group, which is 316.45(5) pm. The aluminium atom is η^5^-coordinated by the cyclopentadienyl moiety leading to an overall pentagonal pyramidal structure. Following polyhedral skeletal electron pair theory, more commonly referred to as the Wade–Mingos rules^51–53^, **1** can be classified as a *nido* cluster. The Al–Cp^centroid^ and Al–C^Cp^ bond lengths in **1** are slightly shorter than those in {Cp*Al}_4_ (Table 1). This originates from the increased Al–Cp bonding interaction in **1** due to its monomeric nature, as also apparent from the ^27^Al NMR chemical shift (vide supra)^49,50^. We analysed the electronic structure of **1** within the density-functional theory (DFT) framework, whereby the equilibrium geometry is in very good agreement with the structure determined by single-crystal XRD (Fig. 2b), with the Al–Cp^centroid^ distance being slightly longer than those observed experimentally. Similar to former theoretical calculations^54,55^, the Kohn–Sham frontier molecular orbitals of **1** (Fig. 2c) consist of a lone pair at the Al atom (HOMO) and two degenerated 3*p* orbitals (LUMO) at the Al atom, respectively. Moreover, the natural population analysis (NPA) and Bader’s quantum theory of atoms in molecules (QTAIM)^56^ show that the aluminium atom is positively charged by +0.70 a.u. (NPA)/+0.81 a.u. (QTAIM) (Supplementary Fig. 44), indicating a relatively ionic bonding interaction between the aluminium atom and the Cp ligand.

**Table 1:** Selected bond length for {Cp*Al}_4_; 1; 1∙AlBr_3_; 1∙W(CO)_5_ and 2

Compound	Al–C^Cp^ (pm)	Al–Cp^centroid^ (pm)
(Cp*Al)_4_ (ref. ^32^)	229(1) to 237(1)	199.8(2) to 203.2(3)
^5^CpAl, **1**	226.9(7) to 235.7(8)	196.7(6)
^5^CpAl→AlBr_3_, **1**∙**AlBr**_**3**_	215.5(2) to 216.4(3)	178.3(9)
^5^CpAl→W(CO)_5_, **1**∙**W(CO)**_**5**_	219.2(8) to 221.5(7)	183.5(1)
^5^CpAl→Li^5^Cp, **2**	222.1(1) to 226.2(1)	188.6(1); 188.8(3)

### Aluminylene complexes 1∙AlBr_3_ and 1∙W(CO)_5_

Due to the lone pair of the aluminium atom, sterically less demanding cyclopentadienylaluminylenes are known to act as donors towards electron-deficient acceptors^39,44,47,57–60^. Thus, to explore the reactivity of the sterically very encumbered **1**, we initially investigated the donor ability of **1** towards electrophiles, which are known to coordinate to Cp*Al and Cp′′′Al. Treatment of **1** with one equivalent of aluminium tribromide indeed affords the corresponding adduct **1∙AlBr**_**3**_. With tungsten hexacarbonyl under ultraviolet irradiation, the corresponding aluminylene tungsten complex **1∙W(CO)**_**5**_ was formed. Single crystals of both compounds were obtained and allowed for structural characterization in the solid state by XRD (Supplementary Figs. 37 and 38). Complex **1∙AlBr**_**3**_ exhibits an Al–Al bond length of 255.5(1) pm, which is similar to the bond in Cp′′′Al→AlBr_3_ (255.4(1) pm)^39^, suggesting similar donor abilities of Cp′′′Al and **1**. A cyclopentadienylaluminylene tungsten carbonyl complex had not been described previously, although the analogous Cp*Al→Cr(CO)_5_ complex and other organoaluminium(I) tungsten carbonyl complexes are known^44,59^. The Al–W bond length in **1∙W(CO)**_**5**_ amounts to 258.5(1) pm, which is longer than in a carbazolylaluminylene tungsten pentacarbonyl complex (253.6(1) pm)^44^, but shorter than those reported for (TMEDA)(R)Al→W(CO)_5_-type compounds (TMEDA = tetramethylethylenediamine; R = Cl: 264.5(2) pm; R = Et: 267.0(1) pm; R = ^*t*^Bu: 274.1(4) pm)^60^. The carbonyl vibration bands in the infra-red spectrum of **1∙W(CO)**_**5**_ are similar to those of the carbazolylaluminylene tungsten complex, hinting to equal donor strength (Supplementary Fig. 30). Interestingly, the Al–Cp^centroid^ distances in **1∙AlBr**_**3**_ (178.3(9) pm) and **1∙W(CO)**_**5**_ (183.5(6) pm) are substantially shortened compared with what is observed in uncomplexed **1** (196.7(6) pm), which is a result of the electron deficiency at the aluminium(I) centre influenced by the electron-withdrawal power of the coordinated metal fragment, and the corresponding compensation by the ^5^Cp ligand.

### Dimetallocene 2

**Fig. 3:**
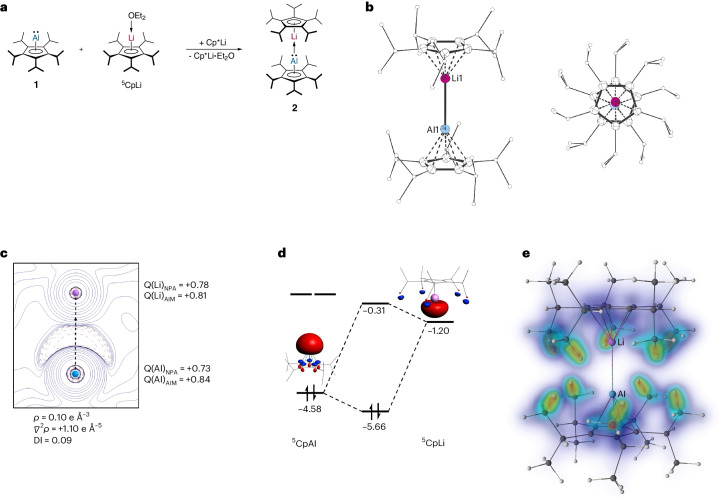
Lithium–aluminium dimetallocene 2. **a**, Synthesis of dimetallocene **2**. **b**, The molecular structure of **2** in the crystal (side view and top view, displacement ellipsoids at 50% probability level, hydrogen atoms omitted for clarity, ^*i*^Pr groups drawn as ball-and-stick models). Selected experimental and theoretical [M06-2X/def2-SVP] bond lengths: Li1–Cp^cent^: 176.2(3)–176.6(1) [170.8] pm, Al1–Cp^cent^: 188.6(1)–189.3(1) [190.9] pm, Al1–Li1: 261.5(2) [265.8] pm. **c**, Laplacian distribution *∇*^2^*ρ*(*r*) of **2** (M06-2X/de2-TZVPP//M06-2X/def2-SVP). Dashed red lines indicate areas of charge concentration (*∇*^2^*ρ*(*r*) < 0); solid blue lines indicate areas of charge depletion (*∇*^2^*ρ*(*r*) > 0) (bond ellipticity: 0.0). DI, delocalization index; Q, partial charge. **d**, Molecular orbital interaction diagram in eV for the Al–Li σ-bond in **2** (M06-2X/de2-TZVPP//M06-2X/def2-SVP; isodensity 0.05). **e**, DID plot (LMP2/cc-pVTZ). Orange/yellow/green zones indicate strong dispersion interactions, and blue/turquoise zones indicate weaker/diffuse contributions.

The clearly apparent ability of **1** to act as a donor ligand despite the bulky ^5^Cp group suggested it as an excellent candidate for the deliberate synthesis of a heterobimetallic dimetallocene, inspired by theoretical predictions, as well as reports of aluminylene lithium complexes with σ-bonded substituents^32–34,61^. Indeed, the reaction of **1** with one equivalent of ^5^CpLi (in the presence of Cp*Li as diethyl ether scavenger) resulted in the formation of lithium–aluminium heterobimetallic dimetallocene **2** (Fig. 3a). Single crystals of **2** were analysed by XRD unambiguously proving the structure of **2** in the solid state (Fig. 3b).

Two crystal structures of dimetallocene **2** could be obtained, co-crystalized with toluene or with 1,2-difluorobenzene. From toluene, **2** crystallizes in the triclinic space group *P*1 with one formula unit and two molecules of toluene per asymmetric unit. Due to the high symmetry of the molecule, a positional disorder of the Al and Li positions of 93:7 is observed. The ^5^Cp ligands are both bonded in η^5^ fashion and adopt a staggered conformation, interestingly unlike in dizincocene and diberyllocene, where eclipsed conformations are observed^17,18,29^. This might be caused by steric pressure and/or packing effects. The Al–Li bond length is 261.5(2) pm and, thus, substantially shorter than in ionic aluminyl lithium complexes, for example, (NON)Al→Li(Et_2_O)_2_/{(NON)Al→Li}_2_: 274.6(3) pm to 276.7(2) pm (NON = 4,5-bis(2,6-diisopropylanilido)-2,7-di-tert-butyl-9,9-dimethyl-xanthene) reported before. Noteworthily, these examples are not only not structurally related to **2** but are also ionic in nature (‘[RAl]^−^→[Li]^+^’), while **2** consists of two formally neutral fragments (‘RAl→LiR’) and is, therefore, essentially without precedents^62^. Nonetheless, the Al–Li bond length is in good agreement with the predicted sum of the covalent radii of Al and Li (∑*r*_cov_(Al + Li) = 259 pm)^63^. The ^7^Li and ^27^Al NMR chemical shifts of **2** in solution are δ^7^Li = −9.63 and δ^27^Al = −151 (*ω*_½_ = 1,139 Hz), which are only slightly different from the ^27^Al NMR chemical shift of **1** (δ^27^Al = −154) and the ^7^Li NMR chemical shift of ^5^CpLi∙OEt_2_ (δ^7^Li = −8.18), hinting at a weak Al–Li interaction. In the solid state, **2** reveals similar NMR chemical shifts (δ^7^Li(SPE/MAS(13 kHz)) = −8.9; δ^27^Al(SPE/MAS(13 kHz)) = −157), with the signal in the ^7^Li NMR spectrum split to a hexet with a coupling constant of ${1\atop}{\mathrm{J}}_{{7\atop}{\rm{L}}{\rm{i}}-{}^{27}{\rm{A}}{\rm{l}}}=102\,{\rm{Hz}}$, clearly reflecting the Al–Li bonding interaction (Supplementary Figs. 12 and 14).

To gain further insight into the electronic structure of **2**, we performed DFT calculations. As the nature of the Al–Li bond is of particular interest, we analysed the topology of the electron density with QTAIM^56^. The Bader analysis reveals a high charge concentration on the aluminium basin, which agrees with a lone pair (Fig. 3c). The bond critical point (BCP) of the bond path, which connects the Al with the Li atom, reveals low electron density (*ρ*(*r*)^BCP^ = 0.10 e Å^−3^) with a large positive Laplacian value (*∇*^2^*ρ*(*r*) = +1.10 e Å^−^^5^) and positively charged Al by +0.84 a.u. and Li by 0.81 a.u. Moreover, the delocalization index of the Al–Li pair is rather low (0.09), and NPA also reveals positively charged Al by +0.73 a.u. and Li by +0.78 a.u. These features are typically found in ionic interactions and are similar to other reported ionic aluminyl lithium complexes. For example, NBO-derived natural atomic charges of (NON)Al→Li(Et_2_O)_2_ are +0.69 for Al and +0.73 for Li (ref. ^61^). Additionally, QTAIM analysis of ionic (NON)Al→Li(Et_2_O)_2_ complexes reveals BCPs for the Al–Li bond with even lower electron densities of *ρ*(*r*)^BCP^ = 0.019 to 0.018 e Å^−3^ (refs. ^61,62^), indicating a more covalent character of the Al–Li interaction in **2**. The relatively higher stability of **2** compared with former predictions originates from attractive dispersion interactions of the ^5^Cp ligands, as shown by the energy decomposition analysis (EDA) method (Table 2 and Supplementary Table 3)^64^. For comparison, we also performed EDA of the theoretical Cp and Cp* derivatives, as well as for Carmona’s Cp*_2_Zn_2_ and Boronski–Aldridge’s Cp_2_Be_2_. The bond dissociation energy for the Al–Li bond in **2** (*D*_e_ = 93.4 kJ mol^−1^) is notably higher than for the Cp (*D*_e_ = 42.7 kJ mol^−^^1^) and Cp* (*D*_e_ = 55.8 kJ mol^−^^1^) analogues (Supplementary Table 3). An examination of the Δ*E*_int_ components for **2** suggests that dispersion interactions (Δ*E*_disp_ = −65.3 kJ mol^−^^1^, 37.7%) and electrostatic interaction (Δ*E*_elst_ = −64.5 kJ mol^−^^1^, 37.2%) are almost identical in magnitude, while the orbital interactions (Δ*E*_orb_ = −43.6 kJ mol^−^^1^, 21.5%) are smaller. The orbital interaction primarily corresponds to the donation of the lone pair of the aluminium atom of the ^5^CpAl fragment into the formally vacant orbital of the lithium atom of the ^5^CpLi fragment (Fig. 3d). The attractive dispersion interactions between the ^5^Cp ligands in **2** apparently play a major role to stabilize the dimetallocene, and are larger than in the Cp (−10.7 kJ mol^−^^1^) and Cp* (−16.8 kJ mol^−^^1^) analogues (Supplementary Table 3). To examine the origins of these dispersion forces, we performed energy partitioning, using local correlation methods LMP2/cc-pVTZ^65^. Within this method, the dipole–dipole moment interactions are quantified as the amplitude of pair excitations on localized orbitals of each fragment^65^. The dispersion interaction density (DID) plot (Fig. 3e)^66^ reveals dominating interactions between the isopropyl groups, namely C–H/C–H contacts, while the π–π interactions between the Cp rings are rather weak. For comparison, in Carmona’s Cp*Zn–ZnCp* and Boronski–Aldridge’s CpBe–BeCp, the homolytic fragmentations disclose more than three times larger dissociation energy (Cp*_2_Zn_2_: *D*_e_ = 302.9 kJ mol^−^^1^; Cp_2_Be_2_: 292.3 kJ mol^−^^1^) than in **2** (Table 2 and Supplementary Table 3)^25,67^. Furthermore, EDA of the dizincocene and diberyllocene shows that the stabilization interactions in these compounds are mainly the orbital (Cp*_2_Zn_2_: 41.2%; Cp_2_Be_2_: 32.0%) and electrostatic (Cp*_2_Zn_2_: 54.5%; Cp_2_Be_2_: 64.4%) terms, while attractive dispersion interactions play almost no role (Cp*_2_Zn_2_: 4.3%; Cp_2_Be_2_: 3.4%). These results clearly highlight the importance of the isopropyl groups of the ^5^Cp ligand to stabilize **2**. While **2** exhibits a polar dative bond, originating from the lone pair at the aluminium atom donating to a vacant orbital at the lithium atom (Supplementary Fig. 45), it is formally valence-isoelectronic to dizincocene and diberyllocene, which exhibit unpolar electron sharing bonds.

**Table 2:** EDA results (BP86-D3(BJ)/TZ2P//M06-2X/def2-SVP) for the Al–Li bonds in 2, CpAl→LiCp and Cp*Al→LiCp*, the Zn–Zn bond in Cp*Zn–ZnCp* and the Be–Be bond in CpBe–BeCp

	2 (^5^CpAl→Li^5^Cp)	Cp*Al→LiCp*	CpAl→LiCp	Cp*Zn–ZnCp*	CpBe–BeCp
Δ*E*_int_	−97.8	−59.2	−46.1	−308.6	−302.8
Δ*E*_Pauli_	75.6	28.7	24.9	207.6	215.0
Δ*E*_disp_^**a**^	−65.3 (37.7 %)	−16.8 (19.1%)	−10.7 (15.0 %)	−22.2 (4.3 %)	−17.7 (3.4 %)
Δ*E*_elst_^**a**^	−64.5 (37.2 %)	−43.0 (48.9 %)	−34.9 (49.2 %)	−281.5 (54.5 %)	−334.4 (64.4 %)
Δ*E*_orb_^**a**^	−43.6 (25.1 %)	−28.1 (31.9 %)	−25.4 (35.8 %)	−212.6 (41.2 %)	−165.7 (32.0 %)
Δ*E*_prep_	4.4	3.4	3.4	5.8	10.6
*D*_e_	93.4	55.8	42.7	302.9	292.3

Energies are given in kJ mol^−1^.

^a^The value in parentheses gives the percentage contribution to the total attractive interactions Δ*E*_elst_ + Δ*E*_orb_ + Δ*E*_disp_.

### Reactivity studies of dimetallocene 2

**Fig. 4:**
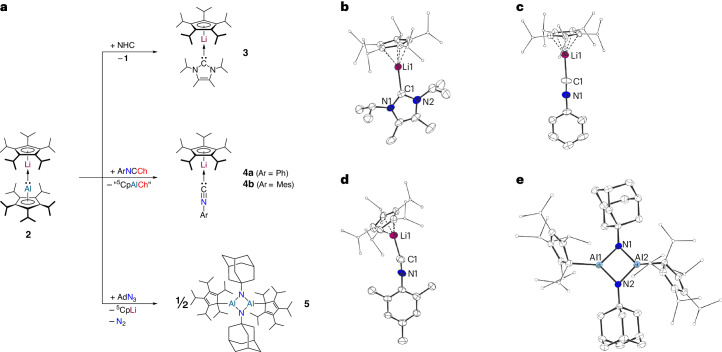
Reactivity of dimetallocene 2. **a**, Reaction of **2** with an NHC to give **3**, PhNCO and MesNCS to give **4a**,**b**, and AdN_3_ to give **5**. **b**, The molecular structure of **3** in the crystal. **c**, The molecular structure of **4a** in the crystal. **d**, The molecular structure of **4b** in the crystal. **e**, The molecular structure of **5** in the crystal (displacement ellipsoids at 50% probability level, hydrogen atoms omitted for clarity, ^*i*^Pr groups drawn as ball-and-stick models). Selected experimental bond lengths: **3**: Li1–C1: 216.6(2) pm, Li1–Cp^cent^: 184.9(2) pm; **4a**: C1–Li1: 210.4(2) pm, Li1–Cp^cent^: 170.7(5) pm; **4b**: C1–Li1: 210.0(8) pm, Li1–Cp^cent^: 153.1(7) pm; **5**: Al1/2–N1/2: 181.7(2)–182.3(2) pm, Al1/2–C^Cp^: 202.5(3)/202.8(3) pm, N1/2–C^Ad^: 146.5(3)/147.3(3) pm.

The computational investigations of **2** suggested that the Al–Li bond in **2** is fairly weak, compared with the metal–metal bond in dizincocene. To investigate this experimentally, we reacted **2** with an N-heterocyclic carbene (NHC), as in the case of decamethyldizincocene coordination of the NHC to one of the zinc atoms is observed, without cleavage of the Zn–Zn bond^68^. In contrast, a cleavage of the Al–Li bond in **2** is observed and the ^5^CpLi**∙**NHC complex **3** was isolated (Fig. 4b), which agrees with the DFT calculations that suggested the Al–Li bond to be rather weak and enforced by attractive dispersion interactions (vide supra). **3** exhibits a δ^7^Li shift of −9.07 ppm, which is similar to other cyclopentadienyl lithium NHC complexes^69,70^, as well as a Li–C1 bond length of 216.6(2) pm and a Li–Cp^centroid^ distance of 184.9(2) pm, which are in the same range as in other cyclopentadienyl lithium NHC complexes^69,70^. Next, **2** was reacted with three different heteroallenes, namely phenylisocyanate, mesitylisothiocyanate and 1-azidoadamantane. These reactions did also result in cleavage of the Al–Li bond, as complexes ^5^CpLi**∙**CNPh, **4a**, ^5^CpLi**∙**CNMes, **4b**, and {^5^CpAlNAd}_2_, **5**, were formed and crystals suitable for single-crystal XRD were obtained (Fig. 4c–e). The Li–C1 bond lengths of 210.4(2) pm (**4a**) and 210.0(8) pm (**4b**) are similar to other lithium isocyanide complexes^71^, and the bond lengths in **5** are relatively similar to an analogue complex reported by Braunschweig^39^. The chalcogen-transfer products in the formation of **4a** and **4b** eluded isolation and characterization, but based on DFT calculations (Supplementary Fig. 47) and previous reports of trimeric {Cp′′′AlO}_3_ (ref. ^39^), dimeric or trimeric compounds of the type {^5^CpAlCh}_*n*_ might be formed. We also performed control experiments in which we reacted aluminylene **1** with phenylisocyanate and mesitylisothiocyanate, but these experiments only yielded large amounts of pentaisopropylcyclopentadiene (^5^CpH) yet no isolatable amount of any {^5^CpAlCh}_*n*_ species. Interestingly, treatment of **1** with 1-azidoadamantane did not result in the formation of **5**, but gave a mixture of products also containing large amounts of ^5^CpH, indicating that the reactivity of aluminylene **1** and dimetallocene **2** towards 1-azidoadamantane differs.

## Conclusion

With the isolation of a monomeric cyclopentadienylaluminylene, **1**, the stage was set for the synthesis of a heterobimetallic dimetallocene. The lithium–aluminium dimetallocene **2**, while formally valence-isoelectronic to dizincocene and diberyllocene, exhibits a highly polar Al–Li bond, which is enforced by attractive dispersion interactions. As the Al–Li bond is relatively weak, it can be cleaved easily by donor molecules such as an NHC or in reactions with heteroallenes, such as phenylisocyanate, mesitylisothiocyanate and 1-azidoadamantane. These reactions resulted in the formation of (^5^Cp)lithium complexes **3**, **4** and dialumazine **5**. The cleavage of the Al–Li bond is in sharp contrast to the related valence-isoelectronic dizincocene, in which the Zn–Zn bond is perpetuated upon coordination of an NHC.

## Online content

Any methods, additional references, Nature Portfolio reporting summaries, source data, extended data, supplementary information, acknowledgements, peer review information; details of author contributions and competing interests; and statements of data and code availability are available at 10.1038/s41557-024-01531-y.

## Supplementary information

Supplementary Information.

Supplementary Data 1 Crystallographic data for compound **1**; CCDC reference no. 2279422.

Supplementary Data 2 Crystallographic data for compound **1∙AlBr**_**3**_; CCDC reference no. 2279423.

Supplementary Data 3 Crystallographic data for compound **1∙W(CO)**_**5**_; CCDC reference no. 2279424.

Supplementary Data 4 Crystallographic data for compound **2** co-crystallized with 1,2-difluorobenzene; CCDC reference no. 2324314.

Supplementary Data 5 Crystallographic data for compound **2** co-crystallized with toluene; CCDC reference no. 2279425.

Supplementary Data 6 Crystallographic data for compound **3**; CCDC reference no. 2324317.

Supplementary Data 7 Crystallographic data for compound **4a**; CCDC reference no. 2324316.

Supplementary Data 8 Crystallographic data for compound **4b**; CCDC reference no. 2338161.

Supplementary Data 9 Crystallographic data for compound **5**; CCDC reference no. 2338185.

Supplementary Data 10 DFT coordinates of the optimized structures.

## Figures and Tables

**Extended Data Fig. 1:**
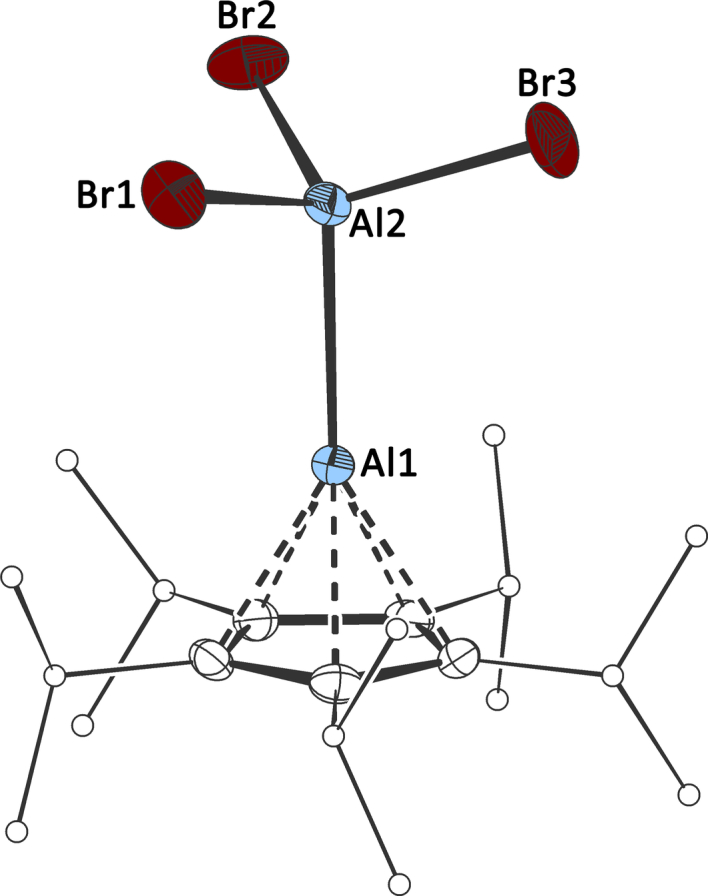
Molecular structure of 1∙AlBr_**3**_. Molecular structure of **1∙AlBr**_**3**_ in the crystal (displacement ellipsoids at 50% probability level, H atoms omitted for clarity, ^*i*^Pr groups drawn as ball-and-stick models).

**Extended Data Fig. 2:**
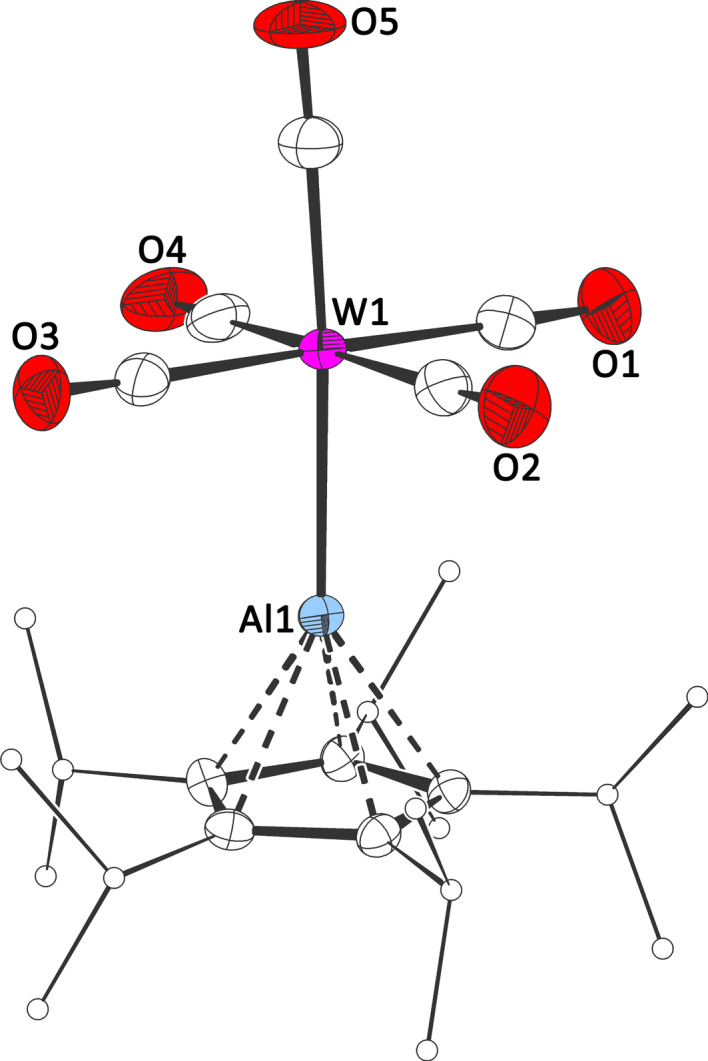
Molecular structure of 1∙W(CO)_**5**_. Molecular structure of **1∙W(CO)**_**5**_ in the crystal (displacement ellipsoids at 50% probability level, H atoms omitted for clarity, ^*i*^Pr groups drawn as ball-and-stick models).

**Extended Data Fig. 3:**
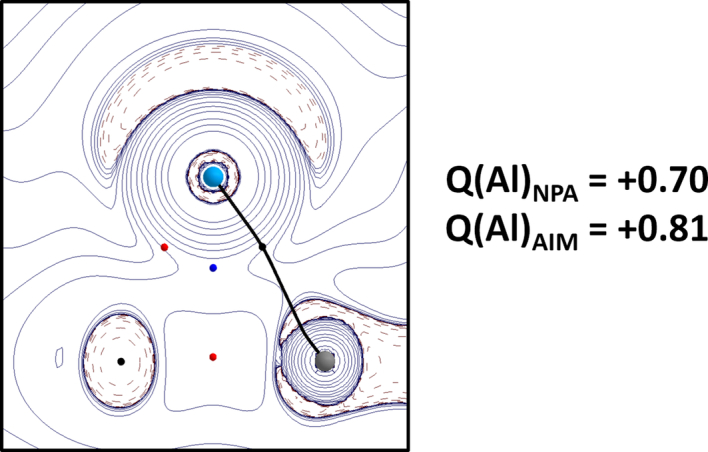
Laplacian distribution of the electron density of **1** (contour line diagrams of the Laplacian distribution ∇^2^ρ(r) in the Al–C–C plane. Dashed red lines indicate areas of charge concentration (∇^2^ρ(r)<0), solid blue lines show areas of charge depletion (∇^2^ρ(r)>0). Thick solid lines connecting the atomic nuclei are bond paths and small dots are the critical points, with bond critical points in black, ring critical points in red and cage critical point in blue.

**Extended Data Fig. 4:**
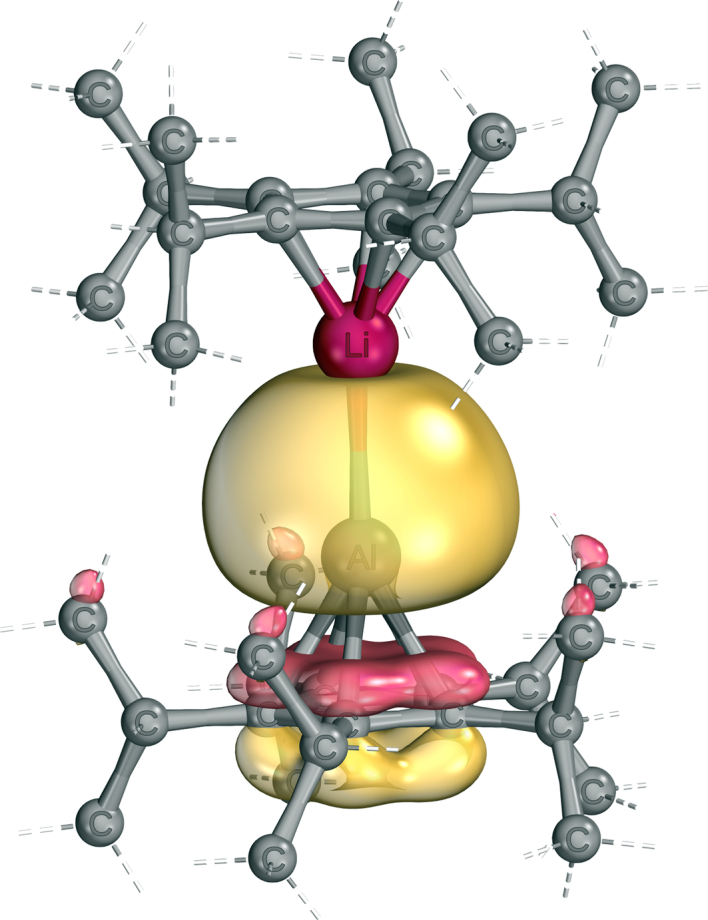
Intrinsic Bond Orbitals (IBO: M06-2X/def2-SVP) of **2**.

## Data Availability

Crystallographic data for the structures reported in this article have been deposited at the Cambridge Crystallographic Data Centre, under deposition numbers CCDC 2279422 (**1**), 2279423 (**1**∙**AlBr**_**3**_), 2279424 (**1**∙**W(CO)**_**5**_), 2324314°/°2279425 (**2**), 2324317 (**3**), 2324316 (**4a**), 2338161 (**4b**) and 2338185 (**5**). Copies of the data can be obtained free of charge via https://www.ccdc.cam.ac.uk/structures/. All other relevant data generated and analysed during this study, which include experimental, spectroscopic, crystallographic and computational data, are included in this article and its Supplementary Information. DFT coordinates of the optimized structures are provided as a supplementary data file. The authors declare that the data supporting the findings of this study are available within the paper or its Supplementary Information. Should any raw data files be needed in another format, they are available from the corresponding author upon reasonable request.

## References

[1] Kealy, T. J. & Pauson, P. L. A new type of organo-ion compound. *Nature* **168**, 1039–1040 (1951).

[2] Miller, S. A., Tebboth, J. A. & Tremaine, J. F. Dicyclopentadienyliron. *J. Chem. Soc*. 10.1039/JR9520000632 (1952).

[3] Adams, R. D. Foreword. *J. Organomet. Chem.* **637–639**, 1 (2001).

[4] Malischewski, M., Adelhardt, M., Sutter, J., Meyer, K. & Seppelt, K. Isolation and structural and electronic characterization of salts of the decamethylferrocene dication. *Science* **353**, 678–682 (2016). 27516596 10.1126/science.aaf6362

[5] Roy, G. et al. Ferrocene as an iconic redox marker: from solution chemistry to molecular electronic devices. *Coord. Chem. Rev.* **473**, 214816 (2022).

[6] Neuse, E. W. Macromolecular ferrocene compounds as cancer drug models. *J. Inorg. Organomet. Polym. Mater.* **15**, 3–31 (2005).

[7] Delferro, M. & Marks, T. J. Multinuclear olefin polymerization catalysts. *Chem. Rev.* **111**, 2450–2485 (2011). 21329366 10.1021/cr1003634

[8] Schäfer, A. Ferrocene and Related Metallocene Polymers. *Compr. Organomet. Chem. IV* **14**, 3–22 (2022).

[9] Štěpnička, P. Forever young: the first seventy years of ferrocene. *Dalton Trans.* **51**, 8085–8102 (2022). 35583080 10.1039/d2dt00903j

[10] Chirik, P. J. Group 4 transition metal sandwich complexes: still fresh after almost 60 years. *Organometallics* **29**, 1500–1517 (2010).

[11] Beswick, M. A., Palmer, J. S. & Wright, D. S. p-Block metallocenes—the other side of the coin. *Chem. Soc. Rev.* **27**, 225–232 (1998).

[12] Baguli, S., Mondal, S., Mandal, C., Goswami, S. & Mukherjee, D. Cyclopentadienyl complexes of the alkaline earths in light of the periodic trends. *Chem. Asian J.* **17**, e202100962 (2022). 34825506 10.1002/asia.202100962

[13] Schäfer, S., Kaufmann, S., Rösch, E. S. & Roesky, P. W. Divalent metallocenes of the lanthanides—a guideline to properties and reactivity. *Chem. Soc. Rev.* **52**, 4006–4045 (2023). 37183859 10.1039/d2cs00744d

[14] McClain, K. R. et al. Divalent lanthanide metallocene complexes with a linear coordination geometry and pronounced 6*s*–5*d* orbital mixing. *J. Am. Chem. Soc.* **144**, 22193–22201 (2022). 36417568 10.1021/jacs.2c09880

[15] Casado, C. M., Alonso, B. & García-Armada, M. P. Ferrocenes and other sandwich complexes of iron. *Compr. Organomet. Chem. IV* **7**, 3–45 (2022).

[16] Long, N. J. *Metallocenes—An Introduction to Sandwich Complexes* (Blackwell Scientific Publications, 1998).

[17] Resa, I., Carmona, E., Gutierrez-Puebla, E. & Monge, A. Decamethyldizincocene, a stable compound of Zn(I) with a Zn–Zn bond. *Science* **305**, 1136–1138 (2004). 15326350 10.1126/science.1101356

[18] Grirrane, A. et al. Zinc–zinc bonded zincocene structures. Synthesis and characterization of Zn_2_(η^5^-C_5_Me_5_)_2_ and Zn_2_(η^5^-C_5_Me_4_Et)_2_. *J. Am. Chem. Soc.* **129**, 693–703 (2007). 17227033 10.1021/ja0668217

[19] Schneider, J. J., Goddard, R., Werner, S. & Krüger, C. Reactivity of cobalt atoms towards 1,2,3,4,5-pentamethylcyclopentadienyl: synthesis and structure of bis(η^5^-pentamethylcyclopentadienyl)-(μ_2_-η^5^:η^5^-pentamethylcyclopentadienyl)dicobalt and bis(η^5^-pentamethylcyclopentadienyl)dicobalt. *Angew. Chem. Int. Ed. Engl.* **30**, 1124–1126 (1991).

[20] Kersten, J. L. et al. “[Cp*Co=CoCp*]” is a hydride. *Angew. Chem. Int. Ed. Engl.* **31**, 1341–1343 (1992).

[21] Schneider, J. J. On the reaction of pentamethylcyclopentadiene with cobalt atoms: a reexamination. *Angew. Chem. Int. Ed. Engl.* **31**, 1392 (1992).

[22] Gould, C. A. et al. Ultrahard magnetism from mixed-valence dilanthanide complexes with metal-metal bonding. *Science* **375**, 198–202 (2022). 35025637 10.1126/science.abl5470

[23] Lauk. S. & Schäfer, A. Pentaisopropyl cyclopentadienyl: an overview across the periodic table. *Eur. J. Inorg. Chem*. 10.1002/ejic.202100770 (2021).

[24] Xie, Y., Schaefer, H. F. III & Jemmis, E. D. Characteristics of novel sandwiched beryllium, magnesium, and calcium dimers: C_5_H_5_BeBeC_5_H_5_, C_5_H_5_MgMgC_5_H_5_, and C_5_H_5_CaCaC_5_H_5_. *Chem. Phys. Lett.* **402**, 414–421 (2005).

[25] Kan, Y. The nature of metal–metal bond of the dimetallocene complexes [M_2_(η^5^-C_5_R_5_)_2_] (M=Zn, Cd, Hg; R=H, Me): an energy decomposition analysis. *J. Mol. Struct. THEOCHEM* **805**, 127–132 (2007).

[26] Li, X. et al. Metal–metal and metal–ligand bonds in (η^5^-C_5_H_5_)_2_M_2_ (M=Be, Mg, Ca, Ni, Cu, Zn). *Organometallics* **32**, 1060–1066 (2013).

[27] Velazquez, A., Fernández, I., Frenking, G. & Merino, G. Multimetallocenes. A theoretical study. *Organometallics* **26**, 4731–4736 (2007).

[28] Wang, C.-Z. et al. Actinide (An=Th–Pu) dimetallocenes: promising candidates for metal–metal multiple bonds. *Dalton Trans.* **44**, 17045–17053 (2015). 26374594 10.1039/c5dt02811f

[29] Boronski, J. T., Crumpton, A. E., Wales, L. L. & Aldridge, S. Diberyllocene, a stable compound of Be(I) with a Be–Be bond. *Science* **380**, 1147–1149 (2023). 37319227 10.1126/science.adh4419

[30] Jutzi, P. The pentamethylcyclopentadienylsilicon(II) cation: synthesis, characterization, and reactivity. *Chem. Eur. J.* **20**, 9192–9207 (2014). 24986115 10.1002/chem.201402163

[31] Jutzi, P., Klipp, A., Mix, A., Neumann, B. & Stammler, H.-G. 1.2-Bis(pentamethylcyclopentadienyl)tetrachlorodisilane and its reduction to decamethylsilicocene. *Silicon Chem.* **3**, 151–156 (2007).

[32] Timoshkin, A. Y. & Schaefer, H. F. Donor–acceptor sandwiches of main-group elements. *Organometallics* **24**, 3343–3345 (2005).

[33] He, N., Xie, H.-b & Ding, Y.-h Can donor–acceptor bonded dinuclear metallocenes exist? A computational study on the stability of CpM′–MCp (M′=B, Al, Ga, In, Tl; M=Li, Na, K) and its isomers. *Organometallics* **26**, 6839–6843 (2007).

[34] Huo, S., Meng, D., Zhang, X., Meng, L. & Li, X. Bonding analysis of the donor–acceptor sandwiches CpE-MCp (E=B, Al, Ga; M=Li, Na, K; Cp=η^5^-C_5_H_5_). *J. Mol. Model.* **20**, 2455–2463 (2014). 25227450 10.1007/s00894-014-2455-6

[35] Dohmeier, C., Baum, E., Ecker, A., Köppe, R. & Schnöckel, H. Pentabenzylcyclopentadienides of lithium. *Organometallics* **15**, 4702–4706 (1996).

[36] Sitzmann, H., Lappert, M. F., Dohmeier, C., Üffing, C. & Schnöckel, H. Cyclopentadienylderivate von aluminium(I). *J. Organomet. Chem.* **561**, 203–208 (1998).

[37] Dohmeier, C., Robl, C., Tacke, M. & Schnöckel, H. The tetrameric aluminum(I) compound [{Al(η^5^-C_5_Me_5_)}_4_. *Angew. Chem. Int. Ed. Engl.* **30**, 564–565 (1991).

[38] Dabringhaus, P., Willrett, J. & Krossing, I. Synthesis of a low valent Al_4_^+^ cluster cation salt. *Nat. Chem.* **14**, 1151–1157 (2022). 35927330 10.1038/s41557-022-01000-4

[39] Hofmann, A., Tröster, T., Kupfer, T. & Braunschweig, H. Monomeric Cp^3t^Al(I): synthesis, reactivity, and the concept of valence isomerism. *Chem. Sci.* **10**, 3421–3428 (2019). 30996931 10.1039/c8sc05175e PMC6429597

[40] Hicks, J., Vasko, P., Goicoechea, J. M. & Aldridge, S. The aluminyl anion: a new generation of aluminium nucleophile. *Angew. Chem. Int. Ed.* **60**, 1702–1713 (2021). 10.1002/anie.202007530 32567755

[41] Cui, C. et al. Synthesis and structure of a monomeric aluminum(I) compound [{HC(CMeNAr)_2_}Al] (Ar=2,6-*i*Pr_2_C_6_H_3_): a stable aluminum analogue of a carbene. *Angew. Chem. Int. Ed.* **39**, 4274–4276 (2000). 10.1002/1521-3773(20001201)39:23<4274::AID-ANIE4274>3.0.CO;2-K 29711904

[42] Queen, J. D., Lehmann, A., Fettinger, J. C., Tuononen, H. M. & Power, P. P. The monomeric alanediyl:AlAr^*i*Pr8^ = C_6_H-2,6-(C_6_H_2_-2,4,6-Pr^*i*^_3_)2-3,5-Pr^*i*^_2_): an organoaluminum(I) compound with a one-coordinate aluminum atom. *J. Am. Chem. Soc*. **142**, 20554–20559 (2020). 10.1021/jacs.0c10222 33226797

[43] Li, X., Cheng, X., Song, H. & Cui, C. Synthesis of HC[(CBu^*t*^)(NAr)]_2_Al (Ar = 2,6-Pr^*i*^_2_C_6_H_3_) and its reaction with isocyanides, a bulky azide, and H_2_O. *Organometallics* **26**, 1039–1043 (2007).

[44] Zhang, X. & Liu, L. L. A free aluminylene with diverse σ-donating and doubly σ/π-accepting ligand features for transition metals. *Angew. Chem. Int. Ed.* **60**, 27062–27069 (2021). 10.1002/anie.202111975 34614275

[45] Zhang, X. & Liu, L. L. Reactivity of a free aluminylene towards Boron Lewis acids: accessing aluminum–boron-bonded species. *Eur. J. Inorg. Chem*. 10.1002/ejic.202300157 (2023).

[46] Hinz, A. & Müller, M. P. Attempted reduction of a carbazolyl-diiodoalane. *Chem. Commun.* **57**, 12532–12535 (2021). 10.1039/d1cc05557g 34751692

[47] Zhang, X., Mei, Y. & Liu, L. L. Free aluminylenes: an emerging class of compounds. *Chem. Eur. J.* **28**, e202202102 (2022). 35942883 10.1002/chem.202202102

[48] Dohmeier, C., Loos, D. & Schnöckel, H. Aluminum(I) and gallium(I) compounds: syntheses, structures, and reactions. *Angew. Chem. Int. Ed. Engl.* **35**, 129–149 (1996).

[49] Ahlrichs, R., Ehrig, M. & Horn, H. Bonding in the aluminum cage compounds [Al(η^5^-C_5_R_5_)]_4_ and Al_4_X_4_, X = H, F, Cl. *Chem. Phys. Lett*. **183**, 227–233 (1991).

[50] Gauss, J., Schneider, U., Ahlrichs, R., Dohmeier, C. & Schnöckel, H. ^27^Al NMR spectroscopic investigation of aluminum(I) compounds: ab initio calculations and experiment. *J. Am. Chem. Soc.* **115**, 2402–2408 (1993).

[51] Wade, K. The structural significance of the number of skeletal bonding electron-pairs in carboranes, the higher boranes and borane anions, and various transition-metal carbonyl cluster compounds. *J. Chem. Soc. D*10.1039/C29710000792 (1971).

[52] Mingos, D. M. P. A general theory for cluster and ring compounds of the main group and transition metals. *Nat. Phys. Sci.* **236**, 99–102 (1972).

[53] Welch, A. J. The significance and impact of Wade’s rules. *Chem. Commun.* **49**, 3615–3616 (2013). 10.1039/c3cc00069a 23535980

[54] Weiss, J. et al. [(η^5^-C_5_Me_5_)Al-Fe(CO)_4_] synthesis, structure, and bonding. *Angew. Chem. Int. Ed. Engl*. **36**, 70–72 (1997).

[55] Rayón, V. M. & Frenking, G. Structures, bond energies, heats of formation, and quantitative bonding analysis of main group metallocenes [E(Cp)_2_] (E=Be–Ba, Zn, Si–Pb) and [E(Cp)] (E=Li–Cs, B–Tl). *Chem. Eur. J.* **8**, 4693–4707 (2002). 12561110 10.1002/1521-3765(20021018)8:20<4693::AID-CHEM4693>3.0.CO;2-B

[56] Bader, R. F. W. A quantum theory of molecular structure and its applications. *Chem. Rev.* **91**, 893–928 (1991).

[57] Gonzáles-Gallardo, S., Bollermann, T., Fischer, R. A. & Murugavel, R. Cyclopentadiene based low-valent group 13 metal compounds: ligands in coordination chemistry and link between metal rich molecules and intermetallic materials. *Chem. Rev.* **112**, 3136–3170 (2012). 22364369 10.1021/cr2001146

[58] Hobson, K., Carmalt, C. J. & Bakewell, C. Recent advances in low oxidation state aluminium chemistry. *Chem. Sci.* **11**, 6942–6956 (2020). 34122993 10.1039/d0sc02686g PMC8159300

[59] Yu, Q., Purath, A., Donchev, A. & Schnöckel, H. The first structurally characterized coordination compound containing direct Al–Cr bonding: Cp*Al–Cr(CO)_5_. *J. Organomet. Chem.* **584**, 94–97 (1999).

[60] Fölsing, H. et al. Synthesis and structure of adduct stabilized Group III metal transition metal carbonyl complexes: new examples for Fe–Ga, Fe–In, W–Al, Cr–Al and Cr–Ga bonds. *J. Organomet. Chem.* **606**, 132–140 (2000).

[61] Roy, M. M. D. et al. Probing the extremes of covalency in M–Al bonds: lithium and zinc aluminyl compounds. *Angew. Chem. Int. Ed.* **60**, 22301–22306 (2021). 10.1002/anie.202109416 34396660

[62] Evans, M. J., Anker, M. D., McMullin, C. L., Neale, S. E. & Coles, M. P. Dihydrogen activation by lithium- and sodium-aluminyls. *Angew. Chem. Int. Ed.* **60**, 22289–22292 (2021). 10.1002/anie.202108934 34402149

[63] Pyykkö, P. Additive covalent radii for single-, double-, and triple-bonded molecules and tetrahedrally bonded crystals: a summary. *J. Phys. Chem. A* **119**, 2326–2337 (2015). 25162610 10.1021/jp5065819

[64] Zhao, L., von Hopffgarten, M., Andrada, D. M. & Frenking, G. Energy decomposition analysis. *WIREs Comput. Mol. Sci.* **8**, e13450 (2018).

[65] Schütz, M., Rauhut, G. & Werner, H. J. Local treatment of electron correlation in molecular clusters: structures and stabilities of (H_2_O)_*n*_, *n* = 2–4. *J. Phys. Chem. A* **102**, 5997–6003 (1998).

[66] Wuttke, A. & Mata, R. A. Visualizing dispersion interactions through the use of local orbital spaces. *J. Comp. Chem.* **38**, 15–23 (2017). 27761924 10.1002/jcc.24508

[67] del Río, D., Galindo, A., Resa, I. & Carmona, E. Theoretical and synthetic studies on [Zn_2_(η^5^-C_5_Me_5_)_2_]: analysis of the Zn–Zn bonding interaction. *Angew. Chem. Int. Ed.* **44**, 1244–1247 (2005). 10.1002/anie.200462175 15662655

[68] Jochmann, P. & Stephan, D. W. Zincocene and dizincocene N-heterocyclic carbene complexes and catalytic hydrogenation of imines and ketones. *Chem. Eur. J.* **20**, 8370–8378 (2014). 24861200 10.1002/chem.201402875

[69] Arduengo, A. J. III, Tamm, M., Calabrese, J. C., Davidson, F. & Marshall, W. J. Carbene–lithium interactions. *Chem. Lett.* **28**, 1021–1022 (1999).

[70] Wang, Y. et al. Labile imidazolium cyclopentadienides. *Organometallics* **38**, 4578–4584 (2019).

[71] Ledig, B., Marsch, M., Harms, K. & Boche, G. Lithiodiphenylmethylisocyanide-(−)-sparteine-bis(tetrahydrofuran): crystal structure of a lithiated isocyanide. *Angew. Chem. Int. Ed. Engl.* **31**, 79–80 (1992).

