# Supplementary material for: A lithium–aluminium heterobimetallic dimetallocene
Source: Nat Chem. 2024 May 14;16(7):1093–100. doi: 10.1038/s41557-024-01531-y (PMC11637000; doi:10.1038/s41557-024-01531-y)
Supplement: Supplementary file 1 — Supplementary Information. [file 41557_2024_1531_MOESM1_ESM.pdf]

nature chemistry

Article

https://doi.org/10.1038/s41557-024-01531-y

A lithium–aluminium heterobimetallic dimetallocene

In the format provided by the
authors and unedited

Experimental Procedures	S1	–	S5
NMR Spectra	S6	–	S18
UV-Vis Spectra			S19
IR Spectra	S20	–	S23
XRD Data	S24	–	S34
Computational Details	S35	–	S39
References	S40	–	S42

Experimental Procedures

General Information:

All manipulations were carried out under an argon inert gas atmosphere using Schlenk line techniques and/or a glove box. Solvents were purified using an MBraun Solvent Purification System. NMR spectra were recorded on Bruker Avance III 300 (solution), Bruker Avance III 400 (solution) and Bruker Avance III 400 WB (solid-state) spectrometers. ^{1}H and ^{13}C NMR spectra were referenced using the solvent signals,[1] ^{7}Li and ^{27}Al NMR spectra were referenced using external standards (δ^{27}Al($AlCl_3$ in D_2O) = 0; δ^{7}Li(LiCl in D_2O) = 0). Single crystal X-ray diffraction analyses were carried out on a Bruker X8 Apex II CCD diffractometer (**1·AlBr$_3$**, **1·W(CO)$_5$**, **3**, **4a**, **4b**, **5**) using monochromated $Mo_{K\alpha}$ radiation (λ = 0.71073 Å) and a Bruker D8 Venture diffractometer (**1**, **2**) using monochromated $Cu_{K\alpha}$ radiation (λ = 1.54178 Å). Data was corrected for absorption effects using the multi-scan method.[2] Elemental analyses were performed on an Elementar Vario MICRO cube. FT-IR spectra were recorded in attenuated total reflectance (ATR) on a Bruker Vertex 70 spectrometer. UV-Vis spectra were recorded in quartz glass cuvettes on a Perkin Elmer Lambda 750 spectrometer with an integrating sphere. {Cp*Al}$_4$[3] and ^{5}CpLi·OEt$_2$[4] were synthesized according to literature procedures.

Synthesis of 1 (^{5}CpAl):

To a mixture of ^{5}CpLi·OEt$_2$ (1.10 g, 3.08 mmol) and {Cp*Al}$_4$ (500 mg, 0.77 mmol) was added 1,2-difluorobenzene (~50 mL) at room temperature and the mixture was warmed to 343 K for 22 h. All volatiles were removed in vacuum and hexane (50 mL) was added. After filtration, all volatiles were removed in vacuum, and the resulting yellow to orange solid was purified by sublimation at 323 K / 0.01 mbar, giving **1** as a yellow to orange crystalline solid.

Yield: 97% / 225 mg / 0.75 mmol.

^{1}H NMR (400.13 MHz, C_6D_6, 293 K, δ in ppm): 1.25 (d, ^{3}J = 7.3 Hz, 15H, CH(C<u>H</u>$_3$)$_2$, 1.37 (d, ^{3}J = 7.3 Hz, 15H, CH(C<u>H</u>$_3$)$_2$), 3.09 (sept, ^{3}J = 7.3 Hz, 5H, C<u>H</u>(CH$_3$)$_2$).

^{13}C{^{1}H} NMR (100.62 MHz, C_6D_6, 293 K, δ in ppm): 22.5 (CH(<u>C</u>H$_3$)$_2$), 25.7 (<u>C</u>H(CH$_3$)$_2$), 26.0 (CH(<u>C</u>H$_3$)$_2$), 124.1 (<u>C</u>^{Cp}).

^{13}C{^{1}H} NMR (100.67 MHz, CP/MAS(13 kHz), 294 K, δ in ppm): 22-27 (<u>C</u>H(CH$_3$)$_2$ & CH(<u>C</u>H$_3$)$_2$), 124 (<u>C</u>^{Cp}).

^{27}Al{^{1}H} NMR (104.26 MHz, C_6D_6, 293 K, δ in ppm): -154 ($\omega_{1/2}$ = 521 Hz).

^{27}Al NMR (104.36 MHz, SPE/MAS(13 kHz), 295 K, δ_{iso} in ppm): -154 (δ_{iso} was determined by simulation of the spectrum with the Bruker TopSpin 3.6.4 software suite).

CHN-analysis: found: C: 79.43%, H: 11.63%; calc. for $C_{20}H_{35}Al$: C: 79.42%, H: 11.66%.

UV-Vis [nm]: 202, 218, 256.

IR (ν_{max} / cm^{-1}): 521 (s), 910 (w), 1091 (w), 1113 (w), 1161 (w), 1316 (w), 1365 (m), 1381 (w), 1456 (w), 2868 (w), 2926 (w), 2952 (m), 2976 (m).

Synthesis of 1·$AlBr_3$ (^{5}CpAl→$AlBr_3$):

1 (150 mg, 0.50 mmol) and $AlBr_3$ (132 mg, 0.50 mmol) were mixed and stirred in toluene (~40 mL) for 15 min. The solvent was removed from the precipitated colorless solid *via* syringe, a 4:1 mixture of 1,2-difluorobenzene (~4 mL) and toluene (~1 mL) was added, and the mixture was heated to just below the boiling temperature. Slow cooling to room temperature afforded colorless crystals of **1·$AlBr_3$**.

Yield: 72 % / 205 mg / 0.36 mmol.

^{1}H NMR (400.13 MHz, C_6D_6, 292 K, δ in ppm): 0.88 (d, ^{3}J = 7.3 Hz, 15H, CH(C$\underline{H}_3$)$_2$), 1.30 (d, ^{3}J = 7.4 Hz, 15H, CH(C$\underline{H}_3$)$_2$), 2.97 (sept, ^{3}J = 7.3 Hz, 5H, C$\underline{H}$(CH_3)$_2$).

^{13}C{^{1}H} NMR (100.62 MHz, C_6D_6, 292 K, δ in ppm): 20.6 (CH($\underline{C}H_3$)$_2$), 25.7 ($\underline{C}$H(CH_3)$_2$), 26.1 (CH($\underline{C}H_3$)$_2$), 126.8 ($\underline{C}^{Cp}$).

No signal was detected in the ^{27}Al{^{1}H} NMR spectrum in a range between -300 to +300 ppm.

CHN-analysis: found: C: 42.32%, H: 6.09%; calc. for $C_{20}H_{35}Al_2Br_2$: C: 42.20%, H: 6.20%.

IR (ν_{max} / cm^{-1}): 594 (s), 648 (w), 693 (w), 719 (w), 784 (w), 884 (w), 1086 (m), 1114 (w), 1162 (w), 1314 (w), 1334 (w), 1355 (m), 1386 (w), 1433 (m), 2875 (w), 2924 (w), 2943 (m), 2986 (m).

Synthesis of 1·W(CO)$_5$ (^{5}CpAl→W(CO)$_5$):

A mixture of **1** (125 mg, 0.41 mmol) and W(CO)$_6$ (145 mg, 0.41 mmol) in THF (~3 mL) was irradiated at 365 nm for 15 min. All volatiles were removed in vacuum, and toluene was added. Slow evaporation of toluene afforded colorless crystals of **1·W(CO)$_5$**, which were washed twice with small amounts of hexane and subsequently dried in vacuum.

(Note: A minor side-product was detected by NMR, which could not be separated completely and is believed to be the bis(aluminylene) tungsten tetracarbonyl complex (^{5}CpAl→)$_2$W(CO)$_4$.

Yield: 9% / 22 mg / 0.04 mmol.

^{1}H NMR (400.13 MHz, C_6D_6, 293 K, δ in ppm): 1.02 (d, ^{3}J = 7.3 Hz, 15H, CH(C$\underline{H}_3$)$_2$), 1.35 (d, ^{3}J = 7.3 Hz, 15H, CH(C$\underline{H}_3$)$_2$), 3.08 (sept, ^{3}J = 7.3 Hz, 5H, C$\underline{H}$(CH_3)$_2$).

^{13}C{^{1}H} NMR (100.62 MHz, C_6D_6, 293 K, δ in ppm): 21.8 (CH($\underline{C}H_3$)$_2$), 24.3 ($\underline{C}$H(CH_3)$_2$), 26.1 (CH($\underline{C}H_3$)$_2$), 125.9 ($\underline{C}^{Cp}$), 199.0 (W($\underline{C}$O)$_5$), 199.2 (W($\underline{C}$O)$_5$).

No signal was detected in the ^{27}Al{^{1}H} NMR spectrum in a range between -300 to +300 ppm.

(Minor side-product: ^{1}H NMR (400.13 MHz, C_6D_6, 293 K, δ in ppm): 1.14 (d, ^{3}J = 7.3 Hz, 15H, CH(C$\underline{H}_3$)$_2$), 1.64 (d, ^{3}J = 7.3 Hz, 15H, CH(C$\underline{H}_3$)$_2$), 3.30 (sept, ^{3}J = 7.3 Hz, 5H, C$\underline{H}$(CH_3)$_2$). ^{13}C{^{1}H} NMR (100.62 MHz, C_6D_6, 293 K, δ in ppm): 22.2 (CH($\underline{C}H_3$)$_2$), 24.2 ($\underline{C}$H(CH_3)$_2$), 26.5 (CH($\underline{C}H_3$)$_2$), 125.6 (C^{Cp}), 199.2 (W($\underline{C}$O)$_5$)).)

CHN-analysis: Found: C: 46.50%; H: 6.02%. Calc. for $C_{25}H_{35}AlO_5W$: C: 47.94%; H: 5.63%.

IR (ν_{max} / cm^{-1}): 571 (s), 598 (w), 798 (m), 1018 (m), 1086 (m), 1259 (w), 1371 (w), 1458 (w), 1905 (s, CO), 1965 (w, CO), 2052 (w, CO), 2361 (w), 2875 (w), 2935 (w), 2962 (w).

Synthesis of 2 (^{5}CpAl→Li^{5}Cp):

METHOD A:

To a mixture of ^{5}CpLi•OEt$_2$ (813 mg, 2.28 mmol) and {Cp*Al}$_4$ (185 mg, 0.29 mmol) was added 1,2-difluorobenzene (~40 mL) at room temperature and the mixture was warmed to 343 K for 22 h. All volatiles were removed in vacuum and toluene (~40 mL) was added. After filtration, all volatiles were removed in vacuum, and precipitated **2** was washed with hexane (~3 mL). Colorless crystals of **2** could be obtained from a 1:1 mixture of toluene (3 mL) and 1,2-difluorobenzene (3 mL) at 249 K.

Yield: 33% / 440 mg / 0.75 mmol.

METHOD B:

To a mixture of ^{5}CpLi•OEt$_2$ (59 mg, 0.17 mmol), **1** (50 mg, 0.17 mmol) and Cp*Li (24 mg, 0.17 mmol) was added 1,2-difluorobenzene (~25 mL) at room temperature. The mixture was stirred for 1 h at room temperature and subsequently heated at 323 K for 18 h. After cooling to room temperature and filtration all volatiles were removed in vacuum to give **2**.

Yield: 93% / 92 mg / 0.16 mmol.

^{1}H NMR (400.13 MHz, C_6D_6, 293 K, δ in ppm): 1.23 (d, ^{3}J = 7.2 Hz, 15H, CH(C$\underline{H}_3$)$_2$:^{5}CpAl), 1.29 (d, ^{3}J = 7.3 Hz, 15H, CH(C$\underline{H}_3$)$_2$:^{5}CpLi), 1.35 (d, ^{3}J = 7.4 Hz, 15H, CH(C$\underline{H}_3$)$_2$:^{5}CpAl), 1.50 (d, ^{3}J = 7.3 Hz, 15H, CH(C$\underline{H}_3$)$_2$:^{5}CpLi), 3.07 (sept, ^{3}J = 7.4 Hz, 5H, C$\underline{H}$(CH$_3$)$_2$:^{5}CpAl), 3.32 (sept, ^{3}J = 7.2 Hz, 5H, C$\underline{H}$(CH$_3$)$_2$:^{5}CpLi).

^{13}C{^{1}H} NMR (100.62 MHz, C_6D_6, 293 K, δ in ppm): 22.4 (CH($\underline{C}$H$_3$)$_2$:^{5}CpAl), 23.9 (CH($\underline{C}$H$_3$)$_2$:^{5}CpLi), 25.6 ($\underline{C}$H(CH$_3$)$_2$:^{5}CpAl), 25.9 ($\underline{C}$H(CH$_3$)$_2$:^{5}CpLi), 26.0 (CH($\underline{C}$H$_3$)$_2$:^{5}CpAl), 27.0 (CH($\underline{C}$H$_3$)$_2$:^{5}CpLi), 118.1 (q, $^{1}J_{^{13}C-^{7}Li}$ = 2.2 Hz, $\underline{C}^{Cp}$:^{5}CpLi), 124.1 ($\underline{C}^{Cp}$:^{5}CpAl).

^{13}C{^{1}H} NMR (100.67 MHz, CP/MAS(13 kHz), 295 K, δ_{iso} in ppm): 22-26 ($\underline{C}$H(CH$_3$)$_2$ & CH($\underline{C}$H$_3$)$_2$), 118 ($\underline{C}^{Cp}$:^{5}CpLi), 124 ($\underline{C}^{Cp}$:^{5}CpAl).

^{7}Li NMR (155.51 MHz, C_6D_6, 293 K, δ in ppm): -9.63.

^{7}Li{^{1}H} NMR (155.57 MHz, SPE/MAS(13 kHz), 298 K, δ_{iso} in ppm): -8.9 (hex, $^{1}J^{\circ}{}_{^{7}Li-^{27}Al}$ = 102 Hz) (δ_{iso} and the $^{1}J_{^{7}Li-^{27}Al}$ coupling constant were determined by simulation of the spectrum with the Bruker TopSpin 3.6.4 software suite).

^{27}Al{^{1}H} NMR (104.26 MHz, C_6D_6, 293 K, δ in ppm): -151 ($\omega_{1/2}$ = 1139 Hz).

^{27}Al NMR (104.36 MHz, SPE/MAS(13 kHz), 297 K, δ_{iso} in ppm): -157 (δ_{iso} was determined by simulation of the spectrum with the Bruker TopSpin 3.6.4 software suite).

CHN-analysis: found: C: 81.61%, H: 11.70%; calc. for $C_{40}H_{70}AlLi$: C: 82.14%, H: 12.06%.

IR (ν_{max} / cm^{-1}): 540 (s), 604 (w), 804 (w), 908 (w), 1020 (w), 1084 (m), 1109 (m), 1159 (m), 1261 (w), 1313 (w), 1362 (m), 1454 (m), 2866 (m), 2935 (s), 2958 (s).

Synthesis of 3 (^{5}CpLi·NHC):

A mixture of **2** (70 mg, 0.12 mmol) and 1,3-diisopropyl-4,5-dimethylimidazol-2-ylidene (22 mg, 0.12 mmol) in toluene (~4 mL) was stirred for 2 h at room temperature, and subsequently stored at 249 K, to obtain **3** in form of colorless crystals.

Yield: 49% / 27 mg / 0.06 mmol.

^{1}H NMR (300.13 MHz, C_6D_6, 298 K, δ in ppm): 1.10 (d, ^{3}J = 6.9 Hz, 12H, CH(C$\underline{H}_3$)$_2$:NHC), 1.56 (d, ^{3}J = 7.3 Hz, 15H, CH(C$\underline{H}_3$)$_2$:^{5}CpLi), 1.61 (s, 6H, C$\underline{H}_3$:NHC), 1.68 (d, ^{3}J = 7.3 Hz, 15H, CH(C$\underline{H}_3$)$_2$:^{5}CpLi), 3.60 (sept, ^{3}J = 7.3 Hz, 5H, C$\underline{H}$(CH$_3$)$_2$:^{5}CpLi), 4.65 (sept, ^{3}J = 7.2 Hz, 2H, C$\underline{H}$(CH$_3$)$_2$:NHC).

^{13}C{^{1}H} NMR (100.62 MHz, C_6D_6, 293 K, δ in ppm): 10.0 ($\underline{C}$H$_3$:NHC), 22.4 (CH($\underline{C}$H$_3$)$_2$:^{5}CpLi), 24.6 (CH($\underline{C}$H$_3$)$_2$:^{5}CpLi), 26.7 ($\underline{C}$H(CH$_3$)$_2$:^{5}CpLi), 27.4 (CH($\underline{C}$H$_3$)$_2$:NHC), 53.7 ($\underline{C}$H(CH$_3$)$_2$:NHC), 118.4 ($\underline{C}^{Cp}$), 124.5 ($\underline{C}$=$\underline{C}$:NHC), 193.8 ($\underline{C}^{Carbene}$).

^{7}Li NMR (155.51 MHz, C_6D_6, 293 K, δ in ppm): -7.41.

CHN-analysis: found: C: 78.68%, H: 11.77%, N 5.84%; calc. for $C_{31}H_{55}LiN_2$: C: 80.47%, H: 11.98%, N 6.05% (Carbon values were repeatedly, reproducibly low).

IR (ν_{max} / cm^{-1}): 546 (w), 742 (w), 796 (w), 908 (w), 1066 (m), 1080 (m), 1105 (m), 1134 (m), 1157 (m), 1225 (w), 1309 (w), 1354 (m), 1371 (m), 1402 (w), 1448 (m), 1556 (w), 1579 (w), 1647 (w), 2860 (s), 2922 (s), 2966 (s).

Synthesis of 4a (^{5}CpLi·CNPh):

To a solution of **2** (60 mg, 0.10 mmol) in toluene (~10 mL) was added phenylisocyanate (12 mg, 0.10 mmol, 0.01 mL) at room temperature and the reaction mixture was subsequently stirred for 10 min. All volatiles were removed in vacuum and the residue was dissolved in a mixture of toluene (~5 mL) and hexane (~2 mL). **4a** was obtained in form of highly air sensitive colorless crystals by storing the solution at 249 K.

Yield: 29% / 11 mg / 0.03 mmol.

^{1}H NMR (400.13 MHz, C_6D_6, 293 K, δ in ppm): 1.48 (d, ^{3}J = 7.2 Hz, 15H, CH(C$\underline{H}_3$)$_2$), 1.60 (d, ^{3}J = 7.2 Hz, 15H, CH(C$\underline{H}_3$)$_2$), 3.49 (sept, ^{3}J = 7.3 Hz, 5H, C$\underline{H}$(CH$_3$)$_2$), 6.54-6.56 (m, 4H, $\underline{H}^{Ph}$), 6.65-6.69 (m, 1H, $\underline{H}^{Ph}$).

^{13}C{^{1}H} NMR (100.62 MHz, C_6D_6, 299 K, δ in ppm): 23.8 (CH($\underline{C}$H$_3$)$_2$), 26.7 (CH($\underline{C}$H$_3$)$_2$), 27.2 ($\underline{C}$H(CH$_3$)$_2$), 118.6 (q, $^{1}J_{^{13}C-^{7}Li}$ = 2.2 Hz, $\underline{C}^{Cp}$), 126.4 ($\underline{C}^{Ph}$), 128.7 ($\underline{C}^{Ph}$), 129.3 ($\underline{C}^{Ph}$), 130.0 ($\underline{C}^{Ph}$).

^{7}Li NMR (155.51 MHz, C_6D_6, 293 K, δ in ppm): -9.07.

IR (ν_{max} / cm^{-1}): 484 (w), 511 (w), 619 (w), 685 (m), 760 (s), 796 (s), 1016 (s), 1080 (s), 1155 (w), 1259 (s), 1362 (w), 1454 (w), 1487 (w), 1589 (w), 2123 (w), 2181 (w), 2868 (w), 2939 (m), 2964 (s).

No satisfactory elemental analysis could be obtained, due to the extreme air sensitivity of this compound.

Synthesis of 4b (^{5}CpLi·CNMes):

To a solution of **2** (50 mg, 0.09 mmol) in hexane (~10 mL) was added mesitylisothiocyanate (15 mg, 0.09 mmol) at room temperature and the reaction mixture was subsequently stirred for 10 min. **4b** was obtained in form of highly air sensitive colorless crystals by storing the solution at 249 K.

Yield: 78% / 30 mg / 0.07 mmol.

^{1}H NMR (300.13 MHz, C_6D_6, 297 K, δ in ppm): 1.52 (d, ^3J = 7.3 Hz, 15H, CH(C<u>H</u>$_3$)$_2$), 1.62 (d, ^3J = 7.3 Hz, 15H, CH(C<u>H</u>$_3$)$_2$), 1.83 (s, 3H, *p*-CH$_3^{Mes}$), 1.89 (s, 6H, *o*-CH$_3^{Mes}$), 3.52 (sept, ^3J = 7.3 Hz, 5H, C<u>H</u>(CH$_3$)$_2$), 6.29 (s, 2H, *m*-<u>H</u>^{Mes}).

^{13}C{^{1}H} NMR (75.48 MHz, C_6D_6, 297 K, δ in ppm): 18.2 (<u>C</u>H$_3^{Mes}$), 20.9 (<u>C</u>H$_3^{Mes}$), 23.8 (CH(<u>C</u>H$_3$)$_2$), 26.8 (<u>C</u>H(CH$_3$)$_2$), 27.2 (CH(<u>C</u>H$_3$)$_2$), 118.5 (q, $^1J_{^{13}C-^7Li}$ = 2.2 Hz, <u>C</u>^{Cp}), 128.7 (<u>C</u>^{Ar}), 135.1 (<u>C</u>^{Ar}).

^{7}Li NMR (116.64 MHz, C_6D_6, 297 K, δ in ppm): -8.92.

IR (ν_{max} / cm^{-1}): 474 (m), 503 (m), 599 (w), 637 (m), 673 (w), 741 (m), 816 (m), 854 (m), 906 (w), 1058 (s), 1100 (s), 1156 (m), 1365 (m), 1381 (m), 1458 (m), 1608 (w), 2113 (m), 2870 (m), 2929 (s), 2961 (s).

No satisfactory elemental analysis could be obtained, due to the extreme air sensitivity of this compound.

Synthesis of 5 ({^{5}CpAlNAd}$_2$):

To a solution of **2** (30 mg, 0.05 mmol) in toluene (~2 mL) precooled to 233 K was added 1-azidoadamantan (9 mg, 0.05 mmol) in toluene (~2 mL) at 233 K and the reaction mixture was subsequently stirred for 10 min and allowed to warm to room temperature. NMR spectroscopy indicated the reaction solution to be a mixture of **5** and ^{5}CpLi, which could not be separated. On one occasion, serendipitous crystals of **5** were obtained by storing a toluene solution at 249 K.

^{1}H NMR (400.13 MHz, toluene-D$_8$, 297 K, δ in ppm): 1.26 (d, ^3J = 7.3 Hz, CH(C<u>H</u>$_3$)$_2$:^{5}CpLi), 1.41 (d, ^3J = 7.3 Hz, CH(C<u>H</u>$_3$)$_2$:^{5}CpLi), 1.42 (d, ^3J = 7.2 Hz, CH(C<u>H</u>$_3$)$_2$:^{5}CpAl), 1.61-1.91 (m, Ad), 3.26 (sept, ^3J = 7.3 Hz, C<u>H</u>(CH$_3$)$_2$:^{5}CpLi), 3.34 (sept, ^3J = 7.3 Hz, C<u>H</u>(CH$_3$)$_2$:^{5}CpAl).

^{13}C{^{1}H} NMR (100.13 MHz, toluene-D$_8$, 297 K, δ in ppm): 24.3 (CH(<u>C</u>H$_3$)$_2$:^{5}CpAl), 24.9 (CH(<u>C</u>H$_3$)$_2$:^{5}CpLi), 26.2 (CH(<u>C</u>H$_3$)$_2$:^{5}CpLi), 27.2 (<u>C</u>H(CH$_3$)$_2$:^{5}CpAl), 27.4 (<u>C</u>H(CH$_3$)$_2$:^{5}CpLi), 30.8 (Ad), 31.6 (Ad), 37.3 (Ad), 37.4 (Ad), 44.5 (Ad), 50.7 (Ad), 53.8 (Ad), 118.4 (<u>C</u>^{Cp}:^{5}CpLi), 126.8 (<u>C</u>^{Cp}:^{5}CpAl).

^{7}Li NMR (155.51 MHz, toluene-D$_8$, 297 K, δ in ppm): -9.54.

No signal was detected in the ^{27}Al{^{1}H} NMR spectrum in a range between -300 to +300 ppm.

NMR Spectra

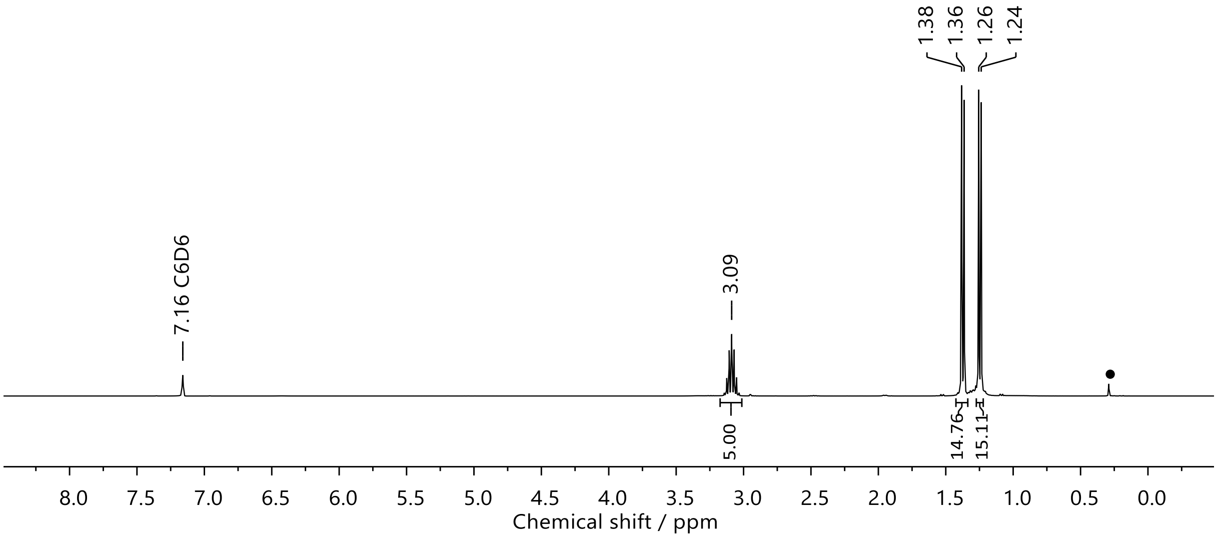

Supplementary Figure 1: ^{1}H NMR spectrum (400.13 MHz, C_6D_6, 293 K) of **1** (^{5}CpAl) (● silicon grease).

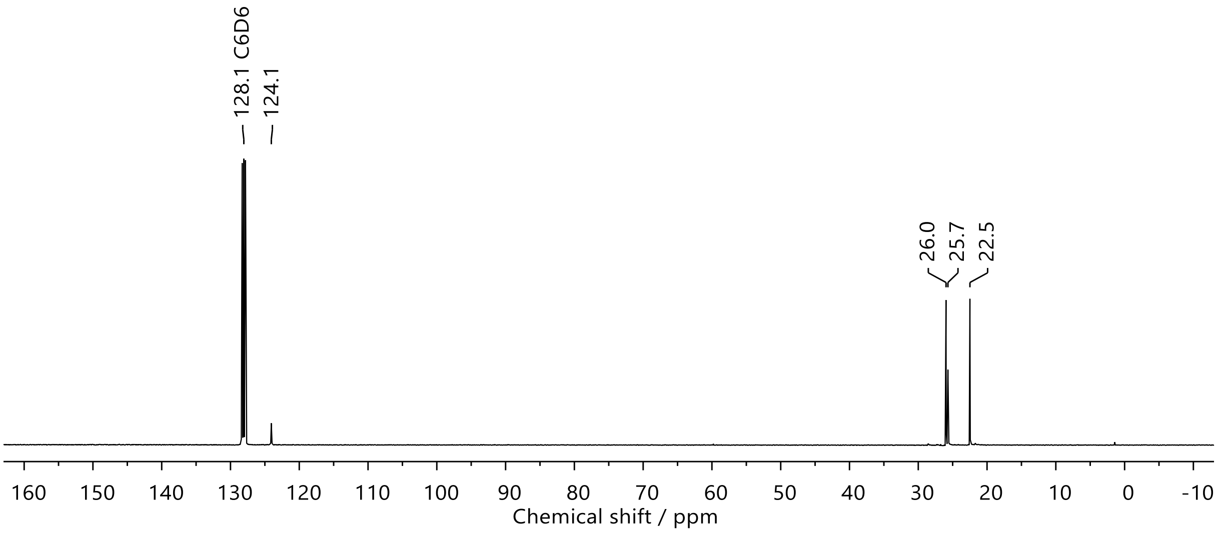

Supplementary Figure 2: $^{13}C\{^{1}H\}$ NMR spectrum (100.62 MHz, C_6D_6, 293 K) of **1** (^{5}CpAl).

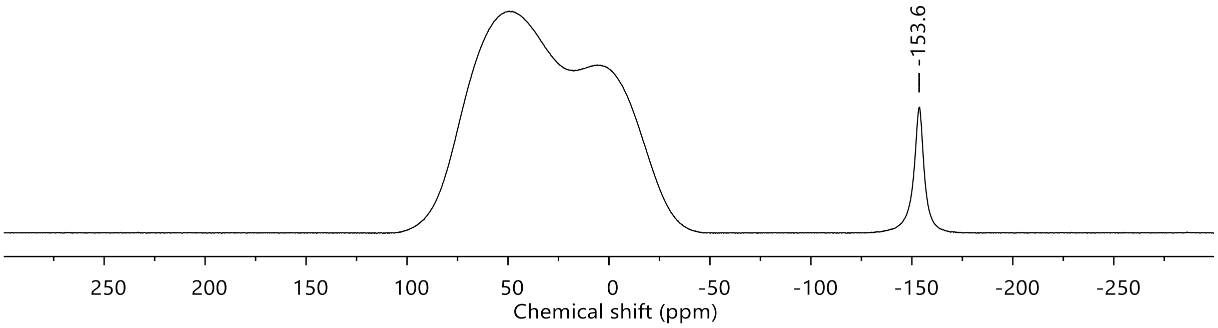

Supplementary Figure 3: $^{27}Al\{^1H\}$ NMR spectrum (104.26 MHz, C_6D_6, 293 K) of **1** (^5CpAl).

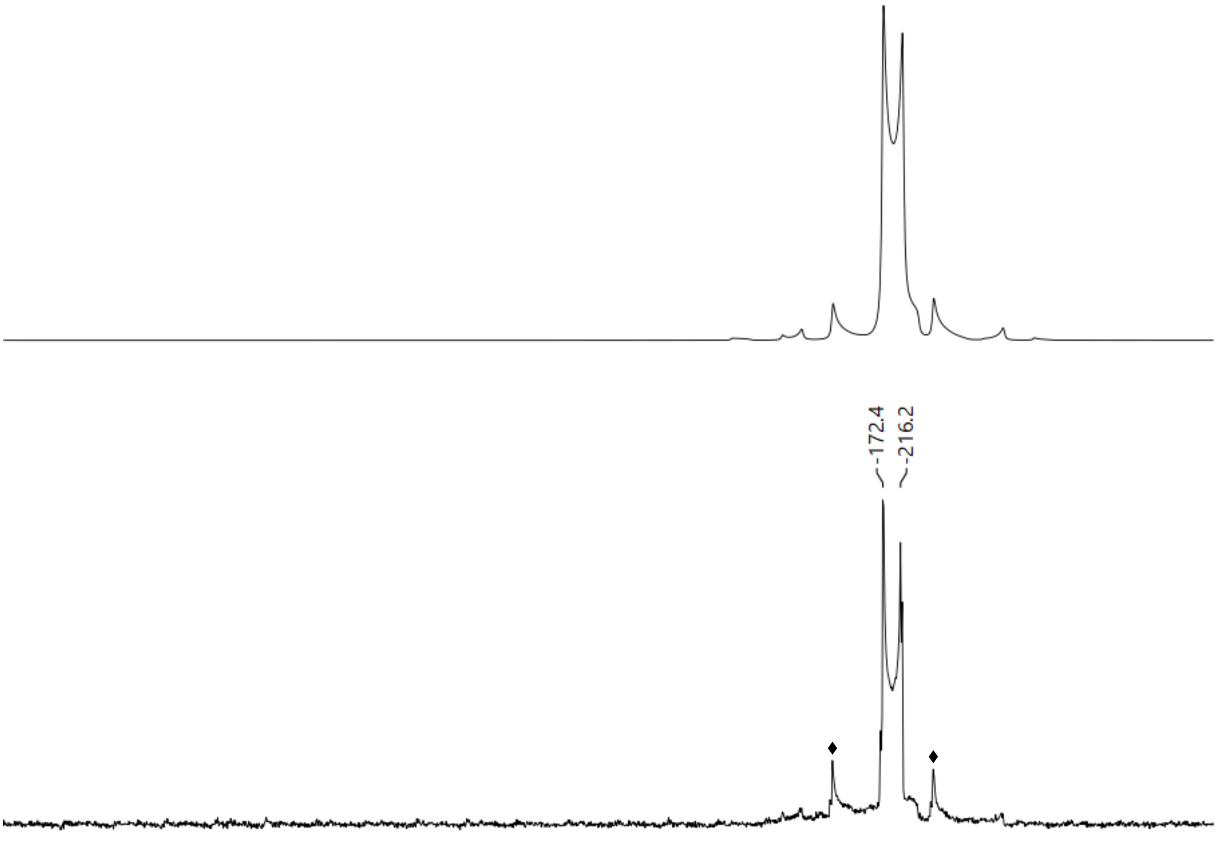

Supplementary Figure 4: ^{27}Al SPE/MAS(13kHz) NMR spectrum (104.36 MHz, 295 K) of **1** (^5CpAl) (top: simulated spectrum; bottom: experimental spectrum; analysis and simulations were performed with the Bruker TopSpin 3.6.4 software suite; ♦ spinning sidebands).

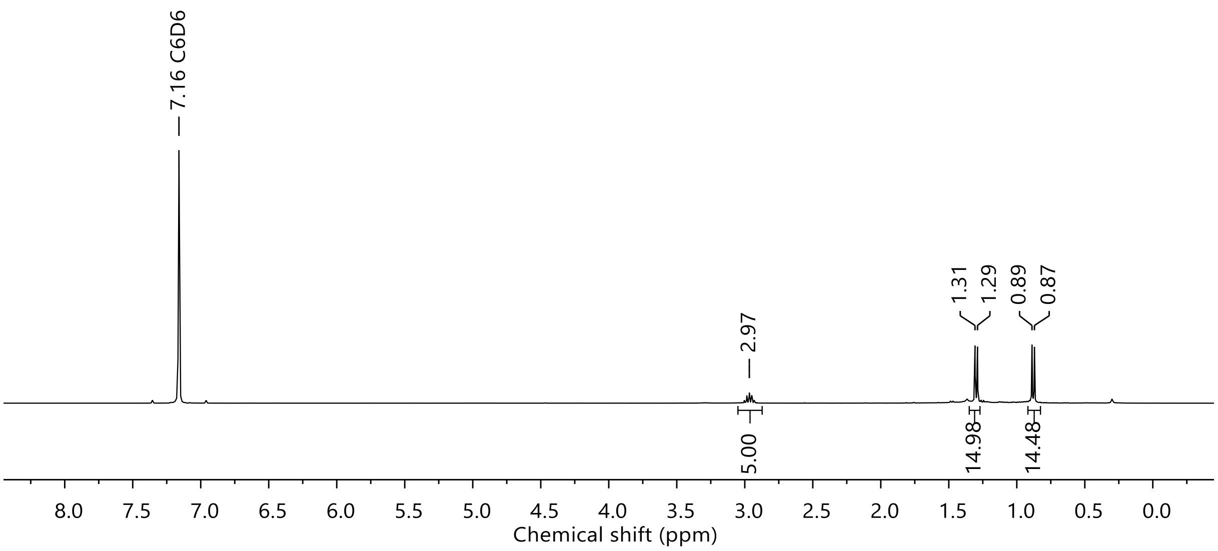

Supplementary Figure 5: ^{1}H NMR spectrum (400.13 MHz, C_6D_6, 292 K) of **1·AlBr$_3$** (^{5}CpAl→$AlBr_3$).

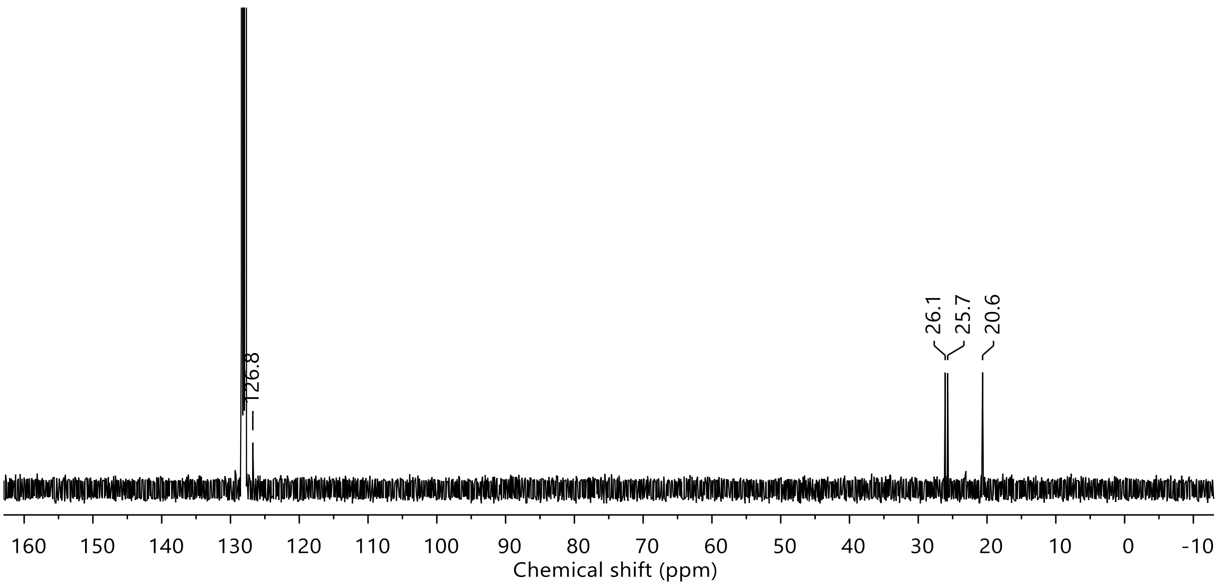

Supplementary Figure 6: $^{13}C\{^{1}H\}$ NMR spectrum (100.62 MHz, C_6D_6, 292 K) of **1·AlBr$_3$** (^{5}CpAl→$AlBr_3$).

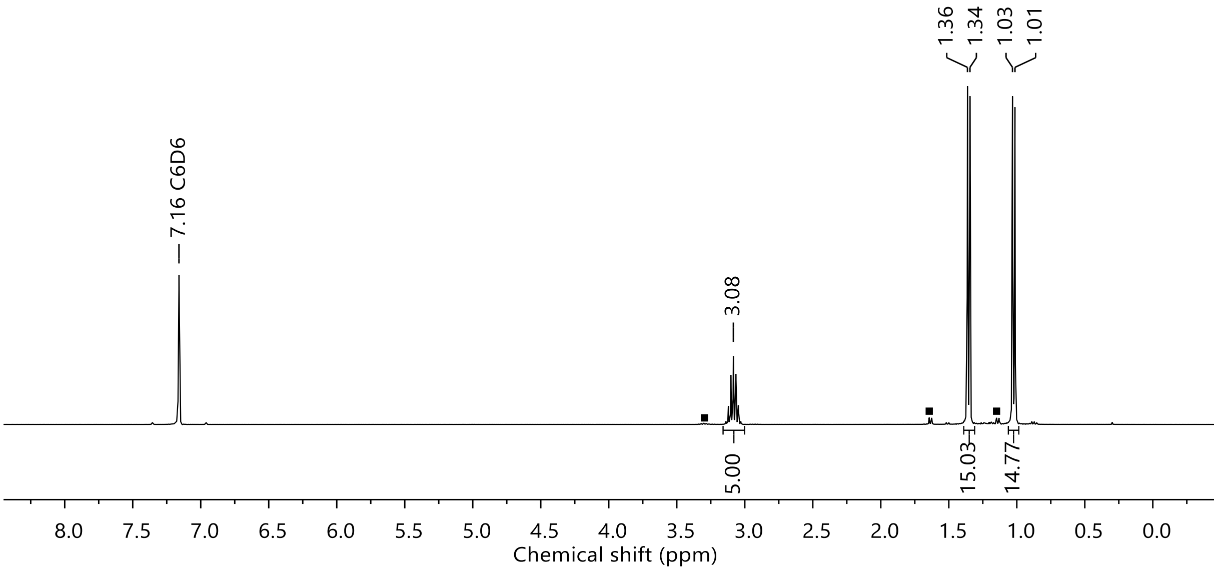

Supplementary Figure 7: ^{1}H NMR spectrum (400.13 MHz, C_6D_6, 293 K) of **1·W(CO)₅** (^{5}CpAl→W(CO)$_5$) (■ side product).

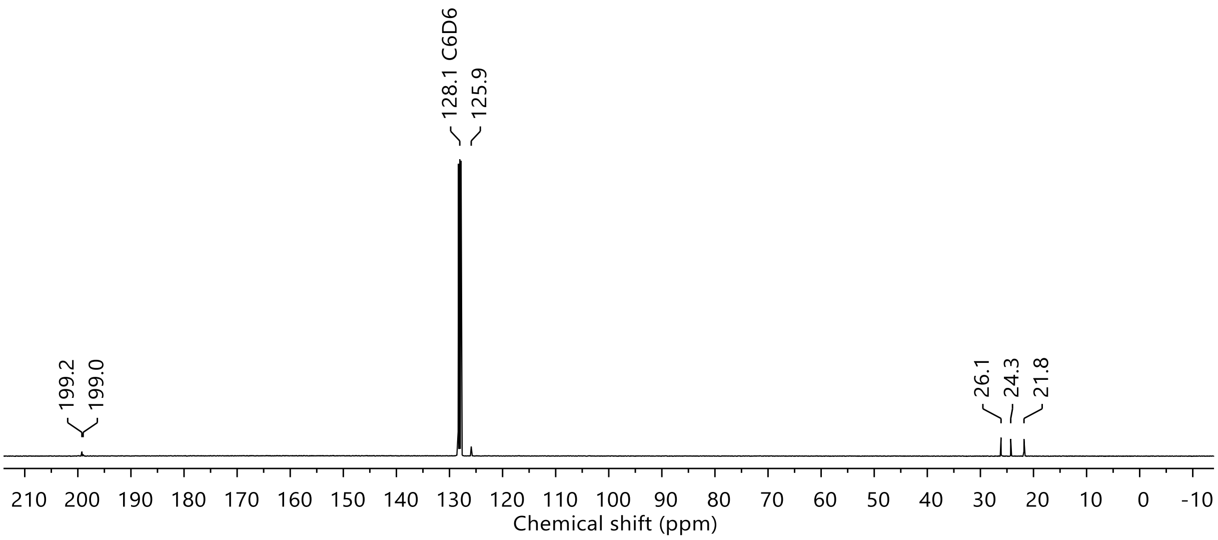

Supplementary Figure 8: $^{13}C\{^{1}H\}$ NMR spectrum (100.62 MHz, C_6D_6, 293 K) of **1·W(CO)$_5$** (^{5}CpAl→W(CO)$_5$).

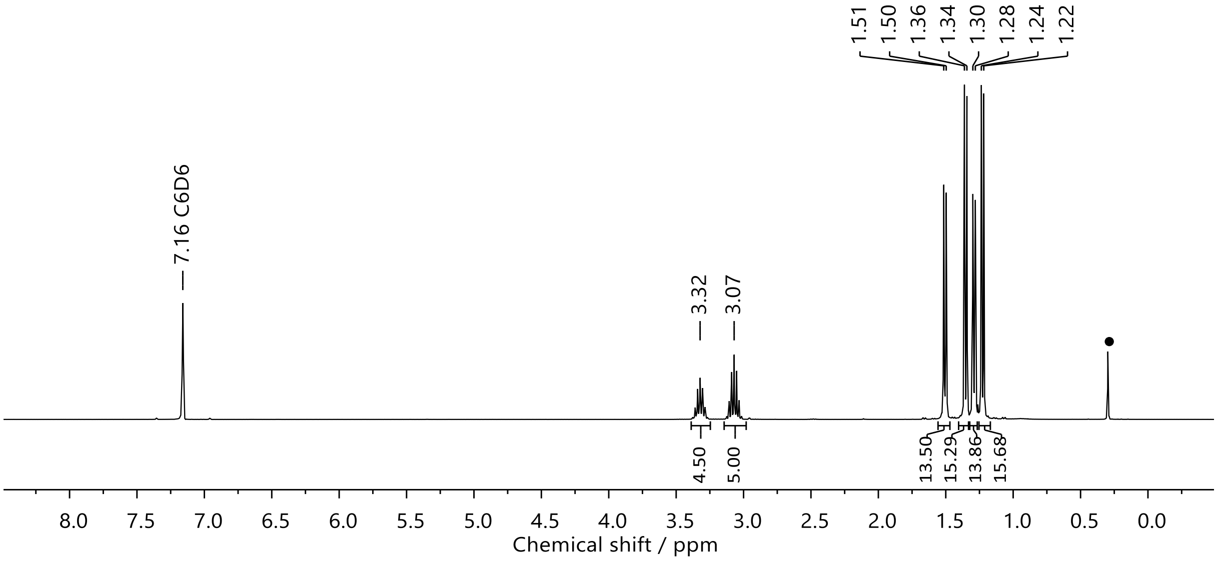

Supplementary Figure 9: ^{1}H NMR spectrum (400.13 MHz, C_6D_6, 293 K) of **2** ($^{5}CpAl\rightarrow Li^{5}Cp$) (● silicon grease).

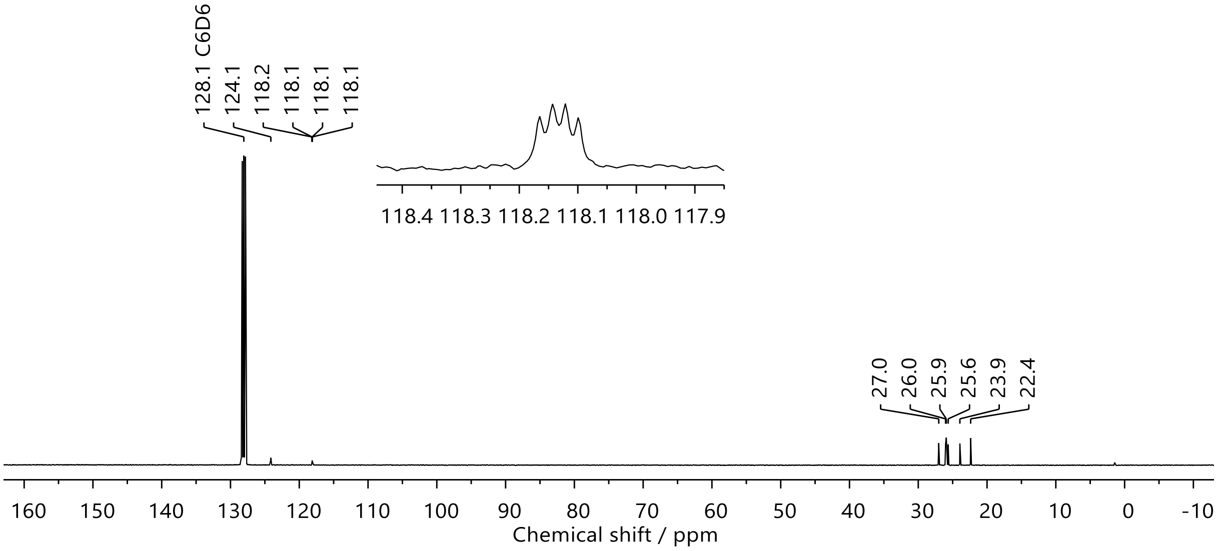

Supplementary Figure 10: $^{13}C\{^{1}H\}$ NMR spectrum (100.62 MHz, C_6D_6, 293 K) of **2** ($^{5}CpAl\rightarrow Li^{5}Cp$).

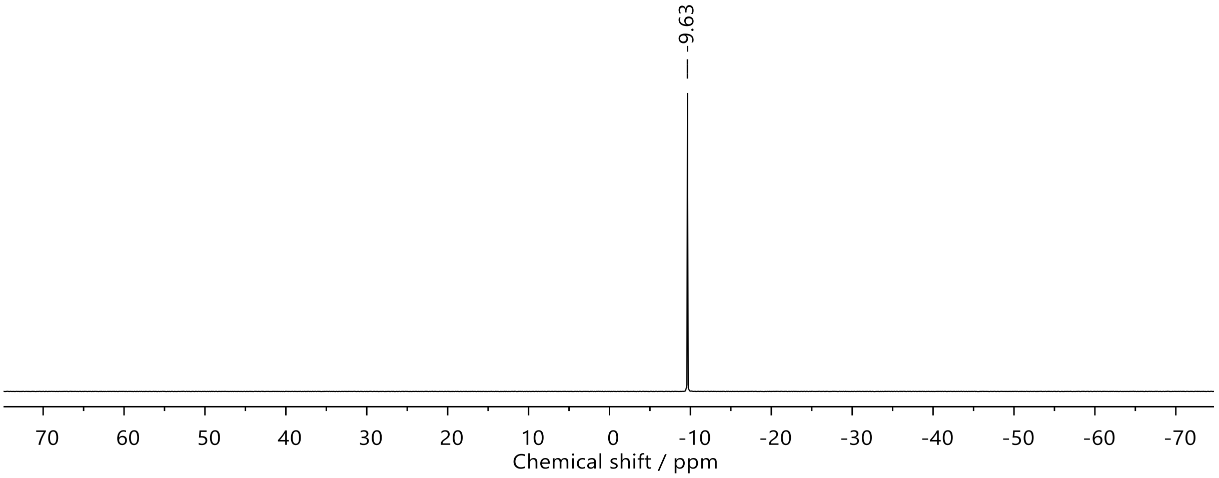

Supplementary Figure 11: ^{7}Li NMR spectrum (155.51 MHz, C_6D_6, 293 K) of **2** ($^{5}CpAl{\rightarrow}Li^{5}Cp$).

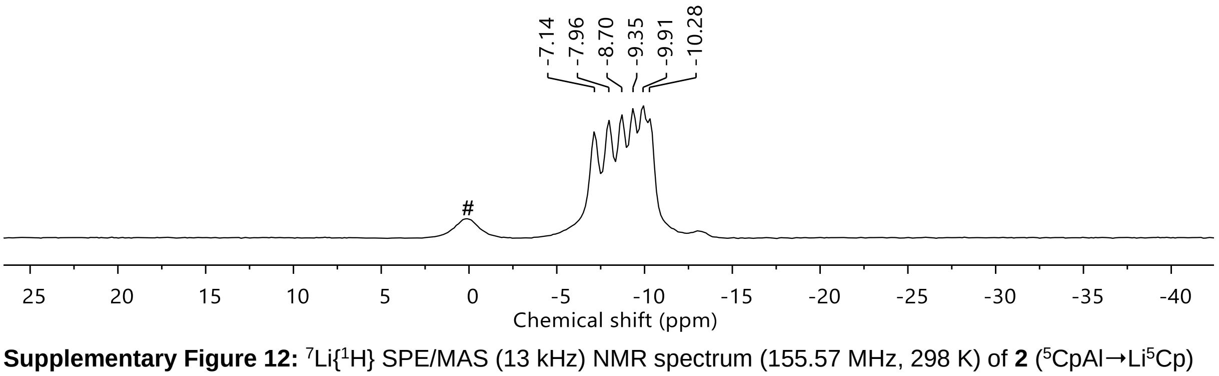

Supplementary Figure 12: $^{7}Li\{^{1}H\}$ SPE/MAS (13 kHz) NMR spectrum (155.57 MHz, 298 K) of **2** ($^{5}CpAl{\rightarrow}Li^{5}Cp$) (# decomposition product; note: the unusual signal shape originates from quadrupolar interactions).

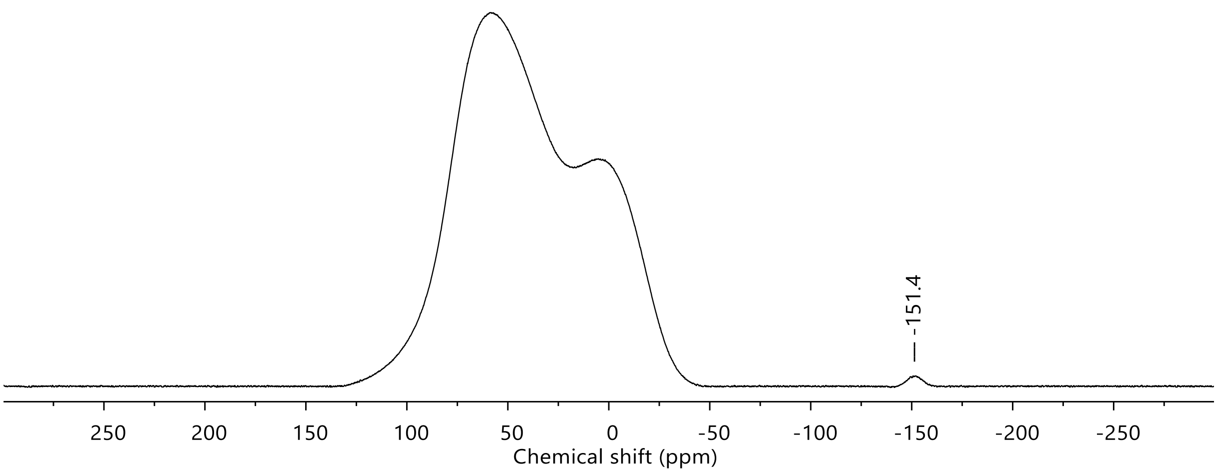

Supplementary Figure 13: $^{27}Al\{^{1}H\}$ NMR spectrum (104.26 MHz, C_6D_6, 293 K) of **2** ($^{5}CpAl \rightarrow Li^{5}Cp$).

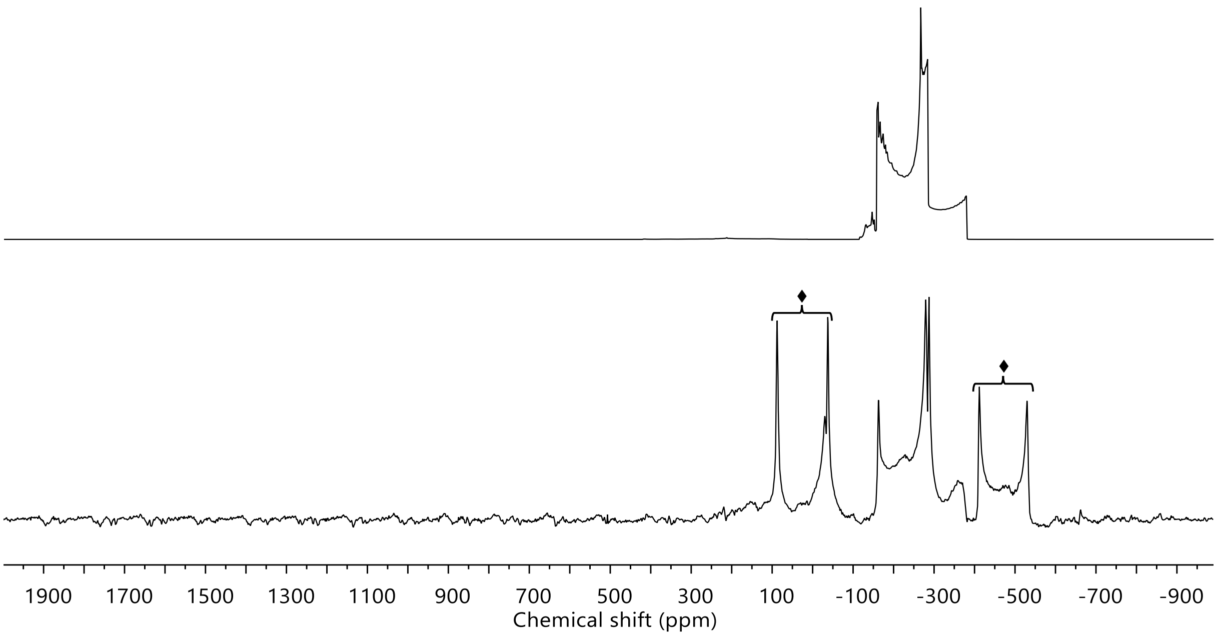

Supplementary Figure 14: ^{27}Al SPE/MAS(13 kHz) NMR spectrum (104.36 MHz, 297 K) of **2** ($^{5}CpAl \rightarrow Li^{5}Cp$) (top: simulated spectrum; bottom: experimental spectrum; analysis and simulations were performed with the Bruker TopSpin 3.6.4 software suite; ♦ spinning sidebands).

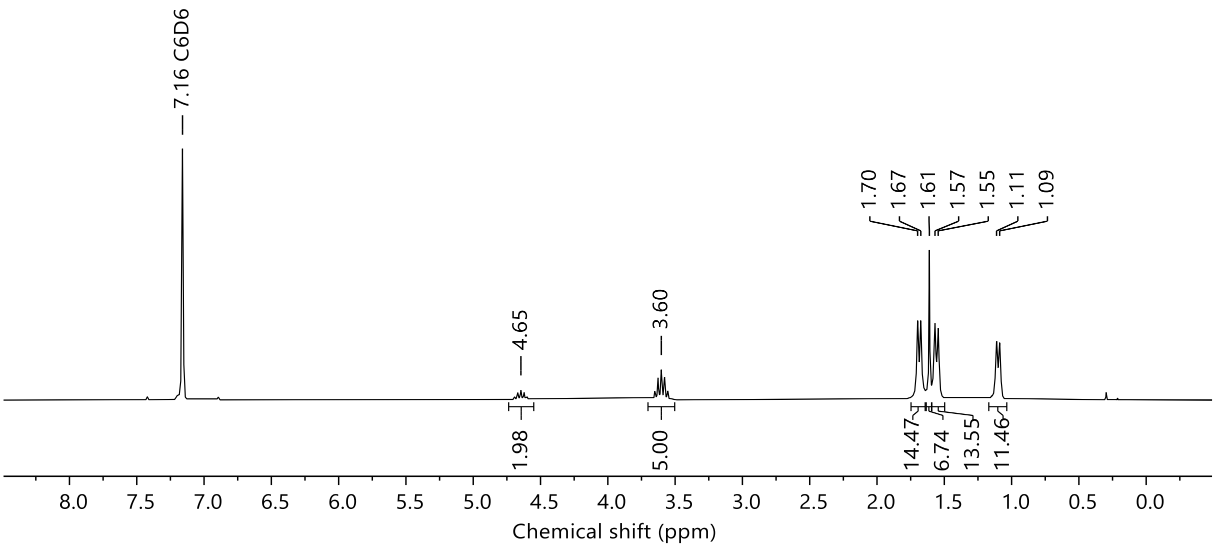

Supplementary Figure 15: ^{1}H NMR spectrum (400.13 MHz, C_6D_6, 298 K) of **3** (^{5}CpLi·NHC).

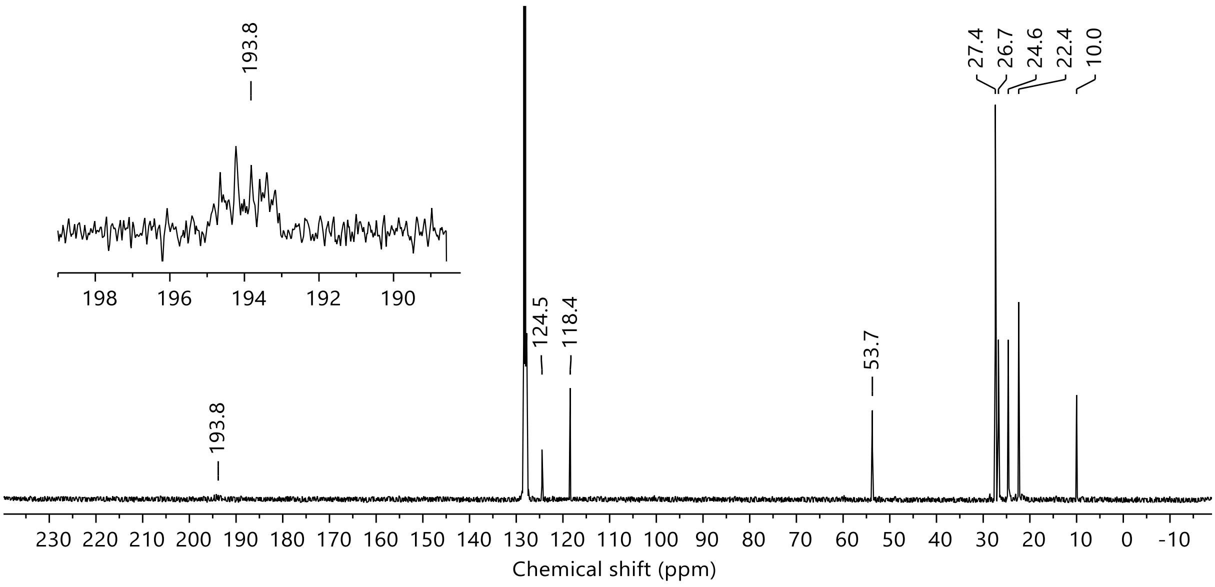

Supplementary Figure 16: $^{13}C\{^{1}H\}$ NMR spectrum (100.62 MHz, C_6D_6, 299 K) of **3** (^{5}CpLi·NHC).

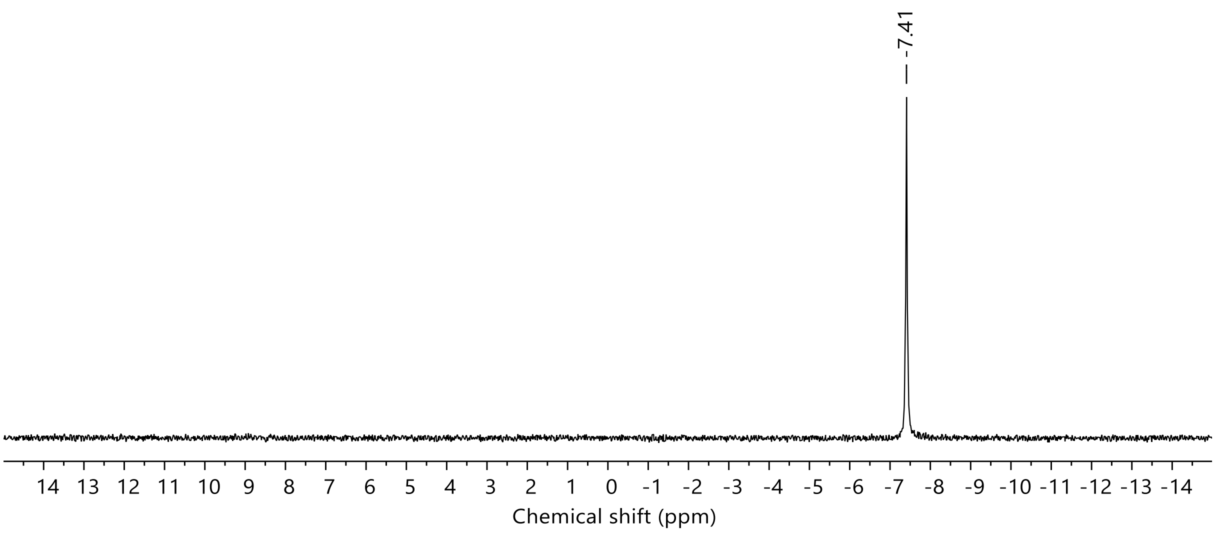

Supplementary Figure 17: ^{7}Li NMR spectrum (116.64 MHz, C_6D_6, 298 K) of **3** (^{5}CpLi•NHC).

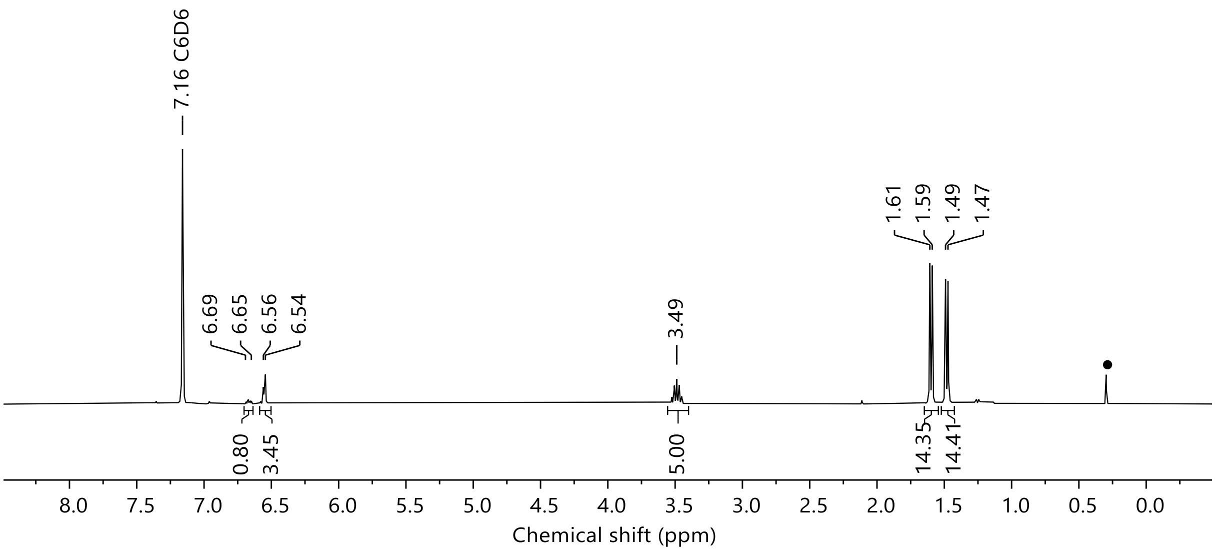

Supplementary Figure 18: ^{1}H NMR spectrum (400.13 MHz, C_6D_6, 298 K) of **4a** (^{5}CpLi·CNPh) (• silicon grease).

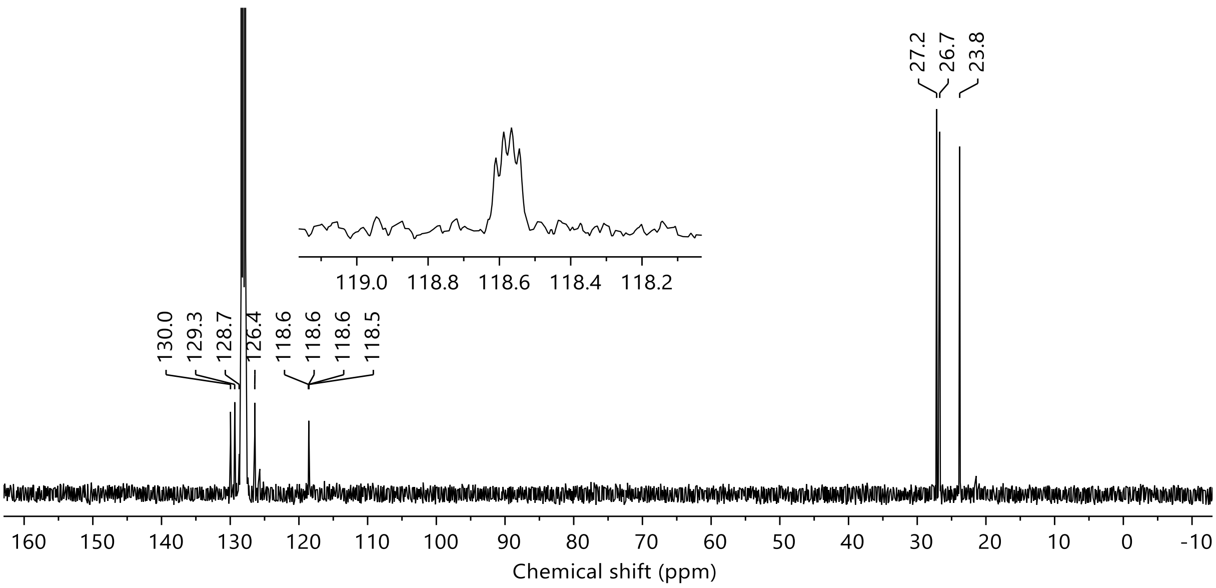

Supplementary Figure 19: $^{13}C\{^{1}H\}$ NMR spectrum (100.62 MHz, C_6D_6, 299 K) of **4a** (^{5}CpLi•CNPh).

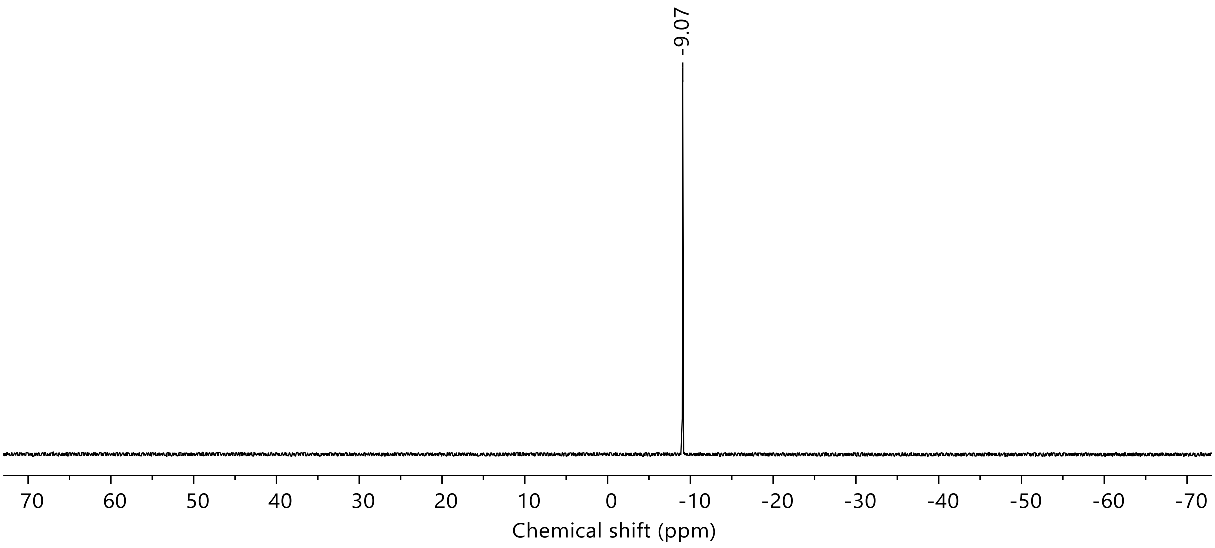

Supplementary Figure 20: ^{7}Li NMR spectrum (155.51 MHz, C_6D_6, 298 K) of **4a** (^{5}CpLi•CNPh).

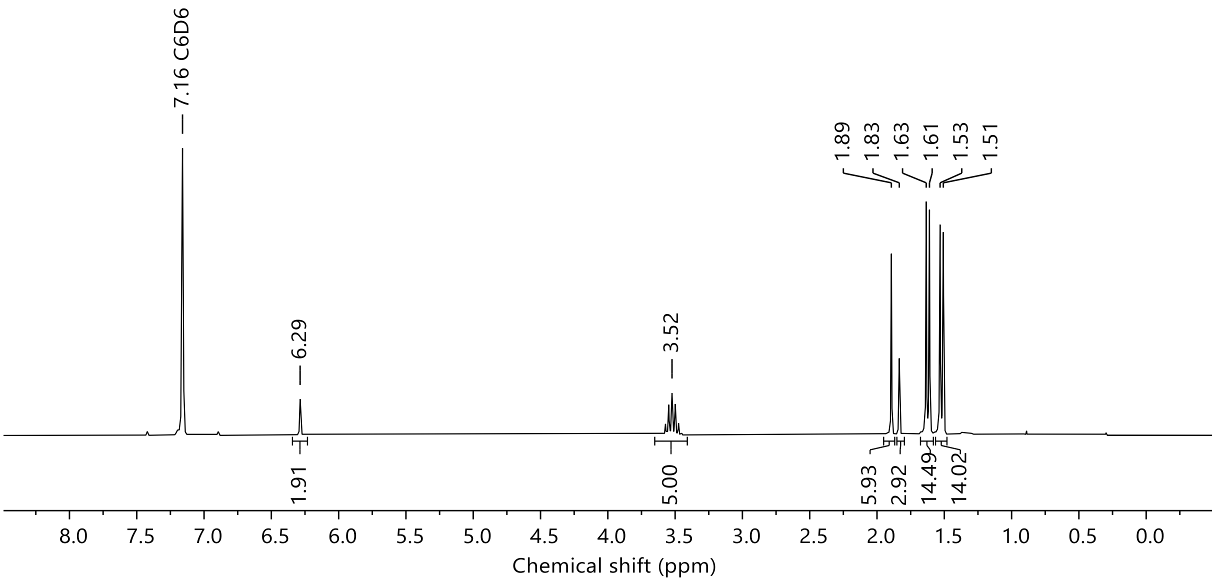

Supplementary Figure 21: ^{1}H NMR spectrum (300.13 MHz, C_6D_6, 297 K) of **4b** (^{5}CpLi•CNMes).

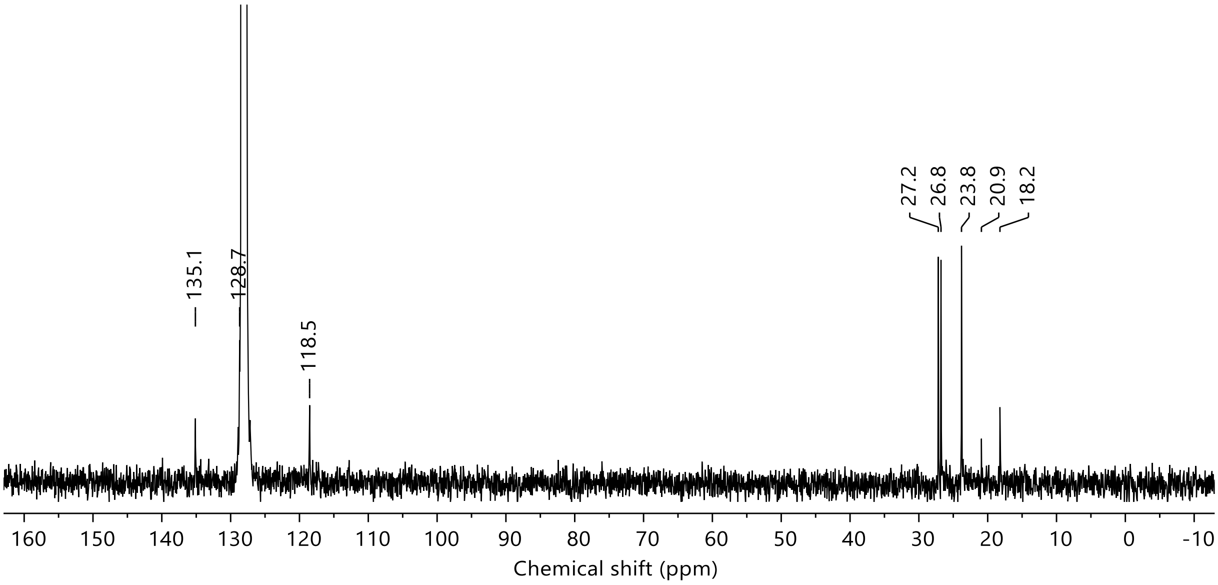

Supplementary Figure 22: ^{13}C{^{1}H} NMR spectrum (75.48 MHz, C_6D_6, 297 K) of **4b** (^{5}CpLi•CNMes).

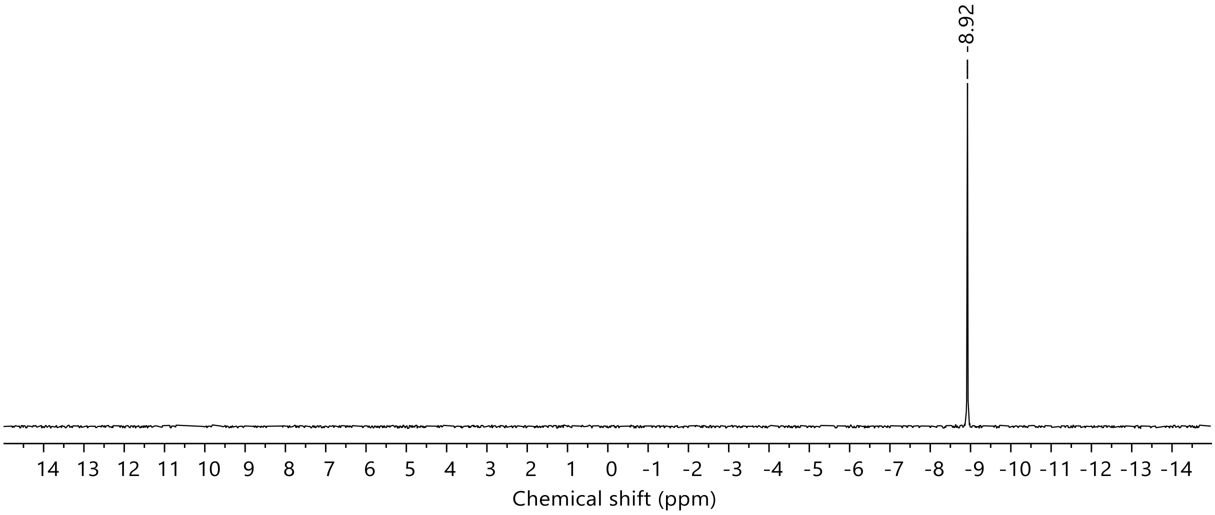

Supplementary Figure 23: ^{7}Li NMR spectrum (116.64 MHz, C_6D_6, 297 K) of **4b** (^{5}CpLi·CNMes).

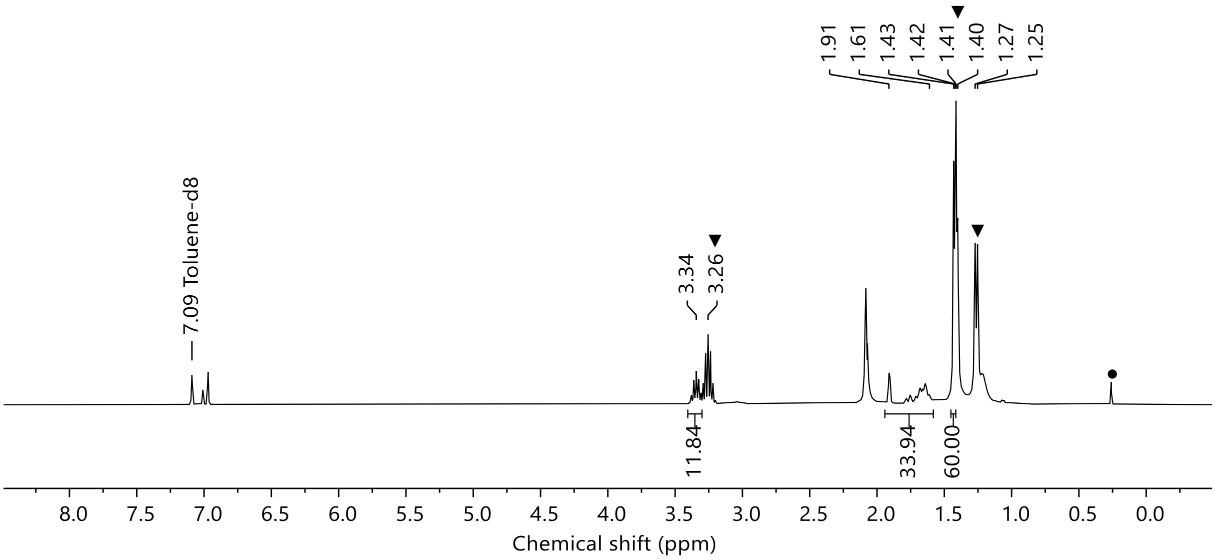

Supplementary Figure 24: ^{1}H NMR spectrum (400.13 MHz, C_7D_8, 297 K) of the reaction mixture of **2** + AdN_3 (▼ ^{5}CpLi, ● silicon grease).

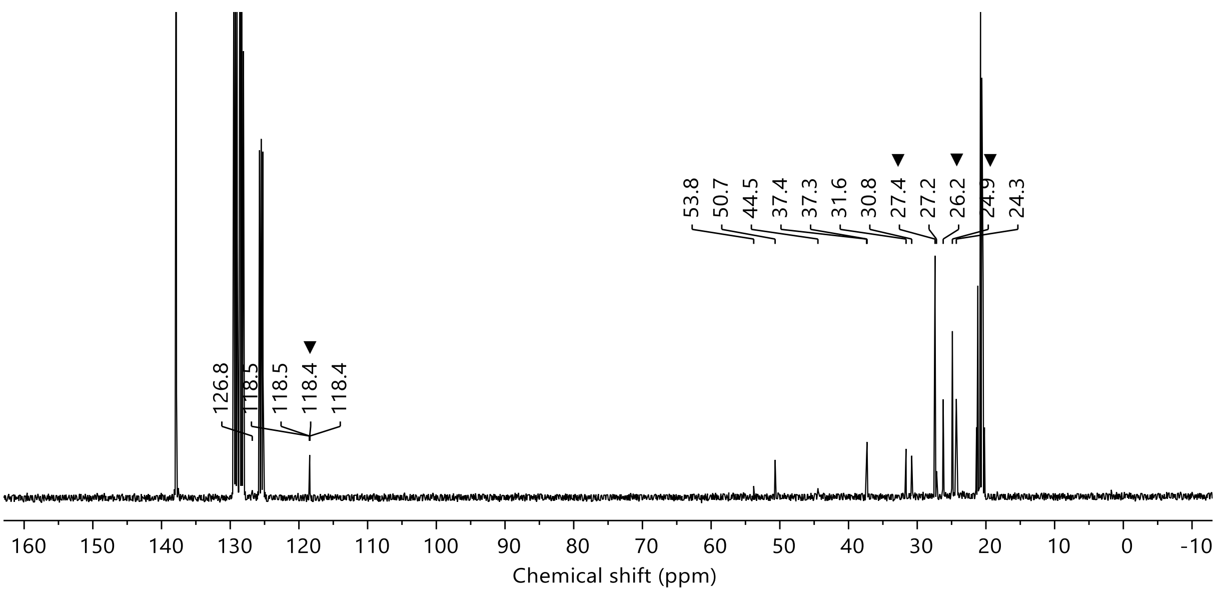

Supplementary Figure 25: $^{13}C\{^{1}H\}$ NMR spectrum (100.62 MHz, C_7D_8, 298 K) of the reaction mixture of **2** + AdN_3 (▼ ^{5}CpLi).

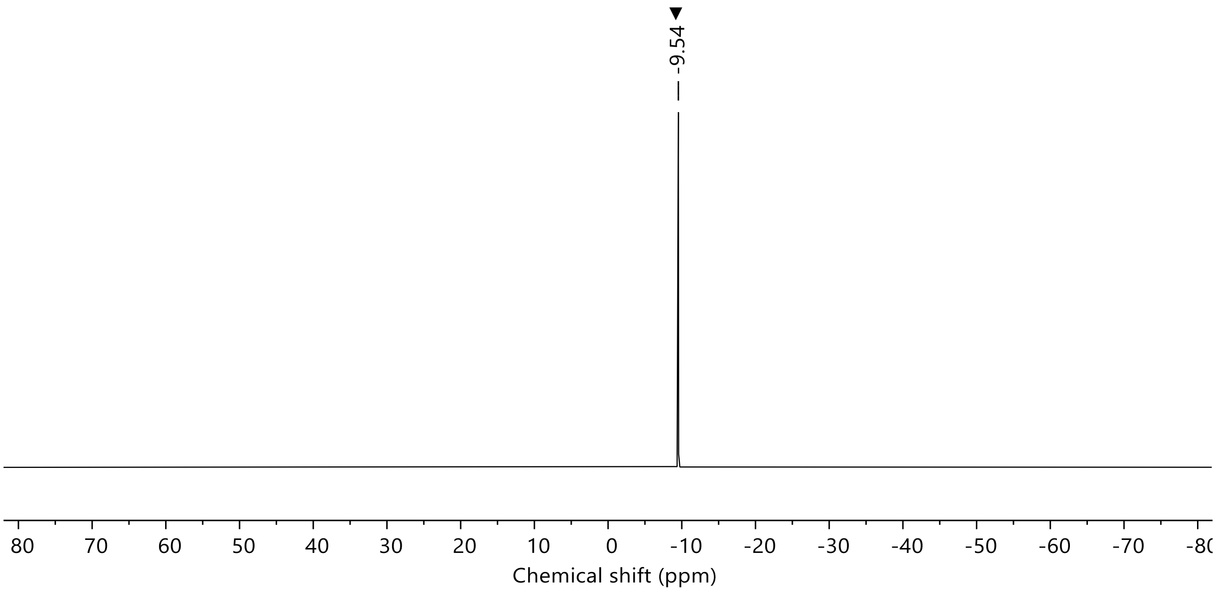

Supplementary Figure 26: ^{7}Li NMR spectrum (155.51 MHz, C_7D_8, 297 K) of the reaction mixture of **2** + AdN_3 (▼ ^{5}CpLi).

UV-Vis Spectra

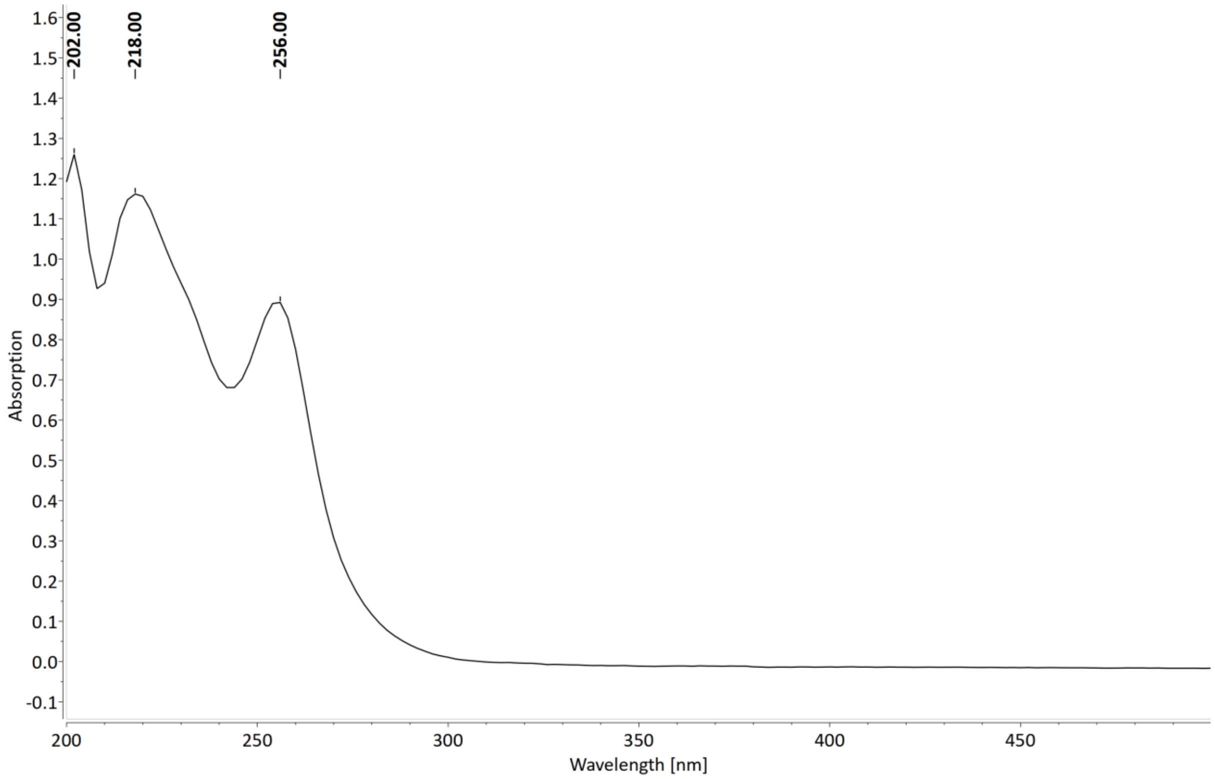

Supplementary Figure 27: UV-Vis spectrum of **1** (^{5}CpAl) (c = 1.47*10^{-4} mol L^{-1} in hexane).

IR Spectra

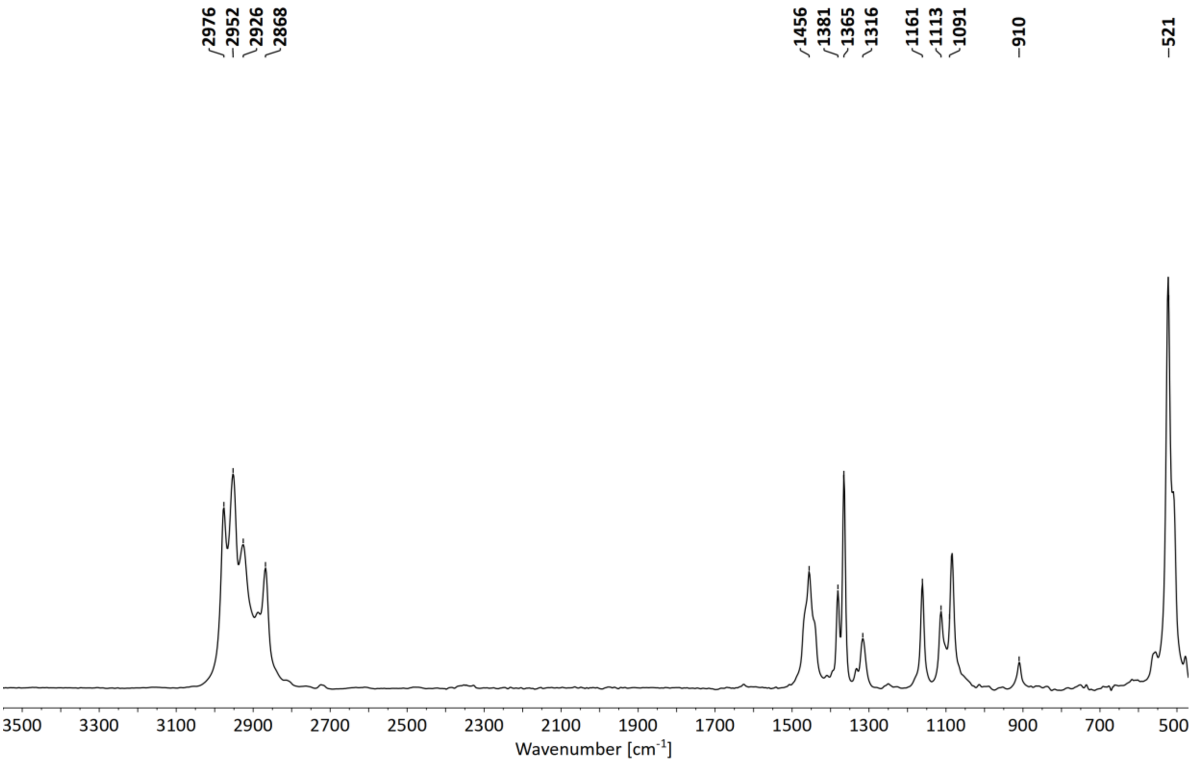

Supplementary Figure 28: IR spectrum of **1** (^{5}CpAl).

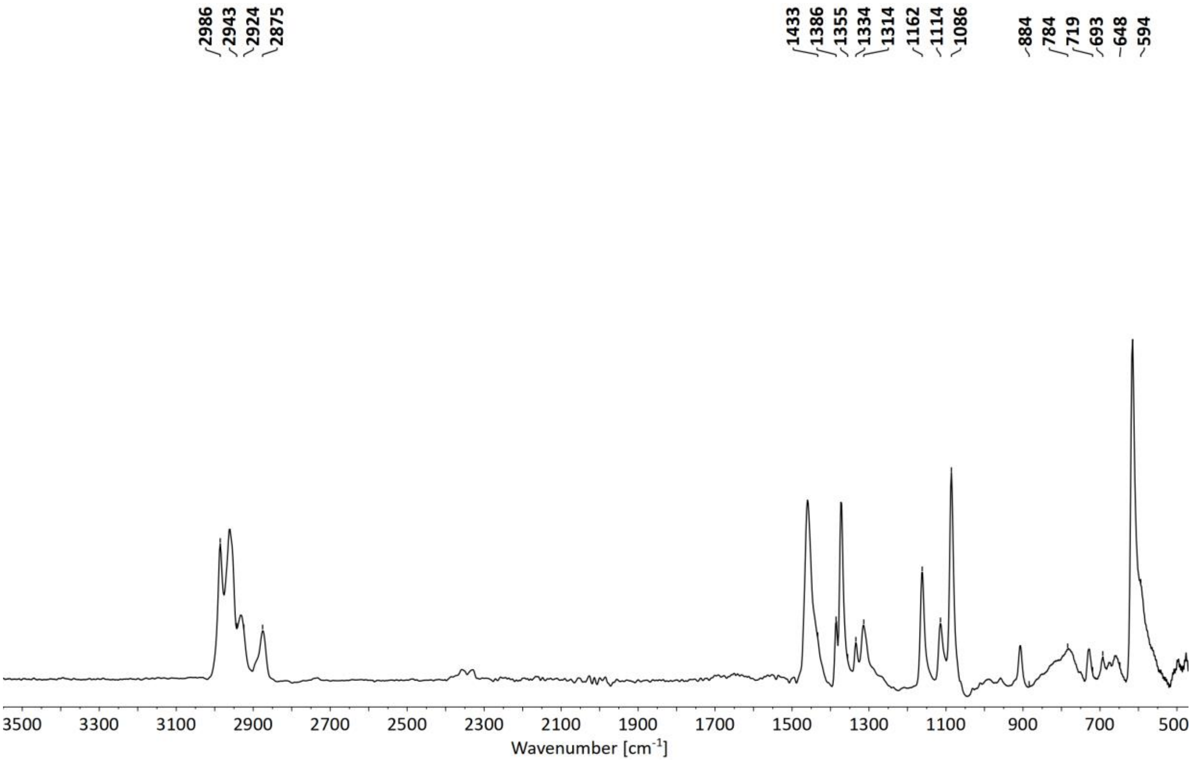

Supplementary Figure 29: IR spectrum of **1·AlBr$_3$** (^{5}CpAl→AlBr$_3$).

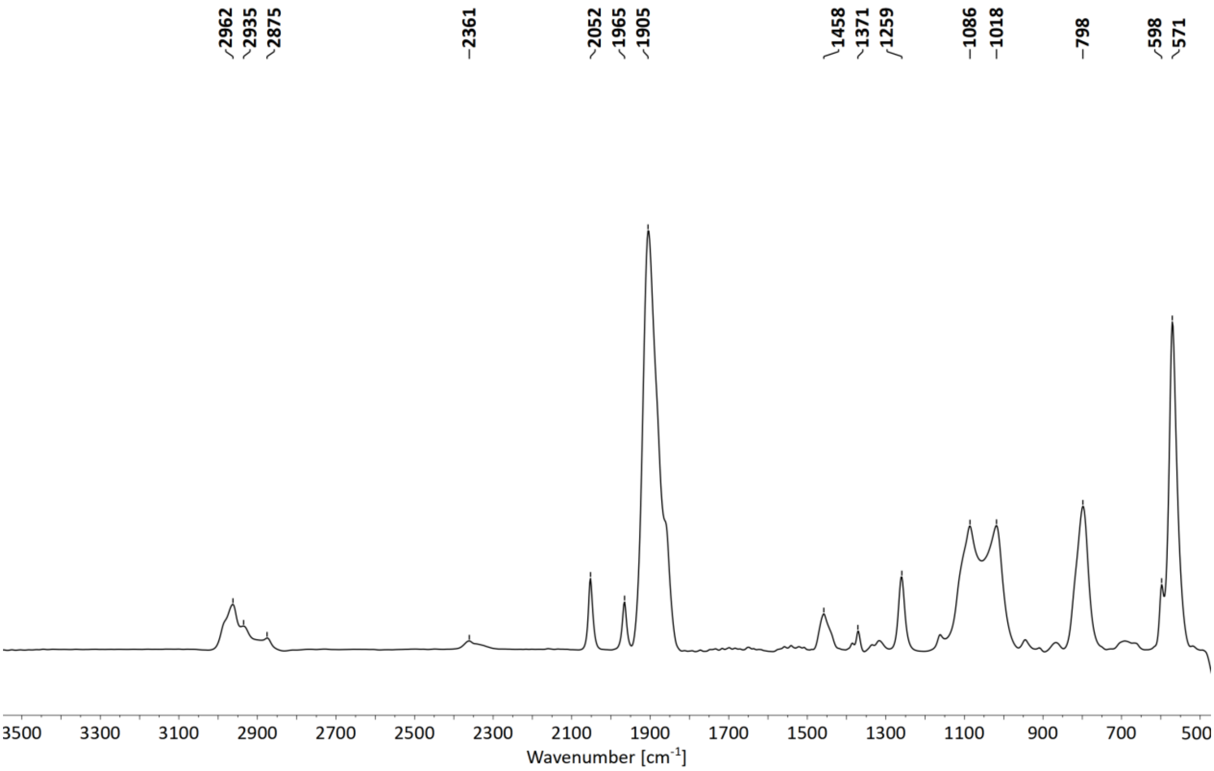

Supplementary Figure 30: IR spectrum of **1·W(CO)$_5$** (^{5}CpAl→W(CO)$_5$).

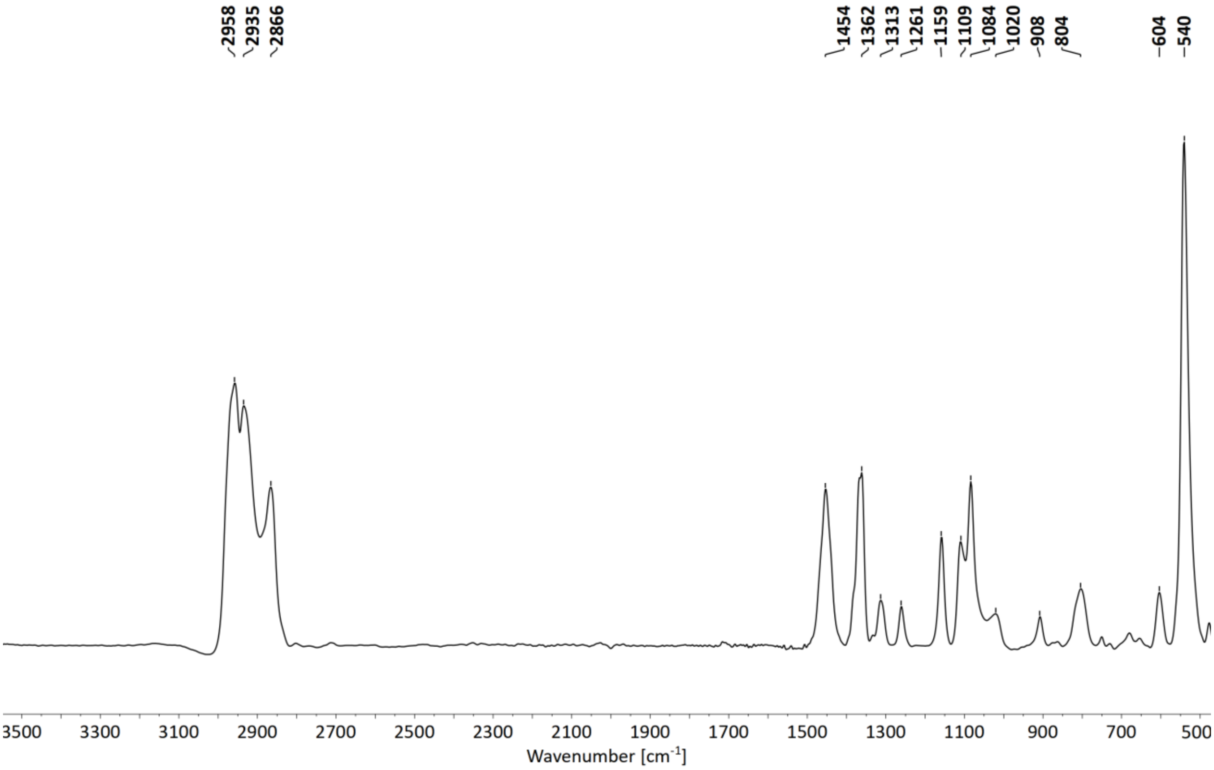

Supplementary Figure 31: IR spectrum of **2** (^{5}CpAl→Li^{5}Cp).

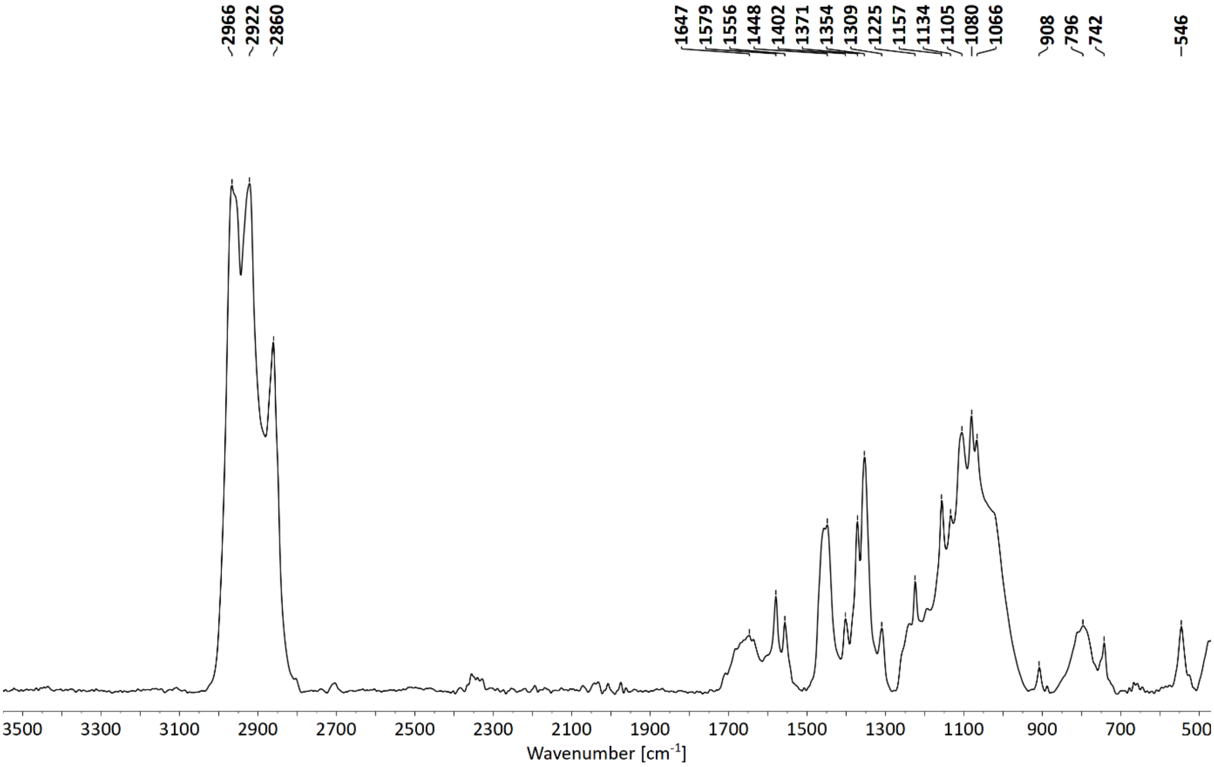

Supplementary Figure 32: IR spectrum of **3** (^{5}CpLi·NHC).

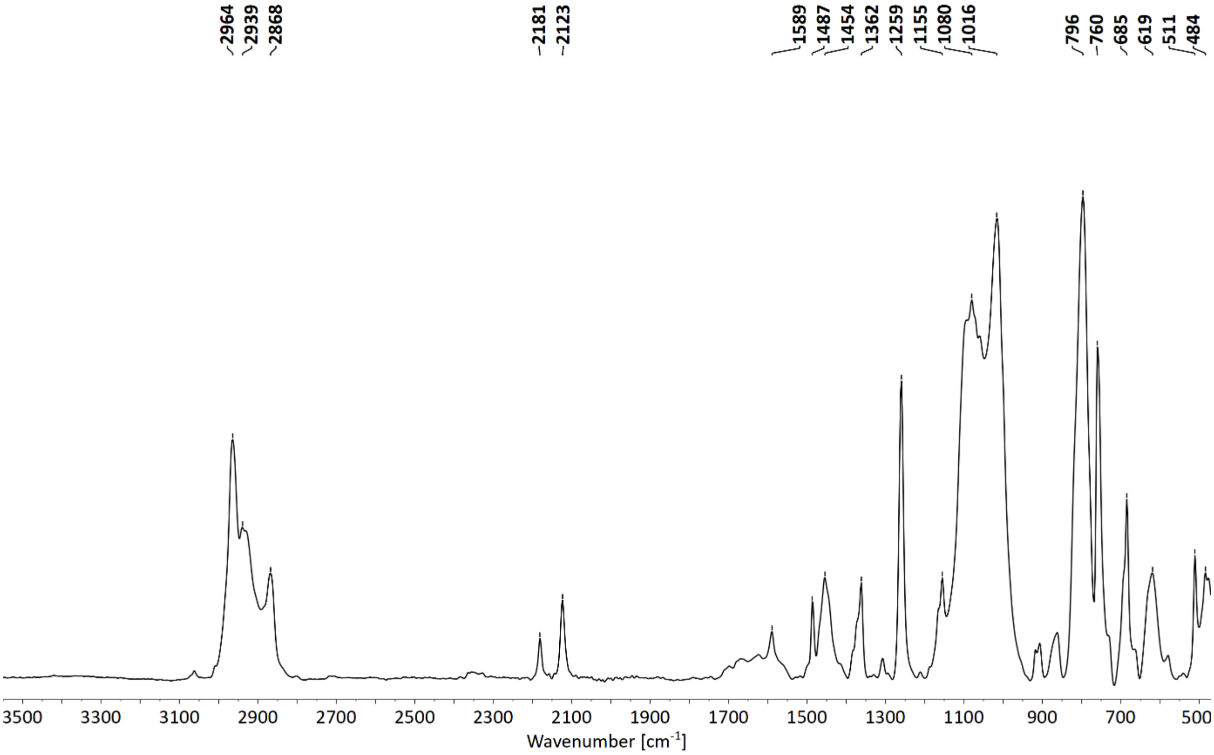

Supplementary Figure 33: IR spectrum of **4a** (^{5}CpLi·CNPh).

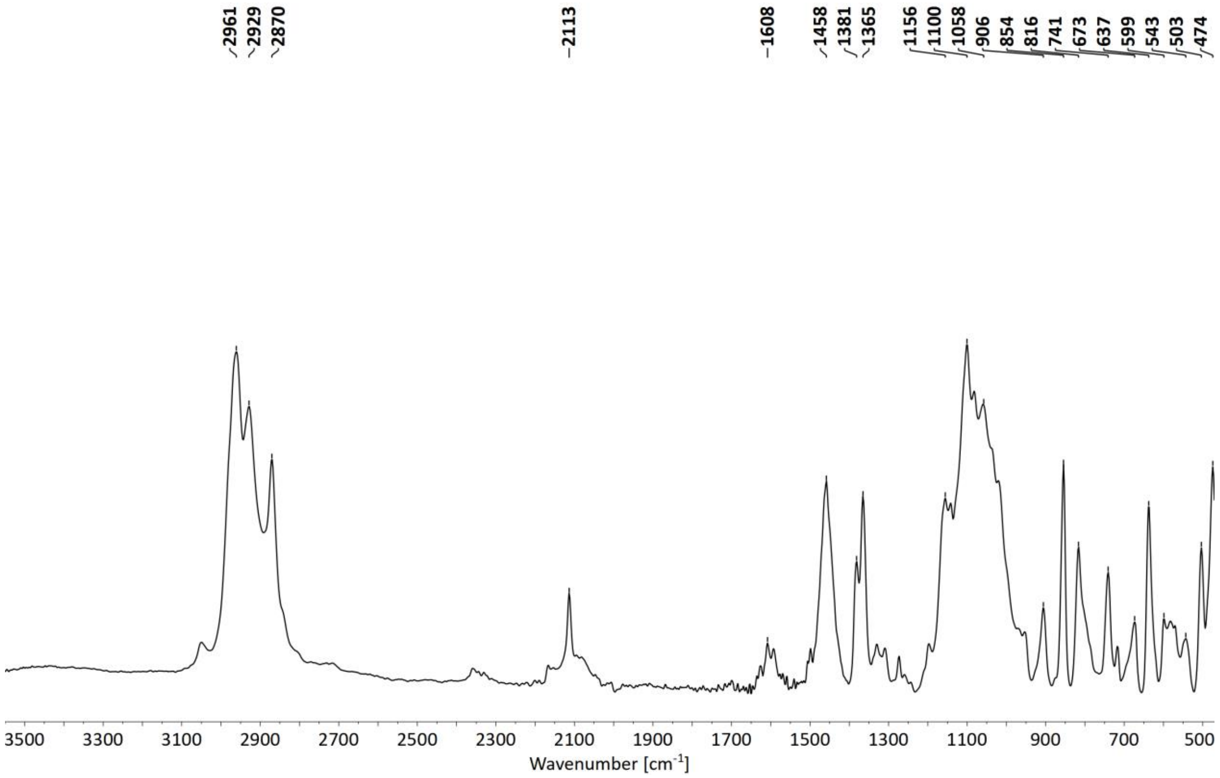

Supplementary Figure 34: IR spectrum of **4b** (^{5}CpLi·CNMes).

XRD Data

General Information:

Structure solution was conducted with direct methods using SHELXT and refinement by full matrix least squares calculations on F^2 using SHELXL2018 in the graphical user interface Shelxle.[2] Crystal structure data has been deposited with the Cambridge Crystallographic Data Centre (CCDC) and is available free of charge from the Cambridge Structural Database (see reference numbers).

Structural and refinement details for 1 (^{5}CpAl):

CCDC number	2279422	
Empirical formula	$C_{20}H_{35}Al$	
Formula weight	302.46	
Temperature	144(2) K	
Wavelength	1.54178 Å	
Crystal system	orthorhombic	
Space group	*Pbcm*	
Unit cell dimensions	a = 8.7996(2) Å	α = 90°
	b = 14.4009(3) Å	β = 90°
	c = 16.0978(4) Å	γ = 90°
Volume	2039.95(8) Å^3	
Z	4	
Density (calculated)	0.985 mg m^{-3}	
Absorption coefficient	0.792 mm^{-1}	
F(000)	672	
Crystal size	0.100 x 0.100 x 0.010 mm^3	
Theta range for data collection	5.026 to 70.137°	
Index ranges	-10<=h<=9, -17<=k<=15, -19<=l<=19	
Reflections collected	31665	
Independent reflections	2021 [R(int) = 0.0742]	
Completeness to theta = 67.679°	100.0%	
Absorption correction	semi-empirical from equivalents	
Max. and min. transmission	0.7533 and 0.6617	
Refinement method	full-matrix least-squares on F^2	
Data / restraints / parameters	2021 / 67 / 197	
Goodness-of-fit on F^2	1.081	
Final R indices [I>2σ(I)]	R1 = 0.0397, wR2 = 0.1184	
R indices (all data)	R1 = 0.0544, wR2 = 0.1270	
Extinction coefficient	n/a	
Largest diff. peak and hole	0.165 and -0.353 e.Å^{-3}	

The (pentaisopropyl)cyclopentadienyl ring is split over two positions. The occupation factors of the two components were refined to 0.5.
A checkCIF B-level alert originates from a calculated crystal density slightly below 1 mg m^{-3}. This is however not uncommon for organo aluminium compounds.

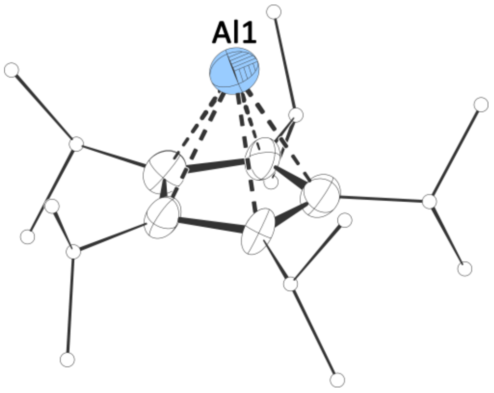

Supplementary Figure 35: Molecular structure of **1** (^{5}CpAl) in the crystal (displacement ellipsoids at 50% probability level, H atoms omitted for clarity, ^{i}Pr groups drawn as ball-and-stick models).

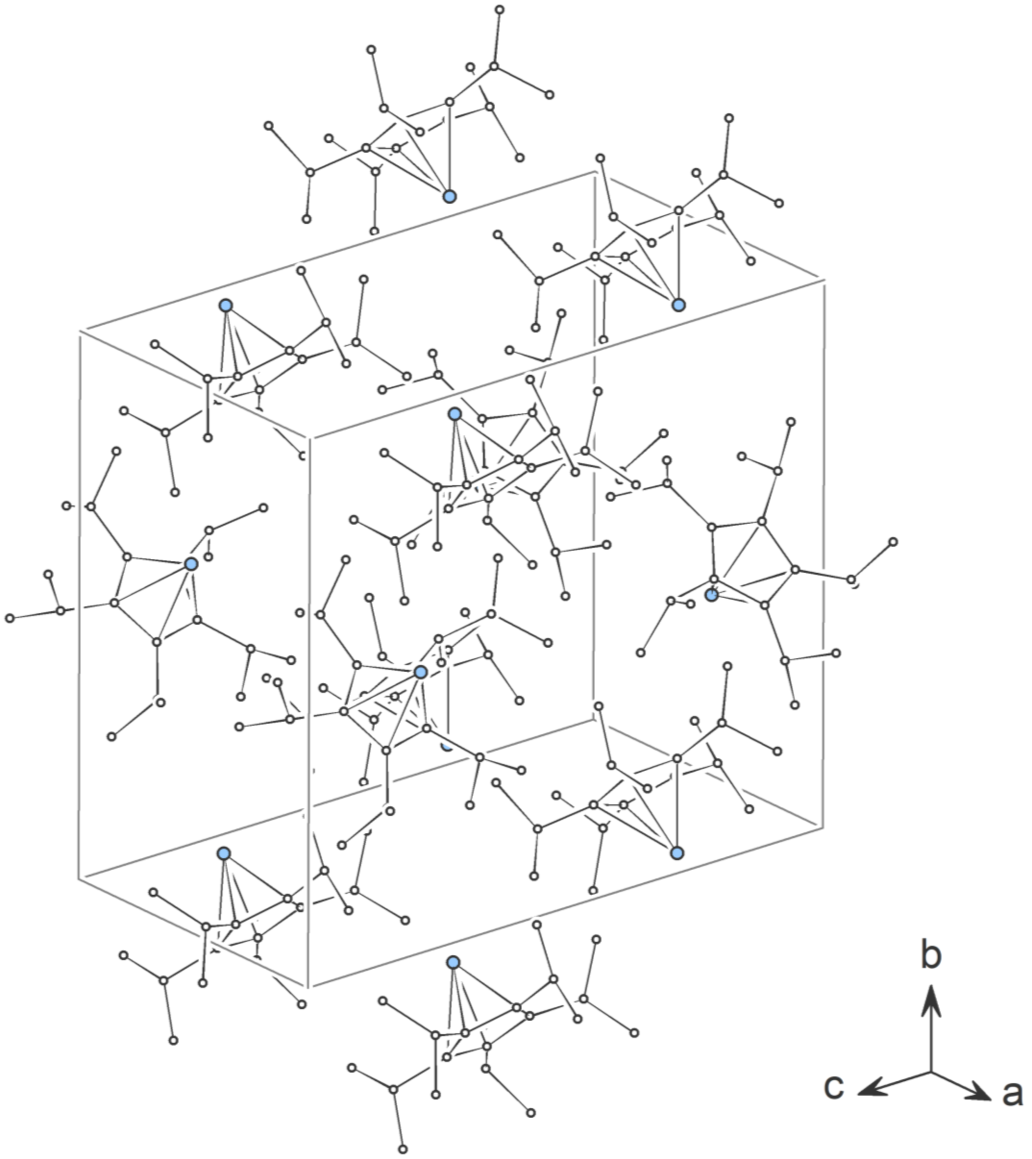

Supplementary Figure 36: Expanded unit cell of **1** (^{5}CpAl).

Structural and refinement details for 1·AlBr3 (^{5}CpAl→AlBr3):

CCDC number	2279423	
Empirical formula	$C_{20}H_{35}Al_2Br_3$	
Formula weight	569.17	
Temperature	152(2) K	
Wavelength	0.71073 Å	
Crystal system	monoclinic	
Space group	$P2_1/c$	
Unit cell dimensions	a = 17.4593(4) Å	α = 90°
	b = 9.4910(2) Å	β = 117.5130(10)°
	c = 17.1348(4) Å	γ = 90°
Volume	2518.23(10) $Å^3$	
Z	4	
Density (calculated)	1.501 mg m^{-3}	
Absorption coefficient	4.878 mm^{-1}	
F(000)	1144	
Crystal size	0.200 x 0.200 x 0.200 mm^3	
Theta range for data collection	1.315 to 28.781°	
Index ranges	-20<=h<=23, -12<=k<=12, -23<=l<=20	
Reflections collected	28104	
Independent reflections	6544 [R(int) = 0.0358]	
Completeness to theta = 25.242°	100.0%	
Absorption correction	semi-empirical from equivalents	
Max. and min. transmission	0.7458 and 0.6639	
Refinement method	full-matrix least-squares on F^2	
Data / restraints / parameters	6544 / 0 / 236	
Goodness-of-fit on F^2	1.030	
Final R indices [I>2σ(I)]	R1 = 0.0366, wR2 = 0.0741	
R indices (all data)	R1 = 0.0586, wR2 = 0.0810	
Extinction coefficient	n/a	
Largest diff. peak and hole	1.802 and -1.425 e.$Å^{-3}$	

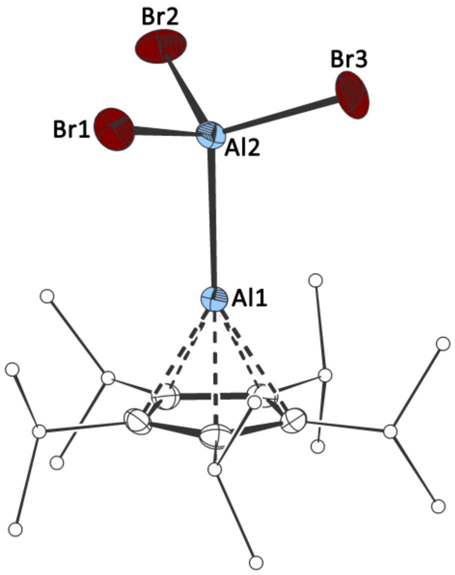

Supplementary Figure 37: Molecular structure of **1·AlBr3** in the crystal (displacement ellipsoids at 50% probability level, H atoms omitted for clarity, ^{i}Pr groups drawn as ball-and-stick models).

Structural and refinement details for 1·W(CO)$_5$ (^{5}CpAl→W(CO)$_5$):

CCDC number	2279424
Empirical formula	$C_{25}H_{35}AlO_5W$
Formula weight	626.36
Temperature	143(2) K
Wavelength	0.71073 Å
Crystal system	monoclinic
Space group	$P2_1/n$
Unit cell dimensions	a = 10.0880(2) Å α = 90°
	b = 15.6745(3) Å β = 93.2060(10)°
	c = 17.1467(3) Å γ = 90°
Volume	2707.07(9) Å^3
Z	4
Density (calculated)	1.537 mg m^{-3}
Absorption coefficient	4.330 mm^{-1}
F(000)	1248
Crystal size	0.200 x 0.120 x 0.100 mm^3
Theta range for data collection	2.288 to 28.713°
Index ranges	-13<=h<=13, -21<=k<=20, -23<=l<=23
Reflections collected	55227
Independent reflections	7005 [R(int) = 0.0329]
Completeness to theta = 25.242°	100.0%
Absorption correction	semi-empirical from equivalents
Max. and min. transmission	0.7458 and 0.6524
Refinement method	full-matrix least-squares on F^2
Data / restraints / parameters	7005 / 950 / 490
Goodness-of-fit on F^2	1.046
Final R indices [I>2σ(I)]	R1 = 0.0188, wR2 = 0.0390
R indices (all data)	R1 = 0.0230, wR2 = 0.0406
Extinction coefficient	n/a
Largest diff. peak and hole	2.391 and -0.764 e.Å^{-3}

The (pentaisopropyl)cyclopentadienyl ring is split over two positions. The occupation factors for the major component was refined to 0.56.

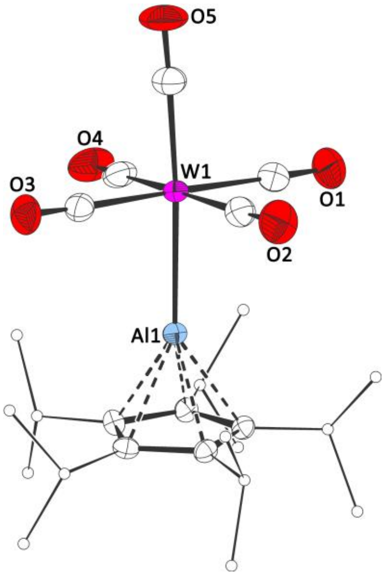

Supplementary Figure 38: Molecular structure of **1·W(CO)$_5$** in the crystal (displacement ellipsoids at 50% probability level, H atoms omitted for clarity, ^{i}Pr groups drawn as ball-and-stick models).

Structural and refinement details for 2 (^{5}CpAl→Li^{5}Cp):

CCDC number	2279425		2324314	
Empirical formula	$C_{52}H_{78}AlF_4Li$		$C_{54}H_{86}AlLi$	
Formula weight	813.06		769.14	
Temperature	143(2) K		152(2) K	
Wavelength	1.54178 Å		0.71073 Å	
Crystal system	orthorhombic		triclinic	
Space group	*Pbca*		*P*1	
Unit cell dimensions	a = 13.783(2) Å	α = 90°	a = 10.4914(7) Å	α = 64.641(2)°
	b = 15.079(2) Å	β = 90°	b = 11.7124(8) Å	β = 76.932(2)°
	c = 23.824(3) Å	γ = 90°	c = 11.8882(8) Å	γ = 77.347(2)°
Volume	4951.4(12) Å^3		1273.42(15) Å^3	
Z	4		1	
Density (calculated)	1.091 mg m^{-3}		1.003 mg m^{-3}	
Absorption coefficient	0.723 mm^{-1}		0.071 mm^{-1}	
F(000)	1768		426	
Crystal size	0.200 x 0.160 x 0.080 mm^3		0.300 x 0.240 x 0.200 mm^3	
Theta range for data collection	3.711 to 74.488°		1.943 to 27.164°	
Index ranges	-16<=h<=16, -18<=k<=18, -29<=l<=29		-13<=h<=13, -15<=k<=15, -11<=l<=15	
Reflections collected	82794		18471	
Independent reflections	5041 [R(int) = 0.0339]		8916 [R(int) = 0.0315]	
Completeness to theta = 67.679°	99.9%		99.9%	
Absorption correction	semi-empirical from equivalents		semi-empirical from equivalents	
Max. and min. transmission	0.7538 and 0.6436		0.7455 and 0.6902	
Refinement method	full-matrix least-squares on F^2		full-matrix least-squares on F^2	
Data / restraints / parameters	5041 / 2861 / 531		8916 / 1626 / 916	
Goodness-of-fit on F^2	1.031		1.006	
Final R indices [I>2σ(I)]	R1 = 0.0381, wR2 = 0.1130		R1 = 0.0445, wR2 = 0.0886	
R indices (all data)	R1 = 0.0409, wR2 = 0.1156		R1 = 0.0772, wR2 = 0.1030	
Absolute structure parameter			0.2(2)	
Extinction coefficient	n/a		n/a	
Largest diff. peak and hole	0.191 and -0.187 e.Å^{-3}		0.148 and -0.150 e.Å^{-3}	

Two different crystal structures of **2** could be obtained, one co-crystallized with 1,2-difluorobenzene and one co-crystallized with toluene.

In the first structure (2279425), the (pentaisopropyl)cyclopentadienyl ring is split over two positions. The occupation factors for the major component was refined to 0.58. The occupation factors of Al1 and Li1 were constraint to 0.5. To refine the disorder Al1, Li1, C7/C7a, C8/C8a, C10/C10a, C11/C11a, C13/C13a, C14/C14a, C16/C16a, C17/17a, C19/C19a and C20/C20a were treated with EADP. Incorporated 1,2-difluorobenzene was split over three positions and the occupation factors were refined to 0.56, 0.31 and 0.13.

A checkCIF A-level alert originates from the unusual bonding situation of the aluminium atom.

The second structure (2324314) was refined as an inversion twin. Both (pentaisopropyl)-cyclopentadienyl rings are split over two positions and the occupation factors for the major component was refined to 0.51. The occupation factors of Al1 and Li1 were refined to 0.93 and 0.07.

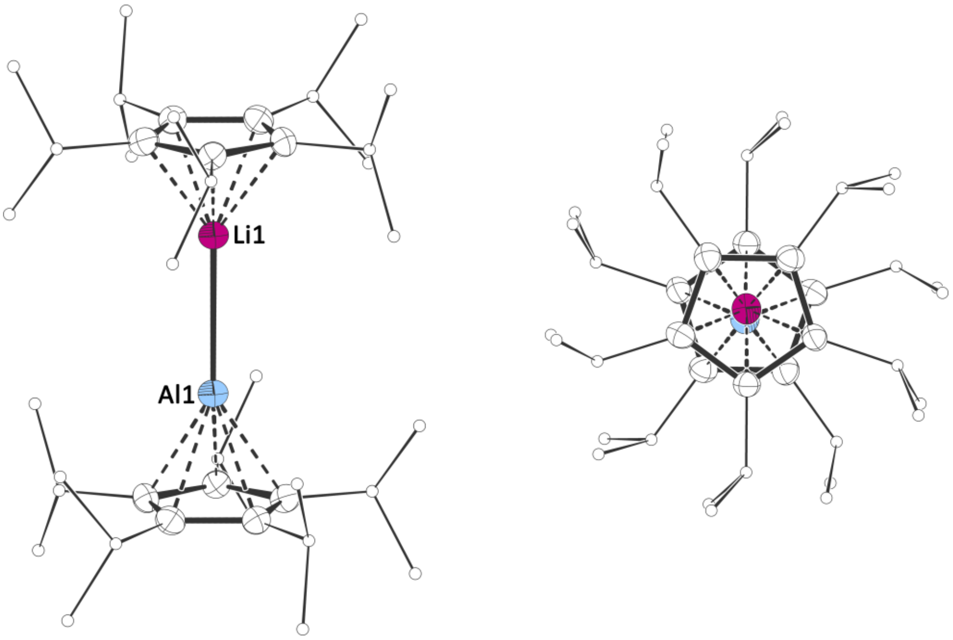

Supplementary Figure 39: Molecular structure of **2** (^{5}CpAl→Li^{5}Cp) in the crystal (side view and top view; displacement ellipsoids at 50% probability level, H atoms omitted for clarity, ^{i}Pr groups drawn as ball-and-stick models).

Structural and refinement details for 3 (^{5}CpLi·NHC):

CCDC number	2324317	
Empirical formula	$C_{83}H_{134}Li_2N_4$	
Formula weight	1201.81	
Temperature	143(2) K	
Wavelength	0.71073 Å	
Crystal system	triclinic	
Space group	*P*1	
Unit cell dimensions	a = 10.0714(2) Å	α = 105.7010(10)°
	b = 13.0656(3) Å	β = 92.6010(10)°
	c = 16.6962(3) Å	γ = 111.0150(10)°
Volume	1949.42(7) Å^3	
Z	1	
Density (calculated)	1.024 mg m^{-3}	
Absorption coefficient	0.058 mm^{-1}	
F(000)	666	
Crystal size	0.220 x 0.200 x 0.180 mm^3	
Theta range for data collection	2.194 to 28.528°	
Index ranges	-13<=h<=13, -17<=k<=17, -22<=l<=22	
Reflections collected	85672	
Independent reflections	9920 [R(int) = 0.0519]	
Completeness to theta = 25.242°	99.9%	
Absorption correction	semi-empirical from equivalents	
Max. and min. transmission	0.7457 and 0.7182	
Refinement method	full-matrix least-squares on F^2	
Data / restraints / parameters	9920 / 1513 / 772	
Goodness-of-fit on F^2	1.044	
Final R indices [I>2σ(I)]	R1 = 0.0523, wR2 = 0.1339	
R indices (all data)	R1 = 0.0686, wR2 = 0.1455	
Extinction coefficient	n/a	
Largest diff. peak and hole	0.212 and -0.413 e.Å^{-3}	

The (pentaisopropyl)cyclopentadienyl ring and two incorporated toluene molecules are split over two positions, with one of the toluene molecules arranged over a center of symmetry. The occupation factors were constraint to 0.5.

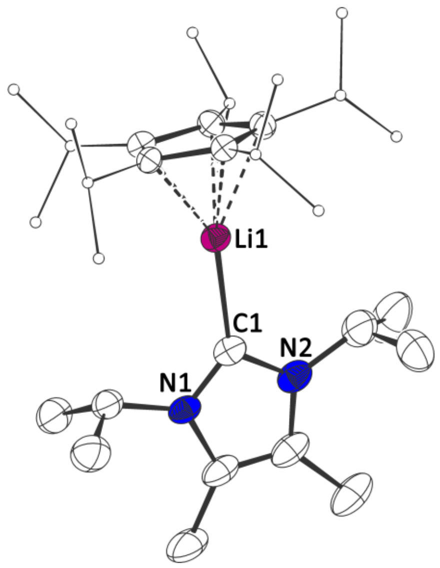

Supplementary Figure 40: Molecular structure of **3** (^{5}CpLi·NHC) in the crystal (displacement ellipsoids at 50% probability level, H atoms omitted for clarity, ^{i}Pr groups drawn as ball-and-stick models).

Structural and refinement details for 4a (^{5}CpLi·CNPh):

CCDC number	2324316	
Empirical formula	$C_{34}H_{48}LiN$	
Formula weight	477.67	
Temperature	143(2) K	
Wavelength	0.71073 Å	
Crystal system	monoclinic	
Space group	*C*2/c	
Unit cell dimensions	a = 12.6632(6) Å	α = 90°
	b = 13.3822(5) Å	β = 90°
	c = 18.6105(8) Å	γ = 90°
Volume	3153.8(2) Å^3	
Z	4	
Density (calculated)	1.006 mg m^{-3}	
Absorption coefficient	0.056 mm^{-1}	
F(000)	1048	
Crystal size	0.200 x 0.180 x 0.020 mm^3	
Theta range for data collection	2.189 to 25.678°	
Index ranges	-15<=h<=15, -16<=k<=16, -22<=l<=22	
Reflections collected	26625	
Independent reflections	3006 [R(int) = 0.0638]	
Completeness to theta = 25.242°	100.0%	
Refinement method	full-matrix least-squares on F^2	
Data / restraints / parameters	3006 / 687 / 400	
Goodness-of-fit on F^2	1.076	
Final R indices [I>2σ(I)]	R1 = 0.0437, wR2 = 0.1012	
R indices (all data)	R1 = 0.0664, wR2 = 0.1158	
Extinction coefficient	n/a	
Largest diff. peak and hole	0.111 and -0.143 e.Å^{-3}	

The structure was refined as a pseudo-merohedral twin (twin matrix: -1 0 0 0 -1 0 0 0 1) with an angle β very close to 90°.

The (pentaisopropyl)cyclopentadienyl lithium moiety lies on a crystallographic twofold-rotation axis which is not part of the molecular symmetry and was split over two positions. The occupation factors were constraint to 0.5. A co-crystalized toluene solvent molecule lies on a center of inversion and was split over two positions. The occupation factors for the major component was refined to 0.51 and the individual occupation factors of each part were constraint to 0.5.

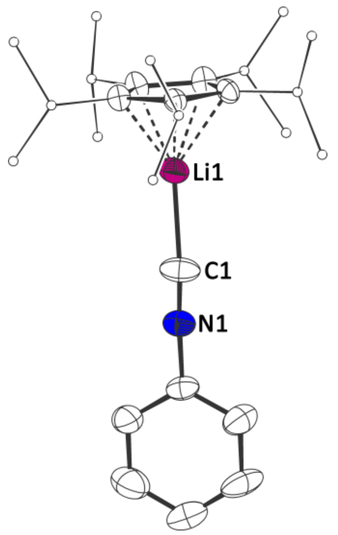

Supplementary Figure 41: Molecular structure of **4a** (^{5}CpLi·CNPh) in the crystal (displacement ellipsoids at 50% probability level, H atoms omitted for clarity, ^{i}Pr groups drawn as ball-and-stick models).

Structural and refinement details for 4b (^{5}CpLi·CNMes):

CCDC number	2338161	
Empirical formula	$C_{30}H_{46}LiN$	
Formula weight	427.62	
Temperature	143(2) K	
Wavelength	0.71073 Å	
Crystal system	monoclinic	
Space group	*C*2/c	
Unit cell dimensions	a = 16.0934(4) Å	α = 90°
	b = 9.8007(4) Å	β = 99.233(2)°
	c = 18.4964(5) Å	γ = 90°
Volume	2879.58(16) Å^3	
Z	4	
Density (calculated)	0.986 mg m^{-3}	
Absorption coefficient	0.055 mm^{-1}	
F(000)	944	
Crystal size	0.240 x 0.200 x 0.060 mm^3	
Theta range for data collection	2.231 to 25.024°	
Index ranges	-19<=h<=19, -11<=k<=11, -21<=l<=21	
Reflections collected	26005	
Independent reflections	2537 [R(int) = 0.0515]	
Completeness to theta = 25.024°	100.0%	
Absorption correction	semi-empirical from equivalents	
Max. and min. transmission	0.7454 and 0.7108	
Refinement method	full-matrix least-squares on F^2	
Data / restraints / parameters	2537 / 310 / 302	
Goodness-of-fit on F^2	1.173	
Final R indices [I>2sigma(I)]	R1 = 0.0682, wR2 = 0.1571	
R indices (all data)	R1 = 0.0765, wR2 = 0.1617	
Extinction coefficient	n/a	
Largest diff. peak and hole	0.133 and -0.184 e.Å^{-3}	

The entire molecule is disordered and was split over two positions. The occupation factors were constraint to 0.5.

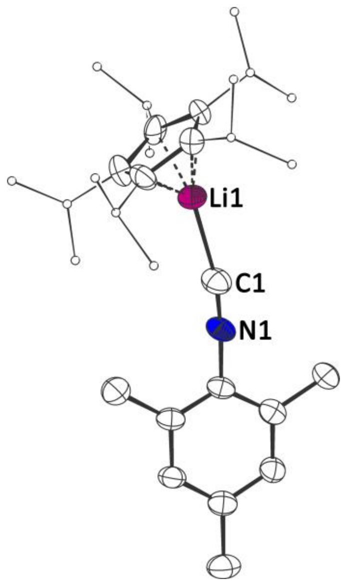

Supplementary Figure 42: Molecular structure of **4b** (^{5}CpLi•CNMes) in the crystal (displacement ellipsoids at 50% probability level, H atoms omitted for clarity, ^{i}Pr groups drawn as ball-and-stick models).

Structural and refinement details for 5 ($(^{5}CpAlNAd)_2$):

CCDC number	2338185
Empirical formula	$C_{60}H_{100}Al_2N_2$
Formula weight	903.37
Temperature	143(2) K
Wavelength	0.71073 Å
Crystal system	orthorhombic
Space group	*Pbca*
Unit cell dimensions	a = 14.4067(5) Å α = 90°
	b = 23.4935(8) Å β = 90°
	c = 31.5318(11) Å γ = 90°
Volume	10672.4(6) $Å^3$
Z	8
Density (calculated)	1.124 mg m^{-3}
Absorption coefficient	0.094 mm^{-1}
F(000)	4000
Crystal size	0.240 x 0.200 x 0.040 mm^3
Theta range for data collection	2.102 to 25.681°
Index ranges	-15<=h<=17, -28<=k<=28, -38<=l<=38
Reflections collected	148344
Independent reflections	10072 [R(int) = 0.1460]
Completeness to theta = 25.242°	99.5%
Absorption correction	semi-empirical from equivalents
Max. and min. transmission	0.7455 and 0.6743
Refinement method	full-matrix least-squares on F^2
Data / restraints / parameters	10072 / 587 / 751
Goodness-of-fit on F^2	1.029
Final R indices [I>2σ(I)]	R1 = 0.0589, wR2 = 0.1231
R indices (all data)	R1 = 0.1041, wR2 = 0.1487
Extinction coefficient	0.00038(6)
Largest diff. peak and hole	0.391 and -0.289 e.$Å^{-3}$

One (pentaisopropyl)cyclopentadienyl moiety was split over two positions. The occupation factor for the major component was refined to 0.84.

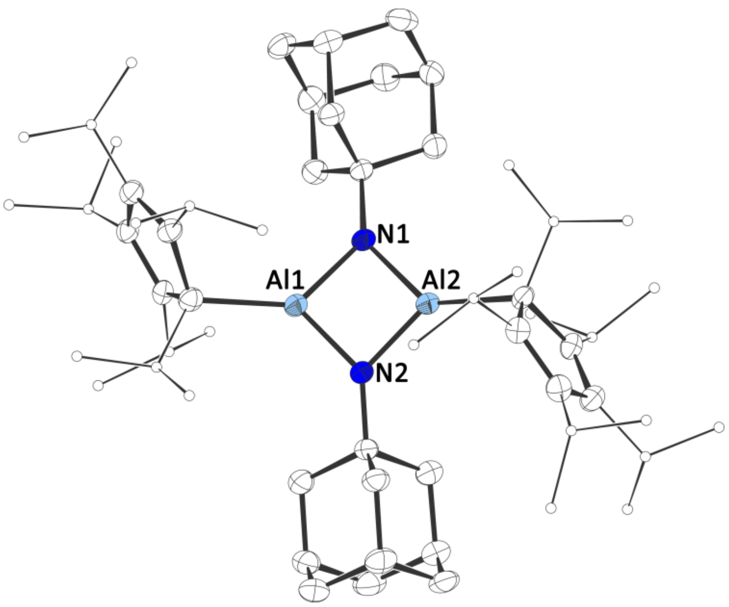

Supplementary Figure 43: Molecular structure of **5** ($\{^{5}CpAlNAd\}_2$) in the crystal (displacement ellipsoids at 50% probability level, H atoms omitted for clarity, ^{i}Pr groups drawn as ball-and-stick models).

Supplementary Table 1: Selected bond length for {Cp*Al}$_4$, **1**, **1·AlBr$_3$**, **1·W(CO)$_5$**, **2**, **3**, **4a**,**b** and **5**.

	Al–C^{Cp} [pm]	Al–Cp^{centroid} [pm]	Li–C^{Cp} [pm]	Li–Cp^{centroid} [pm]
(Cp*Al)$_4$[5]	229.3(1); 229.7(1); 230.8(9); 231.2(9); 231.6(1); 231.9(1); 231.9(1); 232.6(1); 233.1(1); 233.2(8); 233.2(1); 233.8(1); 233.8(1); 234.1(1); 235.1(9); 235.3(1); 235.8(8); 236.4(1); 237.2(1); 237.8(1)	199.8(2); 201.5(3); 201.7(2); 203.2(3)		
**1** (^{5}CpAl)	226.9(7); 229.5(7); 230.7(3); 232.7(7); 235.7(8)	196.7(6)		
**1·AlBr$_3$**	215.5(2); 215.8(3); 216.0(1); 216.4(1); 216.4(3)	178.3(9)		
**1·W(CO)$_5$**	219.2(8); 219.4(9); 220.1(7); 221.1(8); 221.5(7)	183.5(6)		
**2** (^{5}CpAl→Li^{5}Cp) (co-crystalized with 1,2-difluorobenzene)	225.1(9); 225.7(5); 225.9(1); 226.5(7); 226.5(5); 226.7(6); 227.0(7); 227.3(5); 227.9(9); 228.3(1)	191.1(1)	209.3(2); 210.4(2); 210.4(2); 211.2(1); 212.4(1)	172.1(1)
**2** (^{5}CpAl→Li^{5}Cp) (co-crystalized with toluene)	222.1(1); 223.2(1); 223.8(9); 225.4(1); 226.2(1); 223.8(3); 223.8(4); 224.2(1); 224.4(1); 224.9(3)	188.6(1); 188.8(3)	213.0(2); 213.5(2); 214.1(2); 214.9(2); 215.7(1); 213.0(3); 213.4(4); 214.0(3); 214.6(4); 214.8(1)	176.1(1); 176.6(1)
**3** (^{5}CpLi·NHC)			218.7(4); 218.9(5); 221.3(4); 222.4(1); 224.4(1)	184.9(2)
**4a** (^{5}CpLi·CNPh)			201.3(2); 207.4(1); 207.4(2); 214.2(2); 215.6(1)	170.6(5)
**4b** (^{5}CpLi·CNMes)			180.9(1); 187.4(1); 196.0(1); 202.2(1): 208.2(1)	153.1(1)
**5** ({^{5}CpAlNAd}$_2$)	202.5(3); 202.8(1)			

Computational Details

Geometry optimizations were performed using the Gaussian 16 C01 software suite.[6] All geometry optimizations were computed using the BP86,[7,8] B3LYP,[9,10] PBE0,[11,12] M06-2X,[13] ωB97XD,[14] B2PLYP,[15] and PWPB95[16] functionals, with the basis set def2-SVP.[17] Grimme's third generation dispersion correction terms D3[18] and the Becke-Johnson damping function[19] have been used for BP86, B3LYP and PBE0. Additionally, MP2 calculations have been performed using resolution of identity (RI)[20-22] with the cc-pVDZ basis set.[23] Stationary points were located with the Berny algorithm[24] using redundant internal coordinates. Analytical Hessians were computed to determine the nature of stationary points (one and zero imaginary frequencies for transition states and minima, respectively)[25] and to calculate unscaled zero-point energies (ZPEs) as well as thermal corrections and entropy effects using the standard statistical-mechanics relationships for an ideal gas.

The atomic partial charges were estimated with the natural bond orbital (NBO)[26,27] method, using NBO 7.0.[28] The topological quantum theory of atoms in molecules (QTAIM),[29] and Laplacian of the electron density analyses were carried out with AIMAII.[30] All these analyses were performed at the M06-2X/def2-TZVPP level of theory.

The nature of the chemical bonds were investigated by means of the Energy Decomposition Analysis (EDA) method,[31,32] which was developed by Morokuma[33] and by Ziegler and Rauk.[34,35] The bonding analysis focuses on the instantaneous interaction energy ΔE_{int} of a bond A–B between two fragments A and B in the particular electronic reference state and in the frozen geometry AB. This energy is divided into four main components (Eq1).

$$\Delta E_{\text{int}} = \Delta E_{elst} + \Delta E_{Pauli} + \Delta E_{orb} + \Delta E_{disp} \qquad \text{(Eq1)}$$

The term ΔE_{elst} corresponds to the quasi-classical electrostatic interaction between the unperturbed charge distributions of the prepared atoms (or fragments) and it is usually attractive. The Pauli repulsion ΔE_{Pauli} is the energy change associated with the transformation from the superposition of the unperturbed wave functions (Slater determinant of the Kohn-Sham orbitals) of the isolated fragments to the wave function Ψ_0 = NÂ[ΨAΨB], which properly obeys the Pauli principle through explicit antisymmetrization (Â operator) and renormalization (N = constant) of the product wave function. It comprises the destabilizing interactions between electrons of the same spin on either fragment. The orbital interaction ΔE_{orb} accounts for charge transfer and polarization effects.[36] In the case that the Grimme dispersion corrections[17,18] are computed the term ΔE_{disp} is added to equation S1. Further details on the EDA method can be found in the literature.[37,38] In the case of the dimers, relaxation of the fragments to their equilibrium geometries at the electronic ground state is termed ΔE_{prep}, because it may be considered as preparation energy for chemical bonding. The addition of ΔE_{prep} to the intrinsic interaction energy ΔE_{int} gives the total energy ΔE, which is, by definition, the opposite sign of the bond dissociation energy D_e:

$$\Delta E(-D_e) = \Delta E_{\text{int}} + \Delta E_{prep} \qquad \text{(Eq2)}$$

The EDA-NOCV method combines the EDA with the natural orbitals for chemical valence (NOCV) to decompose the orbital interaction term ΔE_{orb} into pairwise contributions. The NOCVs Ψi are defined as the eigenvector of the valence operator, $\hat{V}$, given by Equation (Eq3).

$$\hat{V}\Psi_i = v_i \Psi_i \quad \text{(Eq3)}$$

In the EDA-NOCV scheme the orbital interaction term, ΔE_{orb}, is given by Equation (Eq4),

$$\Delta E_{orb} = \sum_k \Delta E_k = \sum_{k=1}^{N/2} v_k \left[-F_{-k,k}^{TS} + F_{k,k}^{TS} \right] \quad \text{(Eq4)}$$

in which $F_{-k,-k}^{TS}$ and $F_{k,k}^{TS}$ are diagonal transition state Kohn–Sham matrix elements corresponding to NOCVs with the eigenvalues –vk and vk, respectively. The ΔE_k^{orb} term for a particular type of bond is assigned by visual inspection of the shape of the deformation density Δρk. The latter term is a measure of the size of the charge deformation, and it provides a visual notion of the charge flow that is associated with the pairwise orbital interaction. The EDA-NOCV scheme thus provides both qualitative and quantitative information about the strength of orbital interactions in chemical bonds. The EDA-NOCV calculations were carried out with ADF2019.101. The basis sets for all elements have triple-ζ quality augmented by two sets of polarizations functions and one set of diffuse function. Core electrons were treated by the frozen-core approximation. This level of theory is denoted BP86-D3(BJ)/TZ2P.[39] Scalar relativistic effects have been incorporated by applying the zeroth-order regular approximation (ZORA).[40]

Supplementary Table 2: Geometrical parameters of **2** with different DFAs (Density Functional Approximation) and ab-initio methods. Distances are given in pm and angles in degrees.

	Al–Li	Al–Cp^{centroid}	Li–Cp^{centroid}	Cp^{centroid}–Li–Al	Cp^{centroid}–Al–Li
X-ray	261.5(2)	188.6(1); 188.8(3)	176.1(1); 176.6(1)	176.6(8); 177.8(2)	176.6(3); 177.7(2)
BP86-D3BJ	255.9	190.5	169.7	179.9	179.9
B3LYP-D3BJ	257.4	190.7	169.3	179.9	179.8
PBE0-D3BJ	261.3	189.0	170.2	179.8	179.8
M06-2X	265.8	190.9	170.8	179.7	179.9
ωB97XD	263.8	188.1	172.1	180.0	180.0
B2PLYP	266.4	191.9	172.4	170.1	169.4
PWPB95	271.1	192.4	176.4	166.5	169.4
RI-MP2	258.8	193.5	171.0	179.8	179.7

* DFAs optimizations are carried out with def2-SVP basis set. RI-MP2 was carried out with cc-pVDZ basis set.

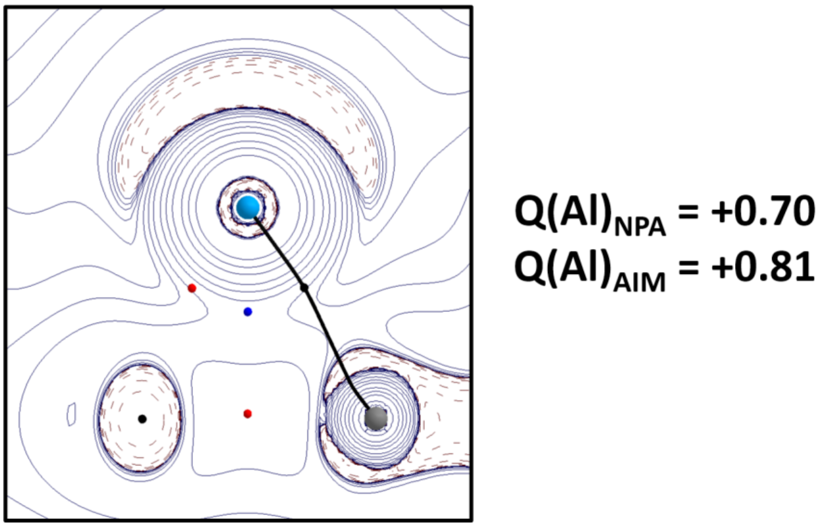

Supplementary Figure 44: Laplacian distribution of the electron density of **1** (contour line diagrams of the Laplacian distribution $\nabla^2\rho(r)$ in the Al–C–C plane. Dashed red lines indicate areas of charge concentration ($\nabla^2\rho(r)$<0), solid blue lines show areas of charge depletion ($\nabla^2\rho(r)$>0). Thick solid lines connecting the atomic nuclei are bond paths and small dots are the critical points, with bond critical points in black, ring critical points in red and cage critical point in blue.

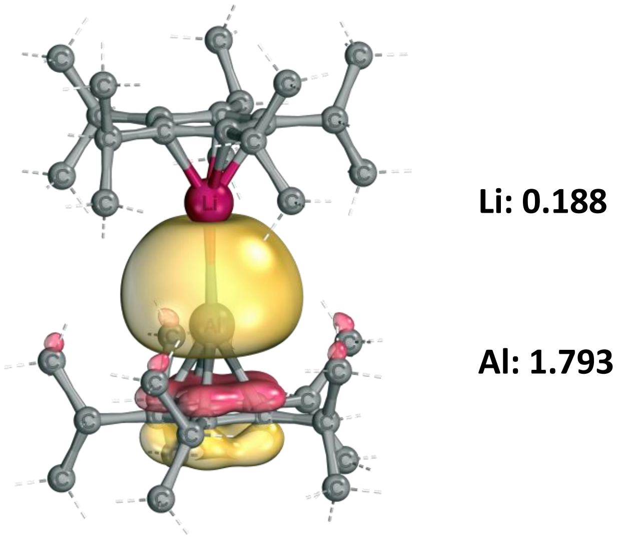

Supplementary Figure 45: Intrinsic Bond Orbitals (IBO: M06-2X/def2-SVP) of **2**.

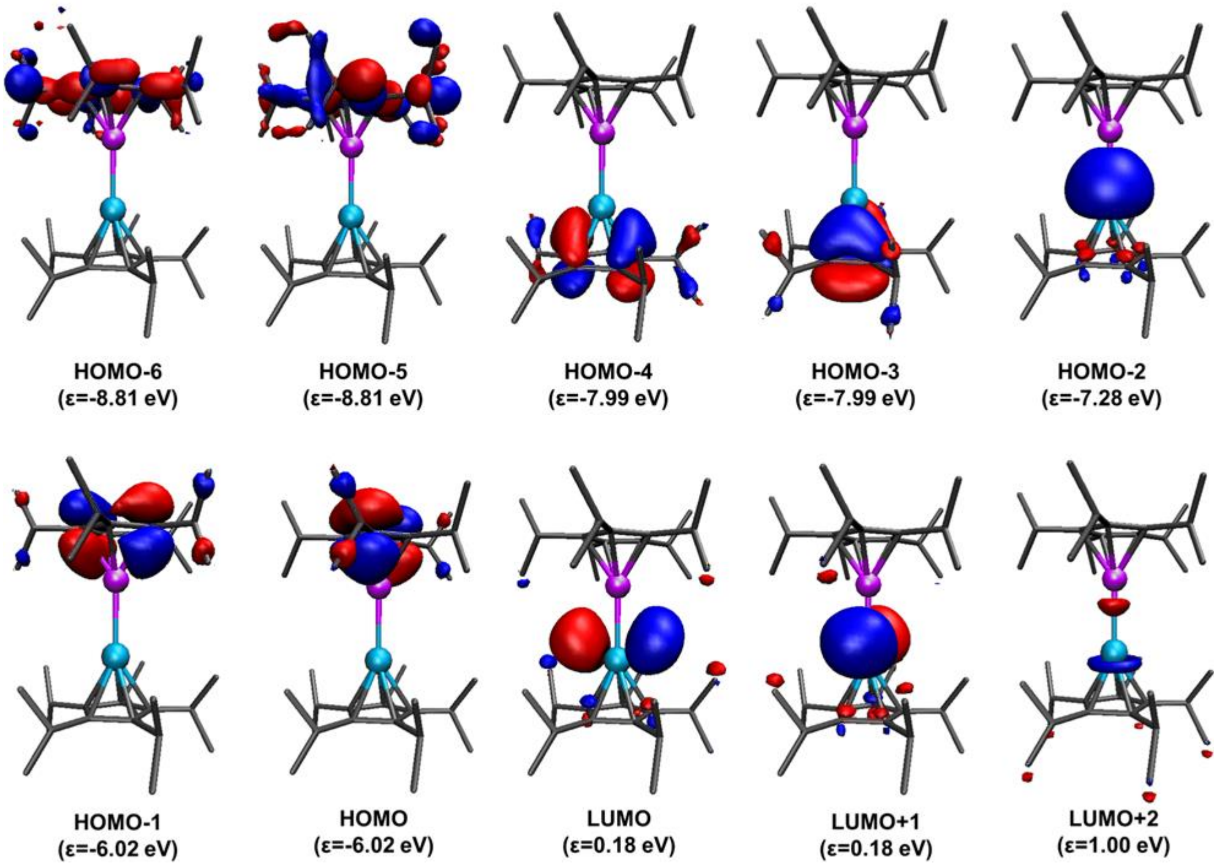

Supplementary Figure 46: Selected Kohn−Sham frontier molecular orbital contours of **2** (M06-2X/def2-TZVPP//M06-2X/def2-SVP; isodensity = 0.05 a.u.).

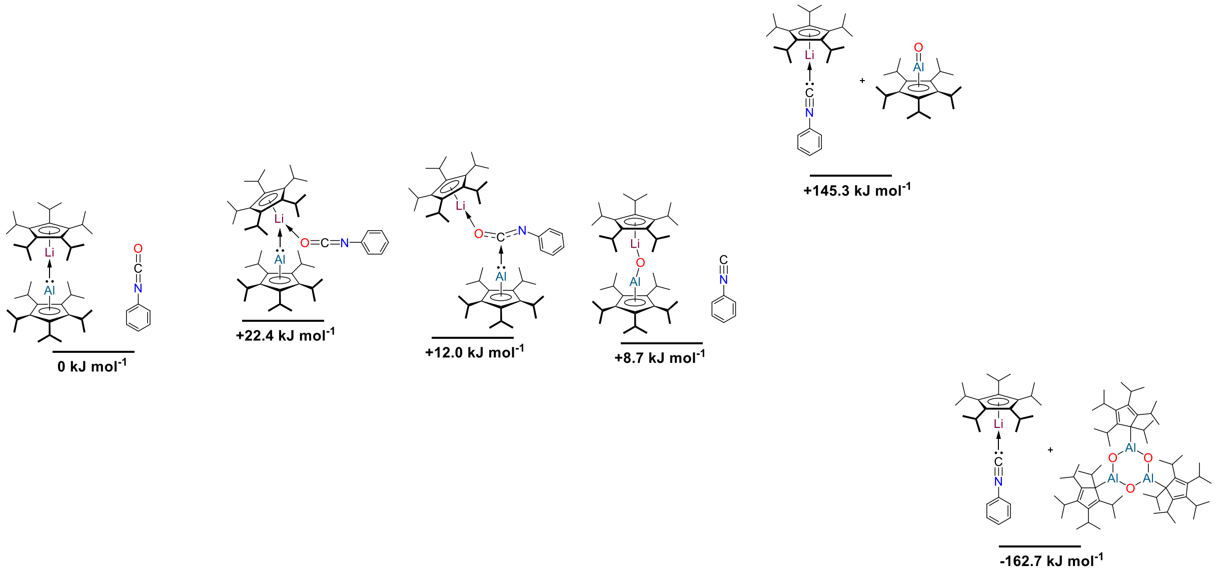

Supplementary Figure 47: Calculated relative energies of possible intermediates / products for the reaction of **2** + PhNCO (M06-2X-D3/def2-TZVP).

Supplementary Table 3: EDA-NOCV results (BP86-D3(BJ)/TZ2P//M06-2X/def2-SVP) on the M–M bond in **2**, Cp*Al→LiCp*, CpAl→LiCp, and dizincocene and diberyllocene analogues (energies given in kJ mol^{-1}).

	^{5}CpAl→Li^{5}Cp **2**	Cp*Al→LiCp*	CpAl→LiCp	^{5}CpZn–Zn^{5}Cp	Cp*Zn–ZnCp*	CpZn–ZnCp	^{5}CpBe–Be^{5}Cp	CpBe–BeCp
ΔE_{int}	-97.8	-59.2	-46.1	-345.1	-308.6	-303.9	-329.7	-302.8
ΔE_{Pauli}	75.6	28.7	24.9	273.5	207.6	197.9	314.3	215.0
ΔE_{disp}[a]	-65.3 (37.7 %)	-16.8 (19.1%)	-10.7 (15.0 %)	-67.7 (10.9 %)	-22.2 (4.3 %)	-15.3 (3.0 %)	-100.2 (15.6%)	-17.7 (3.4 %)
ΔE_{elst}[a]	-64.5 (37.2 %)	-43.0 (48.9 %)	-34.9 (49.2 %)	-325.5 (52.6 %)	-281.5 (54.5 %)	-271.3 (54.1 %)	-349.5 (54.3%)	-334.4 (64.4 %)
ΔE_{orb}[a]	-43.6 (25.1 %)	-28.1 (31.9 %)	-25.4 (35.8 %)	-225.4 (36.4 %)	-212.6 (41.2 %)	-215.2 (42.9 %)	-194.4 (30.2%)	-165.7 (32.0 %)
$\Delta E_{orb\text{-}\sigma}$	-26.1	-20.6	-19.9	-206.0	-201.1	-206.0	-158.0	-159.2
$\Delta E_{orb\text{-}rest}$	-17.5	-7.5	-5.5	-19.4	-11.5	-9.2	-36.3	-6.6
ΔE_{prep}	4.4	3.4	3.4	6.7	5.8	6.2	24.1	10.6
D_e	93.4	55.8	42.7	338.4	302.9	297.6	305.5	292.3
R_e	265.8	272.8	274.9	240.1	242.4	241.8	212.3	204.2

[a] The values in parenthesis give the percentage contribution to the total attractive interactions: ΔE_{elst} + ΔE_{orb} + ΔE_{disp}.

References

[1] Fulmer, G. R., Miller, A. J. M., Sherden, N. H., Gottlieb, H. E., Nudelman, A., Stoltz, B. M., Bercaw, J. E. & Goldberg, K. I. NMR Chemical Shifts of Trace Impurities: Common Laboratory Solvents, Organics, and Gases in Deuterated Solvents Relevant to the Organometallic Chemist. *Organometallics* 29, 2176-2179 (2010).

[2] a) Sheldrick, G. M. A short history of SHELX. *Acta Cryst*. A64, 112-122 (2008). b) Sheldrick, G. M. SHELXT – Integrated space-group and crystal-structure determination. *Acta Cryst*. A71, 3-8 (2015). c) Hübschle, C. B., Sheldrick, G. M. & Dittrich, B. ShelXle: a Qt graphical user interface for SHELXL. *J. Appl. Crystallogr.* 44,1281-1284 (2011).

[3] Ganesamoorthy, C., Loerke, S., Gemel, C., Jerabek, P., Winter, M., Frenking, G. & Fischer, R. A. Reductive elimination: a pathway to low-valent aluminium species. *Chem. Commun*. 49, 2858-2860 (2013).

[4] a) Dezember, T. & Sitzmann, H. Die Fulven-Route zum Pentaisopropylcyclopentadienid / The Fulvene Route to Pentaisopropylcyclopentadienide. *Z. Naturforsch. B, J. Chem. Sci.* 52, 911-918 (1997). b) Sitzmann, H. Synthese von isopropylsubstituierten cyclopentadienylliganden. *J. Organomet. Chem.* 354, 203-214 (1988).

[5] Dohmeier, C., Robl, C., Tacke, M. & Schnöckel, H. The Tetrameric Aluminum(I) Compound [{Al(η^5-C_5Me_5)}$_4$]. *Angew. Chem. Int. Ed. Engl.* 30, 564-565 (1991).

[6] Frisch, M. J., Trucks, G. W., Schlegel, H. B., Scuseria, G. E., Robb, M. A., Cheeseman, J. R., Scalmani, G., Barone, V., Mennucci, B., Petersson, G. A., Nakatsuji, H., Caricato, M., Li, X., Hratchian, H. P., Izmaylov, A.F., Bloino, J., Zheng, G., Sonnenberg, J. L., Hada, M., Ehara, M., Toyota, K., Fukuda, R., Hasegawa, J., Ishida, M., Nakajima, T., Honda, Y., Kitao, O., Nakai, H., Vreven, T., Montgomery J., J. A., Peralta, J. E., Ogliaro, F., Bearpark, M., Heyd, J. J., Brothers, E., Kudin, K. N., Staroverov, V. N., Kobayashi, R., Normand, J., Raghavachari, K., Rendell, A., Burant, J. C., Iyengar, S. S., Tomasi, J., Cossi, M., Rega, N., Millam, J. M., Klene, M., Knox, K. E., Cross, J. B., Bakken, V., Adamo, C., Jaramillo, J., Gomperts, R., Stratmann, R. E., Yazyev, O., Austin, A. J., Cammi, R., Pomelli, C., Ochterski, J. W., Martin, R. L., Morokuma, K., Zakrzewski, V. G., Voth, G. A., Salvador, P., Dannenberg, J. J., Dapprich, S., Daniels, A. D., Farkas, O., Foresman, J. B., Ortiz, J. V., Cioslowski, J. & Fox D. J. Gaussian 09, Revision C.01. Gaussian, Inc.: Wallingford CT (2009).

[7] Becke, A. D. Density-functional exchange-energy approximation with correct asymptotic behavior. *Phys. Rev. A* 38, 3098-3100 (1988).

[8] Perdew, J. P. Density-functional approximation for the correlation energy of the inhomogenous electron gas. *Phys. Rev. B* 33, 8822-8824 (1986).

[9] Becke, A. D. Density-functional thermochemistry. III. The role of exact exchange. *J. Chem. Phys.* 98, 5648-5652 (1993).

[10] Lee, C., Yang, W. & Parr, R. G. Development of the Cole-Salvetti correlation-energy formula into a functional of the electron density. *Phys. Rev. B* 37, 785-789 (1988).

[11] Adamo, C. & Barone, V. Toward reliable density functional methods without adjustable parameters: The PBE0 model. *J. Chem. Phys.* 110, 6158-6170 (1999).

[12] Ernzerhof, M. & Scuseria, G. E. Assessment of the Perdew-Berke-Ernzerhof exchange-correlation functional. *J. Chem. Phys.* 110, 5029-5036 (1999).
[13] Zhao, Y. & Truhlar, D. G. The M06 suite of density functionals for main group thermochemistry, thermochemical kinetics, noncovalent interactions, excited states, and transition elements: two new functionals and systematic testing of four M06-class functionals and 12 other functionals. *Theo. Chem. Acc.* 120, 215-241 (2008).
[14] Chai, J.-D. & Head-Gordon, M. Systematic optimization of long-range corrected hybrid density functionals. *J. Chem. Phys.* 128*,* 084106-1-084106-15 (2008).
[15] Grimme, S. Semiempirical hybrid density functional with perturbative second-order correlation. *J. Chem. Phys.* 124, 034108-1-034108-16 (2006).
[16] Goerigk, L. & Grimme, S. Efficient and Accurate Double-Hybrid-Meta-GGA Density Functionals – Evaluation with the Extended GMTKN30 Database for General Main Group Thermochemistry, Kinetics, and Noncovalent Interactions. *J. Chem. Theory Comput.* 7, 291-309 (2011).
[17] Weigend, F. & Ahlrichs, R. Balanced Basis Sets of Split Valence, Triple Zeta Valence and Quadruple Zeta Valence Quality for H to Rn: Design and Assessment of Accuracy. *Phys. Chem. Chem. Phys.* 7, 3297-3305 (2005).
[18] Grimme, S., Antony, J., Ehrlich, S. & Krieg, H. A consistent and accurate *ab initio* parametrization of density functional dispersion correction (DFT-D) for the 94 elements H-Pu. *J. Chem. Phys.* 132, 154104-1-154104-19 (2010).
[19] Grimme, S., Ehrlich, S. & Goerigk, L. Effect of the damping function in dispersion corrected density functional theory. *J. Comp. Chem.* 32, 1456-1465 (2011).
[20] Feyereisen, M., Fitzgerald, G. & Komornicki, A. Use of approximate integrals in ab initio theory. An application in MP2 energy calculations. *Chem. Phys. Lett.* 208, 359-363 (1993).
[21] Weigend, F. & Häser, M. RI-MP2: first derivatives and global consistency. *Theo. Chem. Acc.* 97, 331-340 (1997).
[22] Distasio JR., R. A., Steele, R. P., Rhee, Y. M. & Shao, Y. An improved algorithm for analytical gradient evaluation in resolution-of-the-identity second-order Møller-Plesset perturbation theory: Application to alanine tetrapeptide conformational analysis. *J. Comp. Chem.* 28, 839-856 (2007).
[23] Weigend, F., Häser, M., Patzelt, H. & Ahlrichs, R. RI-MP2: optimized auxiliary basis sets and demonstration of efficiency. *Chem. Phys. Lett.* 294, 143-152 (1998).
[24] Peng, C., Ayala, P. Y., Schlegel, H. B. & Frisch, M. J. Using redundant internal coordinates to optimize equilibrium geometries and transition states. *J. Comp. Chem.* 17, 49-56 (1996).
[25] McIver Jr., J. W. & Komornicki, A. Structure of transition states in organic reactions. General theory and an application to the cyclobutene-butadiene isomerization using a semiempirical molecular orbital method. *J. Am. Chem. Soc.* 94, 2625-2633 (1972).
[26] Reed, A. E., Weinstock, R. B. & Weinhold, F. Natural Population Analysis. *J. Chem. Phys.* 83, 735-746 (1985).
[27] Reed, A. E., Curtiss, L. A. & Weinhold, F. Intermolecular interactions from a natural bond orbital, donor-acceptor viewpoint. *Chem. Rev.* 88, 899-926 (1988).
[28] Glendening, E.D., Landis, C. R. & Weinhold, F. *NBO 7.0*: New vistas in localized and delocalized chemical bonding theory. *J. Comp. Chem.* 40, 2234-2241 (2019).

[29] Bader, R. F. W. *Atoms in Molecules: A Quantum Theory*, Clarendon, Oxford, (1990).
[30] Keith, T. A. & Gristmill, T. K. *AIMAll*, 19.02.13; Overland Park KS, USA (aim.tkgristmill.com), (2019).
[31] Andrada, D. M. & Foroutan-Nejad, C. Energy components in energy decomposition analysis (EDA) are path functions; why does it matter?. *Phys. Chem. Chem. Phys*. 22, 22459-22464 (2020).
[32] Poater, J., Andrada, D. M., Solà, M. & Foroutan-Nejad, C. Path-dependency in energy decomposition analysis & the elusive nature of bonding. *Phys. Chem. Chem. Phys*. 24, 2344-2348 (2022).
[33] Morokuma, K. Molecular Orbital Studies of Hydrogen Bond. III. C=O···H-O Hydrogen Bond in $H_2CO\cdots H_2O$ and $H_2CO\cdots 2H_2O$. *J. Chem. Phys.* 55, 1236-1244 (1971).
[34] Ziegler, T. & Rauk, A. A theoretical study of the Ethylene-Metal bond in Complexes between Cu^+, Ag^+, Au^+, Pd^0, or Pt^{2+}, and Ethylene, Based on the Hartree-Fock-Slater Transition-State Method. *Inorg. Chem.* 18, 1558-1565 (1979).
[35] Ziegler, T. & Rauk, A Carbon monoxide, carbon monosulfide, molecular nitrogen, phosphorus trifluoride, and methylisocyanide as σ donors and π acceptors. A theoretical study by the Hartree-Fock-Slater transition-state method. *Inorg. Chem.* 18, 1755-1759 (1979).
[36] Bickelhaupt, F. M., Nibbering, N. M. M., Van Wezenbeek, E. M. & Baerends, E. J. Central Bond in the Three $CN^{\cdot}$ Dimers NC-CN, CN-CN, and CN-NC: Electron Pair Bonding and Pauli Repulsion Effects *J. Phys. Chem.* 96, 4864-4873 (1992).
[37] Bickelhaupt, F. M. & Baerends, E. J. Kohn-Sham density functional theory: Predicting and understanding chemistry. In *Reviews in Computational Chemistry*, Lipkowitz, K. B. & Boyd, D. B. 15, 1-86 (2000).
[38] te Velde, G., Bickelhaupt, F. M., Baerends, E. J., Fonseca Guerra, C., van Gisbergen, S. J. A., Snijders, J. G. & Ziegler, T. Chemistry with ADF. *J. Comp. Chem.* 22, 931-967 (2001).
[39] Krijn, J. & Baerends, E. J. Fit Functions in the HFS-Method (1984).
[40] van Lenthe, E., Baerends, E. J. & Snijders, J. G. Relativistic regular two-component Hamiltonians *J. Chem. Phys.* 99, 4597-4610 (1993).
